# Drawing the tree of eukaryotic life based on the analysis of 2,269 manually annotated myosins from 328 species

**DOI:** 10.1186/gb-2007-8-9-r196

**Published:** 2007-09-18

**Authors:** Florian Odronitz, Martin Kollmar

**Affiliations:** 1 Department of NMR-based Structural Biology, Max-Planck-Institute for Biophysical Chemistry, Am Fassberg, 37077 Goettingen, Germany

## Abstract

**Background:**

The evolutionary history of organisms is expressed in phylogenetic trees. The most widely used phylogenetic trees describing the evolution of all organisms have been constructed based on single-gene phylogenies that, however, often produce conflicting results. Incongruence between phylogenetic trees can result from the violation of the orthology assumption and stochastic and systematic errors.

**Results:**

Here, we have reconstructed the tree of eukaryotic life based on the analysis of 2,269 myosin motor domains from 328 organisms. All sequences were manually annotated and verified, and were grouped into 35 myosin classes, of which 16 have not been proposed previously. The resultant phylogenetic tree confirms some accepted relationships of major taxa and resolves disputed and preliminary classifications. We place the Viridiplantae after the separation of Euglenozoa, Alveolata, and Stramenopiles, we suggest a monophyletic origin of Entamoebidae, Acanthamoebidae, and Dictyosteliida, and provide evidence for the asynchronous evolution of the Mammalia and Fungi.

**Conclusion:**

Our analysis of the myosins allowed combining phylogenetic information derived from class-specific trees with the information of myosin class evolution and distribution. This approach is expected to result in superior accuracy compared to single-gene or phylogenomic analyses because the orthology problem is resolved and a strong determinant not depending on any technical uncertainties is incorporated, the class distribution. Combining our analysis of the myosins with high quality analyses of other protein families, for example, that of the kinesins, could help in resolving still questionable dependencies at the origin of eukaryotic life.

## Abstract 2

The tree of eukaryotic life was reconstructed based on the analysis of 2,269 myosin motor domains from 328 organisms, confirming some accepted relationships of major taxa and resolving disputed and preliminary classifications.

## Background

Reconstructing the tree of life is one of the major challenges in biology [1]. Although several attempts to derive the phylogenetic relationships among eukaryotes have been published [2,3], the validity of many taxonomic groupings is still heavily debated [1]. The major reason for this is the fact that molecular phylogenies based on single genes often lead to apparently conflicting results (for a review, see [4]). Only recently has the application of genome-scale approaches to phylogenetic inference (phylogenomics) been introduced to overcome this limitation [5,6]. In this context, large and diverse gene families are often considered unhelpful for reconstructing ancient evolutionary relationships because of the accompanying difficulties in distinguishing homologs from paralogs and orthologs [7]. However, if the different homologs can be resolved, the analysis of a large gene family provides several advantages compared to a single gene analysis, because it provides additional information on the evolution of gene diversity for reconstructing organismal evolution. In addition, direct information on duplication events involving part of a genome or whole genomes can be obtained. Such an analysis requires a large and divergent gene family and sufficient taxon sampling. It is advantageous if the taxa are closely related, to provide the necessary statistical basis for subfamilies, as well as spread over many branches of eukaryotic life, to cover the highest diversity possible. Today, sequencing of more than 300 genomes from all branches of eukaryotic life has been completed [8]. In addition, many of these sequences are derived from comparative genomic sequencing efforts (for example, the sequencing of 12 *Drosophila *species), providing the statistical basis for excluding artificial relationships.

The myosins constitute one of the largest and most divergent protein families in eukaryotes [9]. They are characterized by a motor domain that binds to actin in an ATP-dependent manner, a neck domain consisting of varying numbers of IQ motifs, and amino-terminal and carboxy-terminal domains of various lengths and functions [10]. Myosins are involved in many cellular tasks, such as organelle trafficking [11], cytokinesis [12], maintenance of cell shape [13], muscle contraction [14], and others. Myosins are typically classified based on phylogenetic analyses of the motor domain [15].

Recently, two analyses of myosin proteins describing conflicting findings have been published [16,17]. Both disagree with previously established models of myosin evolution (reviewed in [18]). These analyses are based on 150 myosins from 20 species grouped into 37 myosin classes [17] and 267 myosins from 67 species in 24 classes [16], respectively. However, the number of taxa and sequences included was not sufficient to provide the necessary statistical basis for myosin classification and for reconstructing the tree of eukaryotic life.

Here, we present the comparative genomic analysis of 2,269 myosins found in 328 organisms. Based on the myosin class content of each organism and the positions of each organism's single myosins in the phylogenetic tree of the myosin motor domains, we reconstructed the tree of eukaryotic life.

## Results

### Identification of myosin genes

Wrongly predicted genes are the main reason for wrong results in domain predictions, multiple sequence alignments and phylogenetic analyses. Therefore, we have taken special care in the identification and annotation of the myosin sequences. We have collected all myosin genes that have either been derived from the isolation of single genes and submitted to the nr database at NCBI, or that we obtained by manually analysing the data of whole genome sequencing and expressed sequence tag (EST)-sequencing projects. Gene annotation by manually inspecting the genomic DNA sequences was the only way to get the best dataset possible because the sequences derived by automatic annotation processes contained mispredicted exons in almost all genes (for an in-depth discussion of the problems and pitfalls of automatic gene annotation, gene collection, domain prediction and sequence alignment, see Additional data file 1). These predicted genes contain errors derived from including intronic sequence and/or leaving out exons, as well as wrong predictions of start and termination sites. Automatic gene prediction programs are also not able to recognize that parts of a gene belong together if these are spread over two or several different contigs. Often they also fail to identify all homologs in a certain organism. The only way to circumvent these problems is to perform a manual comparative genomic analysis. In addition, datasets with automatically predicted model transcripts are available for only a small part of all sequenced genomes.

The basis of our analysis was a very accurate multiple sequence alignment. In cases of less conserved amino acid stretches, the corresponding DNA regions of several organisms have been analyzed in parallel, aiming to identify coding regions and shared intron splice sites. Thus, our dataset was generated by an iterative gene identification (using TBLASTN) and gene annotation process, meaning that most of the myosin sequences have been reanalyzed as soon as data from closely related organisms or further species specific data (new cDNA/EST data or a new assembly version) became available. In addition to manually annotating the myosins from genomic data, it was also absolutely necessary to reanalyze previously published data, as these also contain many sequencing errors (especially sequences produced in the last century) and wrongly predicted translations.

The myosin dataset contains 2,269 sequences from 328 organisms (Table 1), of which 1,941 have been derived from 181 whole genome sequencing (WGS) projects. Of all myosin sequences, 1,634 are complete (from the amino terminus to the carboxyl terminus) while parts of the sequence are missing for 635. Sequences for which a small part is missing (up to 5%) were termed 'Partials' while sequences for which a considerable part is missing were termed 'Fragments'. This difference has been introduced because Partials are not expected to considerably influence the phylogenetic analysis. Indeed, even long loops like the approximately 300 amino acid loop-1 of the Arthropoda variant C class-I myosins can either be included or excluded from the analysis without changing the resulting trees (data not shown). Eight of the myosins were termed pseudogenes because they contain proven single frame shifts in exons (for example, in the *Hs*Mhc20 gene) or many frame shifts and missing sequences that cannot be attributed to sequencing or assembly errors.

**Table 1:** Data statistics

Sequences	2,269	Total
	1,941	From WGS
	1,634	Complete sequences
	38	Domains
	3,441,237	Amino acids
	8	Total pseudogenes
	2	Pseudogenes without sequence
		
Classes	35	Classes
	149	Unclassified myosins
		
Motor domain position	1,806	Amino-terminal
	1	Carboxy-terminal
	305	Middle
	157	Unknown
		
Completeness	1,834	Heads complete
	150	Head partials
	277	Head fragments
	149	Only head sequence
	6	Only tail sequence
	1,725	Tails complete
	183	Tail partials
	210	Tail fragments
		
Extremes	4,407	Amino acids in *Br*Myo15B*
	495	kDa is the weight of *Br*Myo15B*
	61	Myosin homologs in Br*
	23	Homologs for *Ol*Mhc*
	13	Classes in *Br*, *Dap*, *Gg*, Xt*
		
Species	328	Total
	181	WGS-projects
	127	EST-projects
	80	WGS- and EST-projects
	3	Species without myosin heavy chain

*Br, *Brachydanio rerio*; *Ol*, *Oryzias latipes*; *Dap*, *Daphnia pulex*; *Gg*, *Gallus gallus*; *Xt*, *Xenopus tropicalis*.

**Figure 1:**
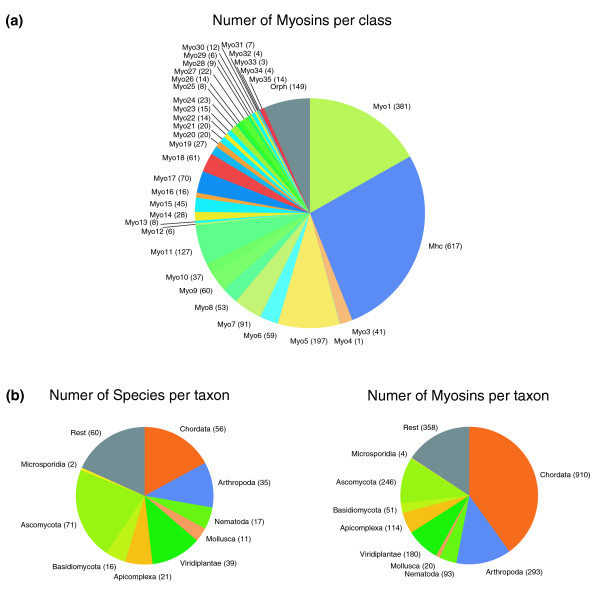
Taxon and class related statistics of the myosin dataset. **(a) **The pie-chart shows the number of myosins for each class. **(b) **The charts show the number of species and the number of myosins for a set of selected taxa. Exact numbers are given in brackets.

Class-I and class-II by far comprise the most myosins (Figure 1a). Class-I myosins were found in almost all organisms, and class-II myosins have undergone several gene duplications (either resulting from whole genome or single gene duplications), leading to up to 22 class-II myosins per vertebrate organism. Although the total numbers of myosins per class are biased by the sequenced species, we expect class-I and class-II to remain the largest classes even if many other species not containing any of these classes (for example, the plants and Alveolata) are sequenced in the future (Figure 1b). For example, the numbers of species of the Chordata and the Viridiplantae lineage for which myosin data are available are similar. However, the number of myosins for each of these species is very different, with the Chordata species encoding up to three times more myosins. In contrast, the number of sequenced Fungi species (over 90 organisms) is almost twice as high as the number of Chordata species, but the number of Fungi myosins is only a quarter of that of the Chordata myosins.

### Nomenclature

The amount of produced data spread over all eukaryotic kingdoms now allows and demands a consistent, systematic, and extendable nomenclature. Here, we introduce the following nomenclature, which builds on the already established system [15,18-20] and tries to keep as many of the existing names as possible. Nevertheless, it changes some of the already used names, thus getting rid of sequence-specific and species-specific exceptions. We are aware of the confusion that this might introduce about the names of some sequences, but given the fact that the amount of annotated data known before finishing this analysis (about 250-300 sequences) was very small compared to the data presented here, it was necessary for us to introduce an appropriate nomenclature. Otherwise the number of exceptions would soon exceed the number of consistently named sequences. We are also aware that different names and classifications have recently been introduced in the literature [16,17]. However, these results were derived from analyses of small datasets based on many incorrectly assembled sequences and, thus, wrongly annotated myosins, and we have not found a way to incorporate the small part of matching data into our system. We also think that even if we introduce some confusion to certain researchers in the field, there is a strong necessity to have an appropriate nomenclature to manage existing and upcoming data. CyMoBase, which we have developed to provide access to all myosin sequence data [21], uses the new nomenclature, provides links to previously used names, and can be used as reference.

The nomenclature is simply as follows and in agreement with what most people in the field already use. The names of the sequences consist of four parts: the abbreviation of the species' systematic name; the abbreviation of the protein; the class designation; and the variant designation.

#### Abbreviation of the species' systematic name

In general, species are abbreviated by using the first letters of their systematic names (for example, *Dm *for *Drosophila melanogaster*). However, there are many species, that would have the same abbreviation, and in these cases we added the second letter of the first part of the name (for example, *Drm *for *Drosophila mimetica*). Different strains of the same species are differentiated by adding lowercase letters separated by an underscore (for example, *Pf_a *for *Plasmodium falciparum 3D7*, *Pf_b *for *Plasmodium falciparum Ghanaian Isolate*, *Pf_c *for *Plasmodium falciparum HB3*, *Pf_d *for *Plasmodium falciparum Dd2*).

#### Abbreviation of the protein

The abbreviation of the protein is Myo. In the case of the class-II myosins, the abbreviations Mhc and Mys are used in the literature. As class-II comprises by far the most sequences and as numbers have very often been introduced as variant designations (for example, human Mys1, Mys2, and so on), we decided to keep the class-II abbreviation as an exception of the proteins general abbreviation. We decided to use Mhc as protein abbreviation for class-II myosins as the abbreviation Mys has been used only for mammalian members while all other class-II myosins have been named Mhc. If the class-II myosins were named Myo2 (in accordance with the other myosin classes) we would have to also rename their variant designations to avoid confusion with other classes (for example, Myo21 could be a class-II myosin variant 1 or a class-XXI myosin).

#### Class designation

Classes are numbered according to their discovery. Thus, we keep all previously accepted class designations [18]. Recent further class designations [16,22] are based on data analyses of very small datasets of wrongly annotated myosins and will not be considered. Richards and Cavalier-Smith [17] have also used wrongly annotated myosins in their analysis and have developed a completely new classification not consistent with any previous classification. As has been agreed upon in the past, new classes should be designated only if members of different organisms contribute. We have been very conservative in our analysis in designating new classes, assigning new classes only if several species contribute (for example, class-XXI, all Arthropoda), or very divergent species contribute (for example, class-XXIX, *Thallassiosira pseudonana*, *Phytophthora *sp. and others), or, if the species are closely related, several homologs of each species contribute (for example, class-XXX, *Phytophthora *sp. and *Hyaloperonospora parasitica*). It is obvious, that class separation improves as more and more divergent sequences are added. In particular, the myosins of very divergent species (for example, *Phytophthora *sp., *Thallassiosira pseudonana*, *Tetrahymena thermophila*, *Paramecium tetrarelia*) tend to group mainly with the homologs of the same organism. Our experience showed that if more sequences of closely related species are added (for example, sequences of *Phytophthora ramorum*, *Phytophthora infestans*, and *Phytophthora sojae*), the class separation improves, and improves further if sequences of more divergent species are added (*Hyaloperonospora parasitica*). But in most of these cases the separation is still not good enough to distinguish between a class separation and just a variant separation. Thus, we designated only classes that are well-supported and separated. There are 24 classes supported by bootstrap values higher than 985 (out of 1,000; Additional data file 2) and 5 are supported by bootstrap values higher than 874. Class-I has the widest taxonomic distribution and is supported by a bootstrap value of 788. Class-XXVIII (bootstrap value of 750), class-V (593) class-XXIII (463) and class-XV (305) show the lowest bootstrap values, but are well separated from any neighboring class. We left groups of sequences (for example, the *Tetrahymena thermophila *and *Paramecium tetrarelia *myosins) unclassified, although their first node in the tree might be supported by a relatively high bootstrap value. A similar situation would exist if only five sequences of class-VII, class-X, and class-XV myosins were known; in this case, these sequences would certainly group together, supported by a high bootstrap value of the first node, as they are far more similar to each other than to the other myosins. Adding more homologs showed these myosins to be separated into three classes, and we expect a similar class separation for the myosins of, for example, *Tetrahymena thermophila *and *Paramecium tetrarelia *if more sequences of closely related species are added.

#### Variant designation

If several myosin homologs exist for the same class, they are distinguished by a variant designation, a letter starting with A. Variants with numbers may be used only for the class-II myosins (see above).

#### Additional qualification

If both alleles of an organism have been assembled independently, providing two versions for each myosin gene, the different versions are distinguished by adding alpha and beta to the sequence name. Alternative splice forms of the same gene get the same protein name. All myosins that cannot be classified at the moment will be considered as 'orphan' myosins. If several orphans exist in a species, they get a variant designation. Orphan names are considered to be preliminary names. Thus, orphan myosins will be renamed as soon as more sequences are available that allow a well-supported classification.

### Classification

The basis for the classification of the myosins is the phylogenetic relation of their myosin motor domains [15,18]. The data for the myosins is now strong enough that all designated classes are well supported. Including or excluding sets of myosins (for example, the orphans) does not change the phylogeny of the other classes as has been observed for the small dataset used in previous analyses [16]. Also, including or excluding large insertions like the loop-1 insertion of the class-I variant C myosins of Arthropoda does not change the tree.

In contrast to other suggestions, we do not agree with the idea that the tail domain architectures should also be considered in the classification process [16,17]. Our analysis shows that the motor domains and the tails coevolved in most of the assigned classes, but there are many exceptions now where the separation of organismal lineages occurred before the adaptation of further tail domains. It does not make sense to artificially 'force' sequences together only because there is not enough sequence data for a better classification. If, for example, the class-XII myosins should be related to the class-XV myosins only because they also contain MyTH4 and Ferm domains [16], then they could also be grouped with the class-VII, class-X, or class-XXII myosins. Many other myosins from Stramenopiles or Amoeba would also have to be grouped with these classes as they also contain MyTH4 and Ferm domains. This seems very arbitrary. Also, several domains, such as the PH domain, Ankyrin repeats or the Pkinase domain, are found on either the amino terminus or the carboxyl terminus of the myosins. Many of the tail regions have also not been analyzed specifically (domains have not been defined yet). Thus, as soon as further domains are defined other myosin classes might unexpectedly share tail regions. It is also not reasonable to consider the organismal distribution of myosins as a classification helper as has been proposed [16]. The species sequenced cover only an extremely small part of all organisms, and their selection has also been biased in favor of financial, medical and other interests. It is not reasonable, therefore, to assume that the organisms that we have data for are the best representatives with regard to the myosin diversity of their taxa. For example, even the well-studied *Drosophila melanogaster *has lost the class-XXII myosin that the closely related species *Drosophila willistoni *and other *Drosophila *species still have. Other Arthropoda (*Daphnia*, *Apis*, *Anopheles*) have additional myosins belonging to well established classes (for example, a class-III myosin and a class-IX myosin) that all *Drosophila *species (that have been sequenced so far) have lost. The same is true for nematodes, where a class-XVIII myosin is found in *Brugia malayi *and not in *Caenorhabditis *species. It is very unlikely, therefore, that myosins that do not group to any of the other assigned metazoan myosins (for example, the class-XII myosins) are closely related to one of the metazoan classes, although they might share some domains in the tail regions. It is far more likely that a class-XII myosin will be found in another metazoa species (as, for example, a class-XX myosin has been found in Echinodermata in addition to Arthropoda), or that a class-XV myosin, to which the class-XII myosins have artificially been grouped [16], will be found in another nematode (as, for example, a class-XVIII myosin has been found in *Brugia malayi*). Both possibilities will support the current class designation. Nevertheless, at the moment it seems that all sequenced lineages have developed their own specific myosin, for example, the class-XVI myosins in vertebrates, the class-XXI myosins in Arthropoda, and the class-XII myosins in Nematoda.

Fragments have been classified and named based on their obvious homology at the amino acid level. Those Fragments that did not obviously group to one of the assigned classes have sequentially been added to the dataset used to construct the major tree. Some of these Fragments could subsequently be classified; others have to be considered as orphans. Note that even very short fragments of only 100 amino acids are sufficient for proper classification. Thus, it is very unlikely that the orphan Fragments will group to one of the established 35 classes if their full-length sequences become available.

### Renamed myosins

#### Change of previous classification

Class-IV contains only one myosin. According to the nomenclature guidelines outlined above, this myosin would not be designated as a class but would be considered as an orphan. So as not to cause confusion, we did not change its classification from class-IV myosin, expecting that more members will be added as soon as further genomes are sequenced. However, our phylogenetic tree shows that the former class-XIII myosins (of the algae *Acetabularia cliftonii*) belong to the class-XI myosins, supported by a bootstrap value of 999. Therefore, we reclassified the former *Acetabularia *class-XIII myosins as class-XI myosins, and assigned the class-XIII to a Kinetoplastida specific myosin class. The *Drosophila melanogaster *NinaC protein has previously been classified as a class-III myosin. However, other Arthropoda contain real class-III myosins (or more precisely, homologs to the mammalian class-III myosins) and NinaC as well as the NinaC homologs of the other Arthropoda form a distinct class. We decided not to rename all the mammalian class-III myosins but to rename NinaC and introduce the new class-XXI.

#### Change of previous names

The apicomplexan myosins have traditionally been named alphabetically [16,23]. However, even different splice forms of the same gene received different protein names. In addition, gene and genome duplication events have led to, and will continue to lead to, confusing naming. Thus, it is not possible to name these myosins consistently in an alphabetical manner and to provide consistency for the future. We renamed the apicomplexan myosins according to our nomenclature, introducing some apicomplexan-specific myosin classes. Nevertheless, we tried to keep the former letters as variants where possible.

The *Saccharomyces cerevisiae *myosins have previously been named numerically [24], thus leading to confusion with class numbers. In addition, several yeast species have now been sequenced that separated before some of the gene and whole genome duplication events happened during yeast evolution. Most of the sequenced yeast species contain only one version of the class-I and class-V myosins, and *Naumovia castellii *contains one class-I but two class-V myosins. It is not possible to name the newly identified yeast myosins according to the *Saccharomyces cerevisiae *myosins. Therefore, we renamed the *Saccharomyces cerevisiae *myosins according to our nomenclature.

Some of the plant and algae myosins were given arbitrary names in the past, especially those from *Helianthus annuus *and *Arabidopsis thaliana*. This happened before genome data became available but has not been changed since [25]. We have renamed these few myosins. Some of the vertebrate class-II myosins have also been renamed based on their homology to myosins from closely related organisms. In particular, descriptive names (for example, 'nonmuscle myosin II' or 'fast skeletal muscle myosin') have been disbanded in favor of numerical variant designations as suggested [18].

### Thirty-five myosin classes

**Figure 2:**
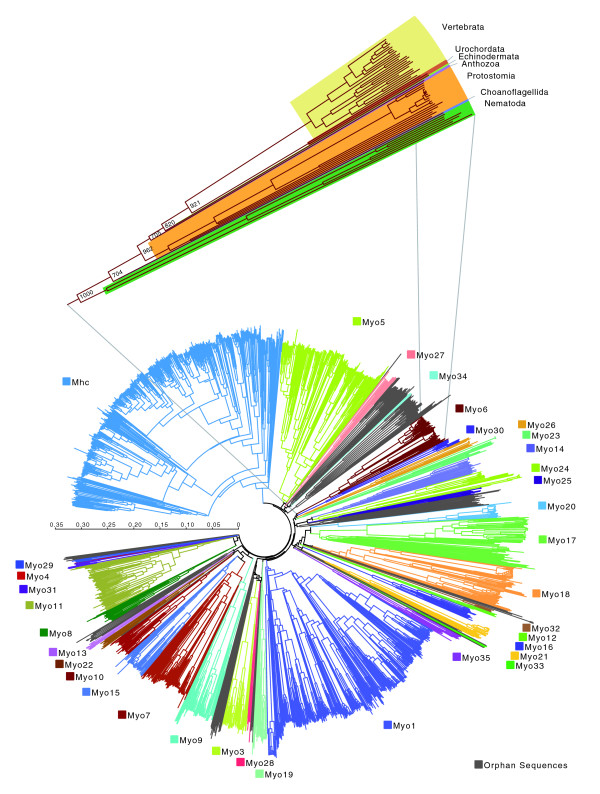
Phylogenetic tree of the myosin motor domains. The phylogenetic tree was built from the multiple sequence alignment of 1,984 myosin motor domains. The complete tree with bootstrap values and sequence descriptors is available as Additional data file 2. The expanded view shows the myosin sequences of class-VI and their distribution in taxa. Every other myosin class has been analyzed in a similar way. Labels at branches are bootstrap values (1,000 total boostraps). The scale bar corresponds to estimated amino acid substitutions per site. The tree was drawn using FigTree v1.0 [40].

**Figure 3:**
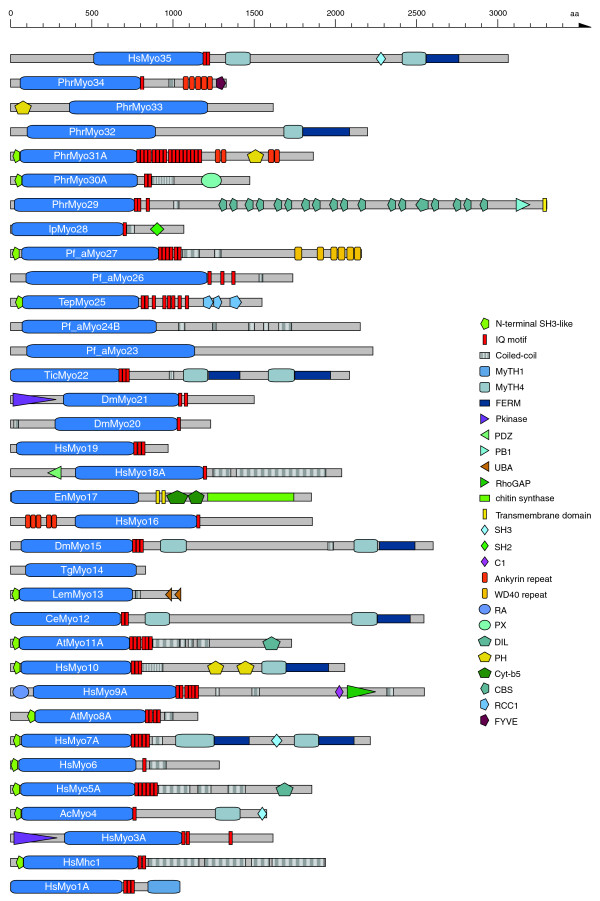
Schematic diagram of the domain structures of representative members of the 35 myosin classes. The sequence name of the representative member is given in the motor domain of the respective myosin. A color key to the domain names and symbols is given on the right except for the myosin domain, which is colored in blue. The abbreviations for the domains are: C1, protein kinase C conserved region 1; CBS, cystathionine-beta-synthase; Cyt-b5, cytochrome b5-like Heme/Steroid binding domain; DIL, *dilute*; FERM, band 4.1, ezrin, radixin, and moesin; FYVE, zinc finger in Fab1, YOTB/ZK632.12, Vac1, and EEA1; IQ motif, isoleucine-glutamine motif; MyTH1, myosin tail homology 1; MyTH4, myosin tail homology 4; PB1, Phox and Bem1p domain; PDZ, PDZ domain; PH, pleckstrin homology; Pkinase, protein kinase domain; PX, phox domain; RA, Ras association (RalGDS/AF-6) domain; RCC1, regulator of chromosome condensation; RhoGAP, Rho GTPase-activating protein; SH2, *src *homology 2; SH3, *src *homology 3; UBA, ubiquitin associated domain; WD40, WD (tryptophan-aspartate) or beta-transducin repeats.

The analysis of the phylogenetic tree of the 2,269 myosin motor domain sequences resulted in the definition of 35 myosin classes (Figures 2 and 3; Additional data file 2), of which 19 classes have been assigned and described previously [18]. Our analysis supports and retains the existing classification except for the former class-XIII, which consisted of two myosins from the chlorophyte *Acetabularia peniculus *(*Acetabularia cliftonii*). The former class-XIII was substituted by a Kinetoplastide-specific class consisting of myosins with an amino-terminal SH3-like domain, a coiled-coil region, and two tandem UBA domains. Five new classes, class-XX, class-XXI, class-XXII, class-XXVIII, and class-XXXV, are specific to Metazoan species. So far, class-XX has been found only in arthropods and the sea urchin *Strongylocentrotus purpuratus *and consists of myosins with a long, coiled-coil region containing an amino-terminal domain and a short neck composed of one IQ motif. The myosins of class-XXI are very similar to the class-III myosins in their domain organization but contain distinct motor domains. The class-XXII myosins are defined by two tandem MyTH4 and FERM domains. Most Metazoan species have lost their class-XXVIII myosin. So far, class-XXVIII myosins have been identified only in the sea anemone *Nematostella vectensis*, the frog *Xenopus tropicalis*, *Gallus gallus*, and some fishes. From the data available it seems that the species of the Acanthopterygii branch of the fishes (including *Takifugu rubripes *and *Gasterosteus aculeatus*) have lost the class-XXVIII myosins. The tail regions of class-XXVIII myosins consist of an IQ motif, a short coiled-coil region and an SH2 domain.

Five of the new myosin classes (class-XXIII to class-XXVII) are composed solely of Apicomplexan myosins. The domain organizations of these myosins have been described elsewhere [16] but classes have not been assigned yet. Another six new myosin classes were attributed to Stramenopiles myosins (class-XXIX to class-XXXIV). Class-XXIX shows the highest taxonomic sampling, consisting of members from all Stramenopiles species. Class-XXIX myosins have very long tail domains consisting of three IQ motifs, short coiled-coil regions, up to 18 CBS domains, a PB1 domain, and a carboxy-terminal transmembrane domain. The myosin classes XXX to XXXIV contain only members from *Phytophthora *species and the closely related *Hyaloperonospora parasitica*. Although the taxonomic sampling is quite low, these classes have distinct motor domains and unique tail domain organizations. Myosins of class-XXX are composed of an amino-terminal SH3-like domain, two IQ motifs, a coiled-coil region and a PX domain. Class-XXXI myosins have a very long neck region consisting of 17 IQ motifs and two tandem Ankyrin repeats separated by a PH domain. Class-XXXII myosins do not contain any IQ motifs but a tandem MyTH4 and FERM domain. The myosins of class-XXXIII have long amino-terminal regions with an amino-terminal PH domain. Class-XXXIV myosins are composed of one IQ motif, a short coiled-coil region, five tandem Ankyrin repeats, and a carboxy-terminal FYVE domain.

### Orphan myosins

#### Fungi/Metazoa lineage

**Figure 4:**
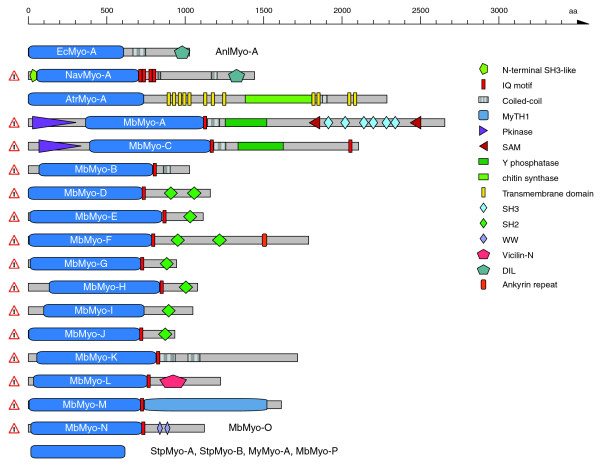
Schematic diagram of the domain structures of the orphan myosins of the Fungi/Metazoa lineage. The sequence names of the ophan myosins are given in the motor domain of the respective myosins. Color keys to the domain names and symbols are given on the right except for the myosin domain, which is colored in blue. Myosin names next to domain representations list orthologs from closely related species or orthologs from the same species. These sequences have a similar domain organization. Sequences that are not orthologs and have not resulted from recent gene duplications are shown separately, although their domain organizations might be very similar. The myosin domains without names on the bottom symbolize that only head fragments are available for the sequences listed on the right. The exclamation mark on the left side of some sequences signifies that the corresponding sequences (especially the tail regions) have not completely been validated because of missing comparative genome sequences. Those sequences and corresponding tail domain predictions might change with upcoming genome sequences of related species. Abbreviations for the domains are: SAM, sterile alpha motif; Vicilin-N, Vicilin amino-terminal region; WW, tryptophan-tryptophan motif domain; Y phosphatase, protein tyrosine phosphatase, catalytic domain.

The domain organizations of the orphan myosins of the Fungi/Metazoa lineage are shown in Figure 4. The Microsporida have two myosins, one class-II myosin and an orphan myosin containing a DIL domain that is also shared by class-V and class-XI myosins. In contrast to these classes, the Microsporida orphan myosins do not have any IQ motifs, thus lacking the ability to bind calmodulin-like light chains. The wasp *Nasonia vitripennis *has an orphan myosin that has a similar domain organization to the class-V and class-XI myosins, although it has less IQ motifs and its coiled-coil region is considerably shorter. This myosin is unique to all Arthropoda species sequenced so far. A myosin very similar in domain organization to the fungal class-XVII myosins has been found in the mollusc *Atrina rigida*. It has 12 transmembrane domains separated by a chitin synthetase domain. The choanoflagellate *Monosiga brevicollis *has 16 orphan myosins of different domain organizations. Due to missing genome sequence data of closely related species, all these gene predictions are preliminary (especially the tail regions) and might change in the future. Some of the predicted orphan myosins contain domains unique to all myosins analyzed so far, like the SAM and the Vicilin-N domains. Seven sequences contain SH2 domains as have been found in the class-XXVIII myosins.

#### Alveolata lineage

**Figure 5:**
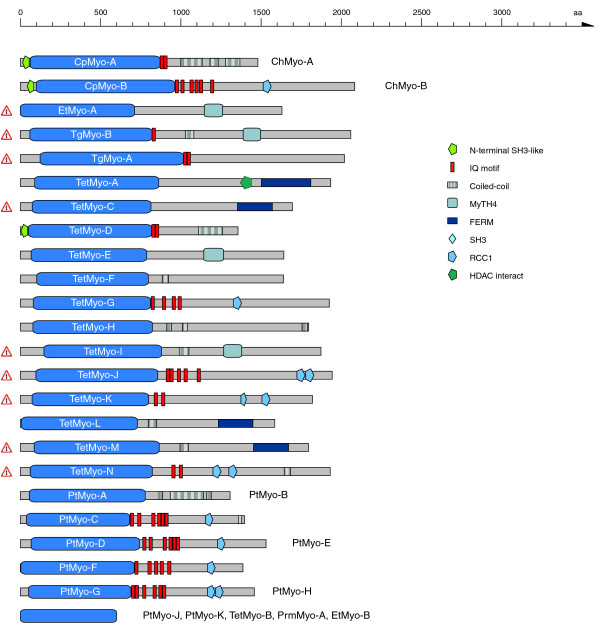
Schematic diagram of the domain structures of the orphan myosins from the Alveolata lineage. The sequence names of the ophan myosins are given in the motor domain of the respective myosins. Color keys to the domain names and symbols are given on the right except for the myosin domain, which is colored in blue. Myosin names next to domain representations list orthologs from closely related species or orthologs from the same species. These sequences have a similar domain organization. Sequences that are not orthologs and have not resulted from recent gene duplications are shown separately, although their domain organizations might be very similar. The myosin domains without names on the bottom symbolize that only head fragments are available for the sequences listed on the right. The exclamation mark on the left side of some sequences signifies that the corresponding sequences (especially the tail regions) have not completely been validated because of missing comparative genome sequences. Those sequences and corresponding tail domain predictions might change with upcoming genome sequences of related species. Abbreviations for the domains are: HDAC interact, histone deacetylase (HDAC) interacting.

Several of the Alveolata myosins could not be classified (Figure 5). All *Tetrahymena thermophila *and *Paramecium tetraurelia *myosins remain ungrouped. The tails of the *Paramecium tetraurelia *myosins contain only IQ motifs, coiled-coil regions, and RCC1 domains, while some of the *Tetrahymena thermophila *myosins also contain FERM or MyTH4 domains. However, the FERM and MyTH4 domains never appear in tandem like in class-VII, class-X, or class-XXII myosins.

#### Orphan myosins from Stramenopiles

**Figure 6:**
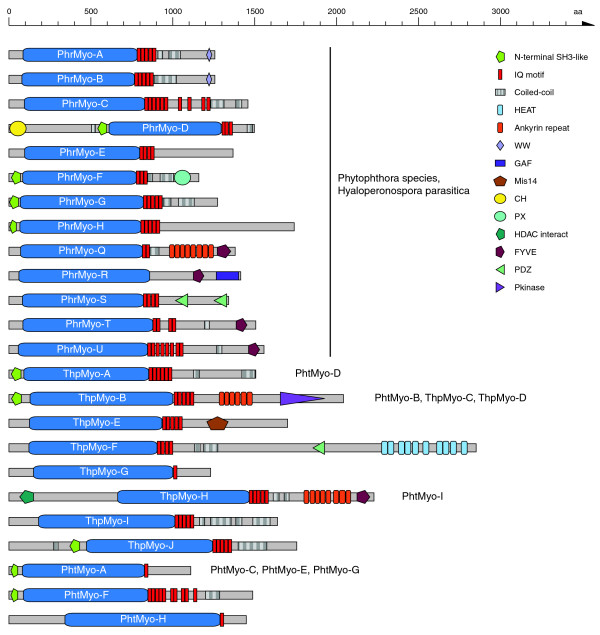
Schematic diagram of the domain structures of the orphan myosins from Stramenopiles. The sequence names of the ophan myosins are given in the motor domain of the respective myosins. Color keys to the domain names and symbols are given on the right except for the myosin domain, which is colored in blue. Myosin names next to domain representations list orthologs from closely related species or orthologs from the same species. These sequences have a similar domain organization. Sequences that are not orthologs and have not resulted from recent gene duplications are shown separately, although their domain organizations might be very similar. Abbreviations for the domains are: CH, Calponin homology domain; GAF, domain present in phytochromes and cGMP-specific phosphodiesterases; HEAT repeat, named after the proteins huntingtin, elongation factor 3 (EF3), the 65 kDa alpha regulatory subunit of protein phosphatase 2A (PP2A) and the yeast PI3-kinase TOR1; Mis14, kinetochore protein Mis14 like.

Although they share only the class-I myosins, the Stramenopiles species show a similar myosin diversity as the metazoan species (Figure 6). So far, three *Phytophthora *species and the closely related *Hyaloperonospora parasitica *have been sequenced; all share the same set of myosins. The orphan myosins of this group have not been classified because it is not clear from the phylogenetic tree where to draw class boundaries. However, it is obvious that the Myo-A to Myo-H and the Myo-Q to Myo-U orphans form distinct groups. The domain organizations of the myosins within these groups are also very different. To resolve their classification, further data from more distantly related species are needed. The genome sequences of two diatoms, *Phaeodactylum tricornutum *and *Thalassiosira pseudonana*, have also been finished. Both species share several sequences, but *Thalassiosira pseudonana *has a higher myosin diversity, having myosins with HEAT or Mis14 domains that do not exist in any other myosin.

#### Orphan myosins from other taxa

**Figure 7:**
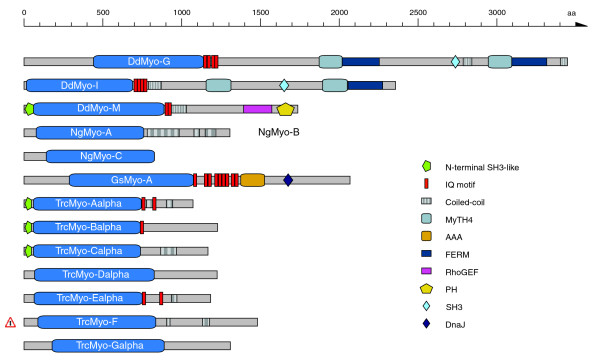
Schematic diagram of the domain structures of the orphan myosins of species not belonging to one of the other taxa. Both alleles of *Trypanosoma cruzi *have been assembled independently, providing two slightly different copies of each myosin gene. None of the Myo-F versions is complete and the presented domain organization of Myo-F is the result of a merged version of both myosins. The sequence names of the ophan myosins are given in the motor domain of the respective myosins. Color keys to the domain names and symbols are given on the right except for the myosin domain, which is colored in blue. Myosin names next to domain representations list orthologs from closely related species or orthologs from the same species. These sequences have a similar domain organization. Sequences that are not orthologs and have not resulted from recent gene duplications are shown separately, although their domain organizations might be very similar. Abbreviations for the domains are: AAA, ATPase family associated with various cellular activities; DnaJ domain, named after the prokaryotic heat shock protein DnaJ; RhoGEF, Rho GDP/GTP exchange factor.

Orphan myosins from other taxa are shown in Figure 7. The *Dictyostelium discoideum *orphan myosins have been discussed elsewhere [26]. The amoeba-flagellate *Naegleria gruberi *has three orphan myosins having only coiled-coil regions in the tail. The unicellular red alga *Galdieria sulphuraria *contains one myosin with a unique domain organization consisting of at least nine IQ motifs followed by an AAA domain and a DnaJ domain. Both alleles of *Trypanosoma cruzi *have been assembled independently, providing two slightly different versions for each myosin gene. The seven orphan myosins of *Trypanosoma cruzi *contain amino-terminal SH3-like domains, IQ motifs, or coiled-coil regions.

### Species that do not contain myosins

There are three species whose genome sequences are available and that do not contain any myosin: the unicellular red alga *Cyanidioschyzon merolae*, the flagellated protozoan parasite *Giardia lamblia*, and the protozoan parasite *Trichomonas vaginalis*.

## Discussion

All myosin protein sequences have been derived by manually inspecting the corresponding DNA, either the published cDNA or genomic DNA, or the genomic DNA provided by sequencing centers. Published sequences contained errors in many cases, either from sequencing or from manual annotation, while automatic annotations provided by the sequencing centers resulted in mispredicted exons in almost all transcripts. For many sequences, the prediction of the correct exons was only possible with the help of the analysis of the homologs of related species. Thus, not only has the quantity of myosin data increased as more and more genomes have been analyzed but also the quality as all ambiguous regions could be resolved for those sequences for which data from a closely related organism are available. Therefore, mispredicted exons may be limited to a few orphan myosins.

For the phylogenetic analysis of the myosin motor domains we created a structure-guided manual sequence alignment whose quality is far beyond any computer-generated alignment. It is obvious that all secondary structure elements of the class-II myosin motor domain structure remain conserved in all myosins, even in the most divergent homologs. Sequence motifs that would not have been aligned at first glance were placed based on the analysis of their supposed three-dimensional counterparts, which always maintained the structural integrity of the respective region. Thus, strong sequence variation and sequence insertions were limited to loop regions. Based on the phylogenetic tree constructed from 1,984 myosin motor domains, 35 classes have been assigned (Figures 2 and 3; Additional data files 2 and 3). There are 149 myosins that still remain unclassified due to our conservative view on designating classes but it is anticipated that sequencing of further genomes will result in their classification and will substantially increase the existing number of classes. For generating the tree it does not matter whether long loop regions (for example, the 300 amino acid loop-1 of the Arthropoda Myo1C proteins) are included in the alignment or not (data not shown). So far, almost all orphan myosins belong to taxa that have not undergone large-scale comparative sequencing efforts. Only short sequence fragments have been found for 277 myosins. These sequences were excluded from the phylogenetic analysis but have been classified based on their similarity in the multiple sequence alignment. Nevertheless, these data are important for defining myosin diversity in as many organisms as possible.

The highest number of myosins in a single organism has been found in *Brachydanio rerio *(61 myosins grouped into 13 classes) while the broadest class distribution is expected for the Phytophthora species (25 myosins grouped into at least 15 classes). The high numbers of vertebrate myosin genes in general are due to several whole genome duplications that happened after the separation from the Craniata and Urochordata [27].

**Figure 8:**
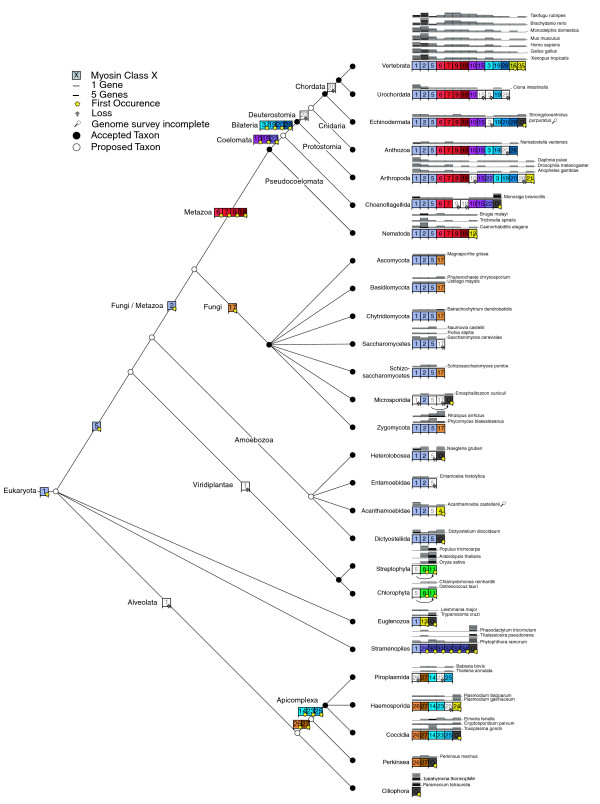
Schematic drawing of the evolution of myosin diversity. The tree has been constructed based on the combination of the phylogenetic information obtained from the analysis of single myosin classes as well as the analysis of the class distribution of major taxa (see Materials and methods). Thus, branch lengths do not correspond to any scale. Nodes that have already been suggested are symbolized by filled circles. Nodes that we propose base on the analysis of the myosins are represented by open circles. The exact myosin contents of several representative organisms are given. The myosin inventory of all 328 organisms is available from Additional data file 3.

Our survey of the myosin gene family now allows the reconstruction of the tree of 328 eukaryotes (Figure 8). The organisms of the major clades Fungi/Metazoa, Euglenozoa, Stramenopiles and Alveolata have distinct sets of myosin classes (except class-I), showing that horizontal gene transfer of myosins has not happened in later stages of eukaryotic evolution. However, we cannot exclude yet that horizontal gene transfer of myosins has not happened at the origin of eukaryotic evolution. Hence, only paralogs and orthologs have to be resolved. Figure 8 represents a schematic reconstruction of both the phylogenetic relationships of major taxa reconstructed from class-specific trees as well as the information on myosin class evolution and distribution. For example, *Tetrahymena thermophila*, *Perkinsus marinus*, *Toxoplasma gondii*, *Plasmodium falciparum*, and *Babesia bovis *have all been classified as Alveolata. However, the relation between Ciliophora (*Tetrahymena thermophila*), Perkinsea (*Perkinsus marinus*), and Apicomplexa (*Toxoplasma gondii*, *Plasmodium falciparum*, and *Babesia bovis*) has not been resolved yet. *Tetrahymena thermophila *does not share any myosin with the other Alveolata and should, therefore, have diverged before the other species. *Perkinsus marinus *shares two myosin classes with the Apicomplexa. Thus, they must have had a common ancestor. The Apicomplexa developed three further common classes, of which single classes have been lost by different species. The myosin class-specific trees show that the Coccidia, the Haemosporida, and the Piroplasmida form distinct lineages. However, their relation cannot be resolved further. This principle for reconstructing the tree has been applied to all species.

The class-I myosins show the widest taxonomic distribution and are devoid of the amino-terminal SH3-like domain and are thus suggested to be the first myosins to have evolved (see below). Only two major lineages, the Viridiplantae and the Alveolata, do not contain class-I myosins (Figure 8). The Alveolata have either lost the class-I myosin, or their class-I myosin diverged so far that a common ancestor could not be reconstructed. The Apicomplexa developed several specific classes, while the Ciliophora myosins cannot be classified yet. The evolutionary history of the Euglenozoa and Stramenopiles cannot be further resolved because both do not share any further myosin classes with other species, and their taxonomic sampling is not high enough for a more precise grouping.

The second myosin class to develop during the evolution of the Fungi and Metazoa kingdoms was class-V. The plants have developed two kingdom-specific classes. However, the domain organization of the plant-specific class-XI is similar to that of class-V, suggesting that both had a common ancestor. In contrast to the class-I myosins, the class-V and class-XI myosins have diverged so far that a common ancestry is not visible beyond their general domain organization. After separation of the plant lineage, the class-II myosins arose. The protists *Entamoeba *sp., *Acanthamoeba castellanii*, *Naegleria gruberi*, and *Dictyostelium discoideum *have closely related myosins, suggesting that they share a common ancestor that diverged shortly before the Fungi and Metazoa split. While the Entamoebidae have lost their class-V myosin, retaining only a class-I and a class-II myosin, the Acanthamoebidae, Dictyosteliida, and Heterolobosea have developed several additional specific myosins with unique domain organizations, in addition to the increase in the number of myosin genes through single gene or whole genome duplications. The Acanthamoebidae and Dictyosteliida already contain the combination of the myosin motor domain and the MyTH4 domain that is also widely found in the metazoan lineage. However, a lack of genomic data prevents the designation of a common myosin motor domain-MyTH4 containing ancestor. The fungi developed the class-XVII myosin that consists of a functionally restricted myosin motor domain fused with a highly conserved chitin synthetase [28]. While the Ascomycetes, Basidiomycetes, and Chytridiomycota have retained one member of each of the four myosin classes, the Zygomycotes *Rhizopus arrhizus *and *Phycomyces blakesleeanus *have undergone several single gene or whole genome duplications. The Saccharomycetes, Schizosaccharomycetes, and Microsporidia have lost their class-XVII myosin.

**Figure 9:**
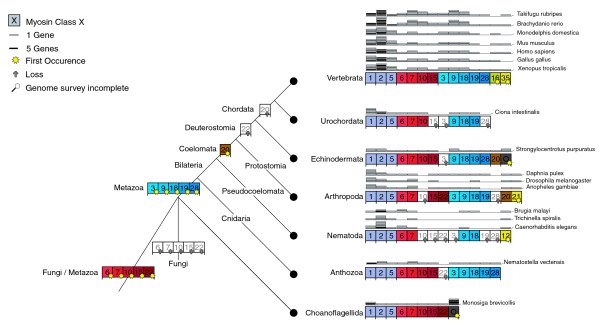
Schematic drawing of the evolution of myosin diversity in the Fungi/Metazoa lineage based on the 'accepted' taxonomy. The inventions and losses of the myosin classes have been plotted onto the 'accepted' phylogeny of the Eukaryotes available at NCBI. Branch lengths do not correspond to any scale.

Two different models can be proposed for the further evolution of the Metazoa (Figures 8 and 9). In both models a considerable boost of myosin diversity happened at the early evolution of Metazoa. The most reasonable model based on the myosin class distribution suggests an increase of the myosin diversity in three steps. After separation of the Fungi, the Metazoa developed four new classes, class-VI, class-VII, class-IX, and class-XVIII. These classes are shared by species of all Metazoa taxa sequenced so far, except the choanoflagellate *Monosiga brevicollis*, which does not contain class-IX and class-XVIII myosins. However, single species of the other taxa have also lost their members of these four classes; for example, the nematode *Trichinella spiralis *contains only a class-VII myosin, the *Caenorhabditis *species have lost their class-XVIII myosins, and the *Drosophila *species have lost their class-IX myosin. Our model places the choanoflagellates to the Coelomata that invented the related class-X, class-XV, and class-XXII myosins. After separation of the choanoflagellates, the Bilateria gained another three classes, class-III, class-XIX, and class-XX. The Deuterostomia, to which we placed the Cnidaria, invented the class-XXVIII myosins and lost class-XXII myosins. Later in evolution, the Chordata lost class-XX myosins. This model proposes the continuous invention of new myosin classes over a relatively long time and the subsequent loss of single myosin classes by certain species and lineages. The placement of the Cnidaria to the Deuterostomia is surprising as the Cnidaria are commonly considered to be a sister group of the Bilateria. However, the analysis of the *Nematostella vectensis *genome showed that, from a genomic perspective, *Nematostella *more closely resembles modern vertebrates than the fruit fly or nematodes [29], which is consistent with our analysis. But as long as genome sequences of further Cnidaria species are not available, this placement could also be the result of long branch attraction effects in the phylogenetic tree. Sequencing of further species of the lineages Choanoflagellida, Cnidaria, and Echinodermata, which are as yet represented only by single species, will provide a better picture of these taxa, as has been obtained for the nematodes, Arthropoda, and vertebrates, which show a wide distribution of the myosin content between their member species. For example, during the evolution of the Arthropoda, the Insecta lost the class-XIX myosin. Later in evolution the ancestor of all *Drosophila *species lost the class-III and class-IX myosins, and finally most *Drosophila *species lost the class-XXII myosin. Most of the lineages like the Nematoda, Arthropoda and Vertebrata have developed further branch-specific myosins. We propose that sequencing of related organisms to *Strongylocentrotus purpuratus *and *Monosiga brevicollis *will result in the classification of their orphan myosins and, thus, also of branch-specific myosins for these lineages.

In contrast, the metazoan tree based on classical taxa and nodes shows the invention of ten myosin classes in a very short time scale (Figure 9). The evolution of the Metazoa would thus mainly be characterized by gene losses. While the Anthozoa *Nematostella vectensis *shares all its 12 myosin classes with vertebrates, the nematodes must have lost 6 of the 13 common Metazoa myosin classes. The nematode *Trichinella spiralis *has lost another three of the remaining classes, sharing only four classes with the other Metazoa. The Arthropoda must also have lost at least two of the common Metazoa myosin classes. This scenario, the invention of ten myosin classes during the evolution of only two taxa nodes and the subsequent major losses of myosin classes until the final speciation, seems very unlikely compared to the other model that proposes the invention of new myosin classes over a long period with the subsequent loss of single classes.

In both models, the tree of myosin diversity gives clear support for the classical Coelomata hypothesis that groups Arthropoda with Deuterostomia in a monophyletic class. The Nematoda sequenced so far lack four classes that the Arthropoda share with the vertebrates. It is very unlikely that the Nematoda have lost just these four classes and not one or more of the others. The class specific phylogenetic trees show that the Nematoda myosins always separate before the Arthropoda-Deuterostomia split, except for the class-IX myosins, where the Nematoda and Arthropoda homologs group separately from the Deuterostomia homologs. These findings illustrate the advantage of analyzing the diversity of a large protein family in contrast to looking at single-gene phylogenies that have supported the monophyletic grouping of Nematoda and Arthropoda in some cases [30].

**Figure 10:**
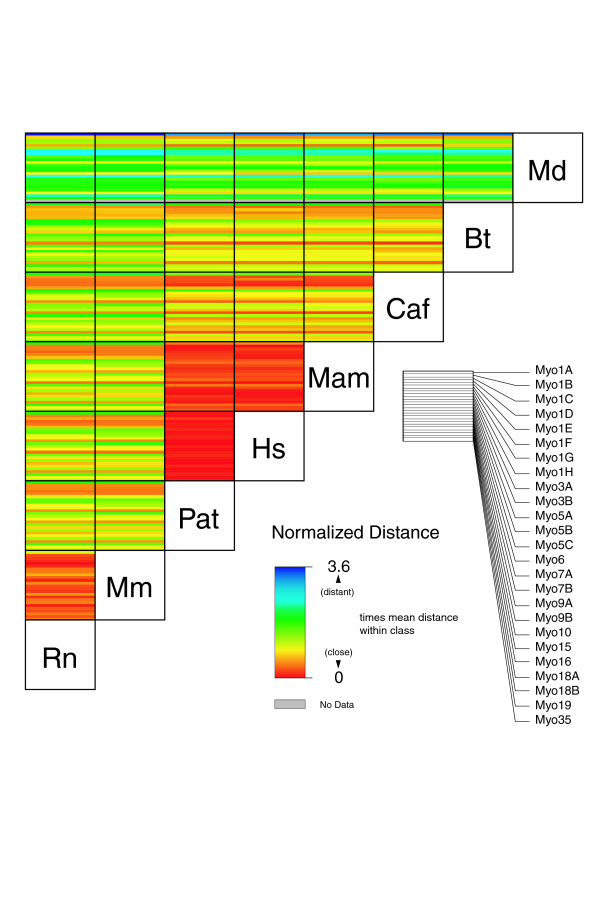
Asynchronous evolution of mammalian myosin proteins. The matrix illustrates the normalized distances between corresponding sequences. Asynchronous evolution is observed if the pattern of the deviation from the mean is different. For example, the pattern from rat to the other mammalian species is very similar, illustrating their synchronous evolution in general. However, there are differences in the patterns of some class-I myosins between rat and mouse and opossum, indicating their asynchronous evolution. In contrast, the sequence comparison patterns of cow and the other mammals are very different, indicating the asynchronous evolution of all cow myosin genes. The abbreviations for the organisms are: Rn, *Rattus norvegicus*; Mm, *Mus musculus*; Pat, *Pan troglodytes*; Hs, *Homo sapiens*; Mam, *Macaca mulatto*; Caf, *Canis familiaris*; Bt, *Bos Taurus*; Md, *Monodelphis domestica*.

**Figure 11:**
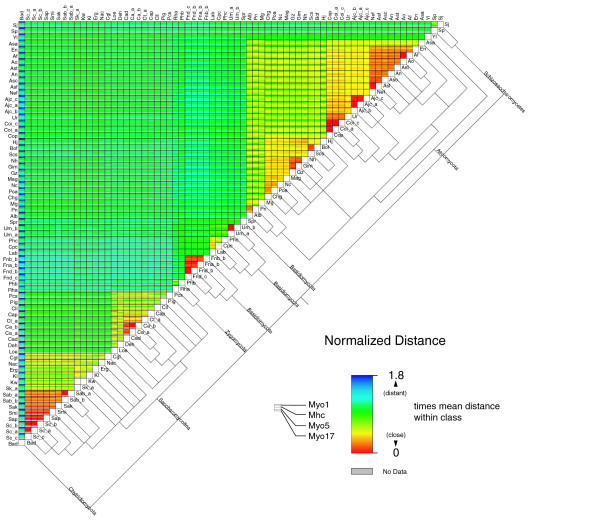
Asynchronous evolution of fungi myosin proteins. The matrix is shown in a similar way as in Figure 10. The consensus tree from the analysis of the single myosin class trees is shown. The obtained polytomic tree is the result of the asynchronous evolution of the different species. The abbreviations for the organisms are: Bad, *Batrachochytrium dendrobatidis JEL423*; Sc_c, *Saccharomyces cerevisiae S288c*; Sc_a, *Saccharomyces cerevisiae YJM789*; Sc_b, *Saccharomyces cerevisiae RM11-1a*; Sap, *Saccharomyces paradoxus NRRL Y-17217*; Smi, *Saccharomyces mikatae IFO 1815*; Sak, *Saccharomyces kudriavzevii IFO 1802*; Sab_b, *Saccharomyces bayanus MCYC 623*; Sab_a, *Saccharomyces bayanus 623-6C*; Sk_a, *Saccharomyces kluyveri NRRL Y-12651*; Kw, *Kluyveromyces waltii NCYC 2644*; Kl, *Kluyveromyces lactis NRRL Y-1140*; Erg, *Eremothecium gossypii ATCC 10895*; Nac, *Naumovia castellii NRRL Y-12630*; Cgl, *Candida glabrata CBS138*; Loe, *Lodderomyces elongisporus NRLL YB-4239*; Deh, *Debaryomyces hansenii CBS767*; Cad, *Candida dubliniensis CD36*; Ca_a, *Candida albicans SC5314*; Ca_b, *Candida albicans WO-1*; Ct_a, *Candida tropicalis MYA-3404*; Cap, *Candida parapsilosis*; Cll, *Clavispora lusitaniae ATCC 42720*; Pig, *Pichia guilliermondii ATCC 6260*; Pcs, *Pichia stipitis CBS 6054*; Rha, *Rhizopus arrhizus RA 99-880*; Phb, *Phycomyces blakesleeanus*; Fnd_c, *Filobasidiella neoformans *var. *neoformans JEC21*; Fnd_b, *Filobasidiella neoformans *var. *neoformans B-3501A*; Fna_b, *Filobasidiella neoformans *var. *neoformans H99*; Fnb_b, *Filobasidiella neoformans *var. *bacillispora R265*; Lab, *Laccaria bicolor S238N*; Cpc, *Coprinopsis cinerea okayama7#130*; Phc, *Phanerochaete chrysosporium RP-78*; Um_a, *Ustilago maydis 521*; Um_b, *Ustilago maydis FB1*; Spr, *Sporobolomyces roseus IAM 13481*; Alb, *Alternaria brassicicola ATCC 96836*; Pn, *Phaeosphaeria nodorum SN15*; Mg, *Mycosphaerella graminicola*; Chg, *Chaetomium globosum CBS 148.51*; Poa, *Podospora anserina*; Nc, *Neurospora crassa OR74A*; Mag, *Magnaporthe grisea 70-15*; Gz, *Gibberella zeae PH-1*; Gim, *Gibberella moniliformis 7600*; Nh, *Nectria haematococca MPVI*; Scs, *Sclerotinia sclerotiorum 1980*; Bof, *Botryotinia fuckeliana B05.10*; Hj, *Hypocrea jecorina QM9414*; Cop, *Coccidioides posadasii C735*; Coi_a, *Coccidioides immitis RS*; Coi_c, *Coccidioides immitis RMSCC 2394*; Ur, *Uncinocarpus reesii 1704*; Ajc_b, *Ajellomyces capsulatus NAmII G217B*; Ajc_a, *Ajellomyces capsulatus NAmII G186AR*; Ajc_c, *Ajellomyces capsulatus NAmI WU24*; Nef, *Neosartorya fischeri NRRL 181*; Asf, *Aspergillus fumigatus Af293*; Asc, *Aspergillus clavatus NRRL 1*; An, *Aspergillus niger ATCC 1015*; Ast, *Aspergillus terreus NIH2624*; Ao, *Aspergillus oryzae RIB40*; Af, *Aspergillus flavus NRRL3357*; En, *Emericella nidulans FGSC A4*; Asa, *Ascosphaera apis USDA-ARSEF 7405*; Yl, *Yarrowia lipolytica CLIB99*; Sp, *Schizosaccharomyces pombe 972h*-; Sj, *Schizosaccharomyces japonicus yFS275*.

The comparative analysis of the phylogenetic relationship of the species in single myosin classes showed several incongruities. We hypothesized that the myosin genes of the corresponding organisms might have evolved asynchronously, as has been observed for a number of yeast genes [31]. From the phylogenetic tree we therefore determined the distances between pairs of sequences. To compensate for differences in general diversity within each class, all distances were normalized. Asynchronous evolution is visualized by the comparison of the deviation from the mean distances. As examples we analyzed the myosins of completely sequenced mammalian (Figure 10) and fungal (Figure 11) genomes. As expected, all primates are very closely related, with the chimpanzee generally closer to *Homo sapiens *than to macaca. The myosin proteins from dog and cow are more closely related to those of the primates than to those from rodents. The opossum *Monodelphis domestica *is, in general, the most divergent mammal with respect to myosins, although in the case of Myo1E and Myo16, it is most closely related to the dog and the primates than to the rodents. The myosin proteins from cow show the most asynchronous phylogenetic relationship of the analyzed mammalian genomes. They either diverge before the split of the rodents and primates/dog, after this split, or form a monophyletic class with the corresponding dog orthologs. Hence, it is not possible either to resolve the phylogenetic grouping of the cow in general, or to do so by using the myosin proteins, or sequences from additional mammals have to be added to better resolve the tree.

The fungal myosins show several distinct groups that are related to the established taxa. However, the analysis resolves some so far unrecognized relationships. The Saccharomycotina do not group to the Ascomycota in all myosin classes, but have evolved asynchronously. Based on our analysis of the myosins, the Saccharomycotina should be considered as an independent clade that evolved from Fungi, in parallel with the Ascomycota, the Basidiomycota, the Zygomyocota, and the Schizosaccharomycetes. These clades developed very asynchronously so that their phylogeny cannot be resolved. In addition, the species in these clades have undergone considerable asynchronous development. *Yarrowia lipolytica*, which has been considered a yeast species, is more closely related to the Ascomycota than to the Saccharomycotina, based on both the phylogenetic relation of the respective myosin homologs and its myosin content as it contains a class-XVII myosin that all Saccharomycotina have lost.

**Figure 12:**
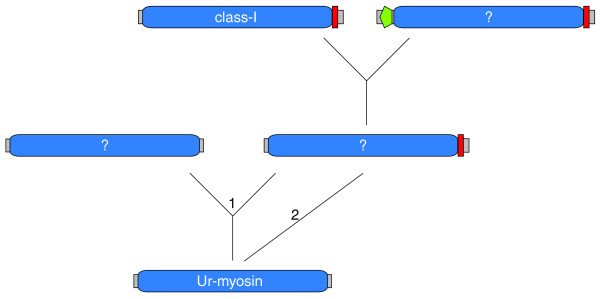
Evolution of the first myosins. The first myosin, called ur-myosin, is expected to consist only of the myosin motor domain. By domain fusion it generated the IQ motif either directly carboxy-terminal to the motor domain (2), or after a gene duplication event (1). After a further gene duplication event, this myosin developed to the class-I myosins as well as the ancestor of most of the other myosin classes after fusion with an SH3 domain (which developed into the amino-terminal SH3-like domain).

What did the very first myosin look like? In the beginning of eukaryotic evolution, the myosin motor domain had been developed (Figure 12). During subsequent early evolution, an extensive process of domain fusions started, during which the carboxy-terminal IQ motif was added first. After duplication of this gene, the amino-terminal SH3-like domain was fused to the motor domain. These two domain organizations are shared by myosins of all species. The class-I myosins show the widest taxonomic distribution, are devoid of the amino-terminal SH3-like domain and, thus, are suggested to be the first distinct myosin class to have evolved. We propose that the most ancient myosin motor domain had a sequence very close to that of the class-I myosins.

## Conclusion

Here, we present the phylogenetic analysis of 2,269 manually annotated myosin proteins. The previously assigned 19 myosin classes were confirmed and 16 new classes with unique domain organizations defined. A phylogenetic tree has been constructed that includes information about the class distribution and evolution in certain taxa as well as the phylogenetic information contained in class-specific subtrees. The analysis shows the Choanoflagellida as part of the Metazoa lineage and the cnidaria (*Nematostella vectensis*) as having diverged after the separation of Deuterostomia and Protostomia. The myosin data show that several taxa have evolved asynchronously, for example the Mammalia and the Fungi.

The presented tree will increase in resolution as more organisms are sequenced. To increase the fine resolution, more sequences of intermediate taxa, for example, in the metazoan lineage, are needed. For some major taxa a significant amount of species has to be sequenced to get the resolution already obtained for the fungi and metazoans. For example, only eight species of the Viridiplantae have completely been sequenced so far. In particular, sequencing of further underrepresented taxa will increase myosin diversity. The myosin data presented here will allow the correct annotation and classification of all upcoming homologs. We hope that CyMoBase [21,32], which stores and presents all related information, will be an invaluable tool in all areas of myosin and motor protein research, and in classical taxonomy.

## Materials and methods

### Identification of myosin family proteins

Myosin genes were identified in iterated TBLASTN searches of the completed genomes of 181 organisms starting with the protein sequence of *Dd*MhcA. All hits were manually analyzed at the genomic DNA level. The correct coding sequences were identified with the help of the multiple sequence alignment of the myosins. As the amount of myosin sequences increased (especially the number of sequences in classes with few representatives), many of the initially predicted sequences were reanalyzed to correctly identify all exon borders. Where possible, EST data have been analyzed to help in the annotation process. Now, all designated myosin classes contain enough members to correctly predict any additional member sequence in the future. However, some of the orphan myosins (for example, from *Tetrahymena thermophila *and *Paramecium tetraurelia*) might still contain wrongly predicted exons in the tail regions, because sufficient comparative genomic data are not yet available. For some organisms only EST data are available to date, and myosin sequences identified in these databases have been included in the analysis as long as the sequences contains at least 100 residues. These short sequences cannot be, and have not been, used in the phylogenetic analysis but are important to define the myosin inventory with as many organisms as possible. In addition to the analysis of these large-scale sequencing projects, all myosin sequences in the nr database at NCBI have been collected and reanalyzed. Many of these sequences contain sequencing errors, mispredicted exons, and wrongly predicted gene borders.

Some of the genes contain alternative splice forms for the motor domain. The different splice forms were not considered independently in the analysis but in all cases the same splice forms were taken for homologous myosins. All sequence-related data (names, corresponding species, GenBank IDs, alternative names, corresponding publications, domain predictions, and sequences) and references to genome sequencing centers are available through CyMoBase [21,32]. The annotated sequences and the alignment of the motor domain sequences are available as Additional data files 5 and 6, respectively.

### Building trees

The phylogenetic tree was built based on a manually constructed and maintained structure-guided multiple sequence alignment. The phylogenetic tree is unrooted and was generated using neighbor joining and the bootstrap (1,000 replicates) method as implemented in ClustalW (standard settings) [33]. The phylogenetic tree presented in Additional data file 2 was visualized using TreeDyn [34]. The schematic tree was constructed using the following criteria. The myosins are separated into 35 classes. Thus, we assigned a class inventory to every organism. The myosin classes are not only well-separated based on their motor domains but also due to the unique composition of their tail domains. Thus, we conclude that species having myosins of the same class must have had a common ancestor. It is extremely if not completely unlikely that myosins with these distinct features were invented independently. The other criterion is the analysis of the different trees of the myosin classes. Looking at the tree of a single class, for example, the class VI myosins, it is obvious that certain taxa always separated earlier than others, for example, the Arthropoda myosins always separated before the mammalian myosins. In the next step we ordered all species according to their class inventory, minimizing the number of myosin class inventions and losses. If taxa have the same class inventory we used the data from the single class trees to resolve their phylogenetic relationship. If the phylogenetic relationships of taxa in single classes contradicted each other, we hypothesized asynchronous evolution and did not resolve the relationship between these taxa.

### Distance maps

The distances between two sequences of one class/variant were obtained from the distance matrix produced by ClustalW using default substitution matrix BLOSUM62 [35]. We collected the distances between all sequences of each class/variant. The distances within each class/variant were normalized by dividing by the mean distance of the class/variant. This set the mean distance of each class/variant to 1. In the distance maps the normalized distances were visualized in blocks, each block representing the distances of all sequences from two species. Asynchronous phylogenetic relationships are visible as color fluctuations within a block.

### Domain and motif predictions

Protein domains were predicted using the SMART [36,37] and Pfam [38,39] web server. The prediction of coiled-coils is based on the coils program. The IQ motifs and amino-terminal domains were predicted manually based on the homology to similar domains of the other myosins. The recognition motifs included in the SMART and Pfam databases are too restrictive, as the motifs have been created based on the small datasets available some years ago. The domain profiles of the other domains have not been revised yet.

## Abbreviations

EST, expressed sequence tag; WGS, whole genome sequencing.

## Authors' contributions

FO performed data analysis. MK designed the study, assembled and annotated all sequences, and performed data analysis. Both authors wrote and approved the manuscript.

## Additional data files

The following additional data are available with the online version of this paper. Additional data file 1 outlines the problems and pitfalls of automatic gene annotation, gene collection, domain prediction, and sequence alignment. Additional data file 2 shows the complete phylogenetic tree of 1,984 myosins. Additional data file 3 is a table of the complete myosin inventory of all 328 species. Additional data file 4 provides references for sequence data, listing of all organisms and related publications with references and links to genome sequencing centers. Additional data file 5 lists the myosin sequences. These sequences are the basis for the myosin inventory and the domain prediction figures. Additional data file 6 shows the alignment of the myosin head domains used to compute the phylogenetic tree shown in Additional data file 2.

## Supplementary Material

Additional data file 1 Problems and pitfalls of automatic gene annotation, gene collection, domain prediction, and sequence alignment. Click here for file

Additional data file 2 Complete phylogenetic tree of 1,984 myosins. Click here for file

Additional data file 3 Complete myosin inventory of all 328 species. Click here for file

Additional data file 4 All organisms and related publications with references and links to genome sequencing centers. Click here for file

Additional data file 5 All annotated myosin sequences in fasta format Click here for file

Additional data file 6 Alignment of the myosin head domains used to compute the phylogenetic tree shown in Additional data file 2 Click here for file
