# Supplementary material for: Drawing the tree of eukaryotic life based on the analysis of 2,269 manually annotated myosins from 328 species
Source: Genome Biol. 2007 Sep 18;8(9):R196. doi: 10.1186/gb-2007-8-9-r196 (PMC2375034; doi:10.1186/gb-2007-8-9-r196)
Supplement: Additional data file 1 — Problems and pitfalls of automatic gene annotation, gene collection, domain prediction, and sequence alignment. [file gb-2007-8-9-r196-S1.pdf]

Problems and pitfalls of automatic gene annotation, gene collection, domain prediction, and sequence alignment

Because of the following problems within the automatic gene annotation process it is absolutely necessary to manually check and annotate all genes. Almost every myosin gene prediction and its translation produced by the automatic processes contains errors derived from including intronic sequence and leaving out exons, as well as wrong predictions of start and termination sites. It is also absolutely necessary to reanalyse previously published data, as these also contain many sequencing errors (especially sequences produced in the last century) and wrongly predicted translations. Wrongly predicted genes are the main reason for wrong results in domain predictions, multiple sequence alignments and phylogenetic analyses. In the following sections we show and discuss examples to these problems.

1. Automatic gene annotation and gene collection

A. Data obtained from whole genome sequencing (WGS) and EST sequencing centers

One of the major obstacles in performing genome analysis projects is that different databases (at different sequencing centers) often contain different data! Not only that the databases just contain older or newer versions of the genome assembly, but in some cases they contain just different data. Therefore one can find genes in a database that cannot be found in others although both databases claim to provide access to the "whole genome assembly".

Problems with cDNA and EST data:

Even in the cases where it is claimed, not all cDNA clones contain the full-length sequence. EST data does also not contain all proteins and, more often than one would expect, genomic clones are obtained. Of course, EST data also contain many sequencing errors leading either to frame shifts or different amino acids. As the EST data is also used in the automatic annotation processes, these problems lead to wrongly predicted genes.

Problems with genome sequencing data:

Especially the low coverage WGS projects (e.g. the mammalian genomes sequenced with 2x coverage) accumulate many sequencing errors leading to frame shifts and mutations. The low coverage WGS sequencing also results in very short contigs, and especially the genes of species, that contain many and long introns (e.g. mammalia), are spread over dozens of

contigs. If large protein superfamilies like the myosins are analysed that contain closely related homologs (e.g. the many class II myosins) it is absolutely impossible to merge the contigs without risking to mix homologs together.

WGS is often not complete (even that of the small yeast genomes) and therefore contains many gaps. WGS data is sometimes incorrectly assembled leading to genes spread over different contigs although we know from very closely related species that these parts belong together.

Problems with exon/intron prediction:

Gene prediction is in almost all cases performed with GENSCAN and/or similar programs. For gene annotation, genome sequencing centers use GENSCAN with trained profiles for each genome. If EST data are available, these data are also used in the gene annotation process. EST data sometimes cover a large part of the ORF's, but are far from representing all genes. Automatic prediction of genes suffers from the following problems:

- Programs are not able to predict GC-AT introns (or AT-AC introns, GG-AG introns, and GA-AG introns).
- Often, there are several very close possibilities for splice site prediction.
- Exons with same splice sites as preceding or following exons are overlooked.
- Small exons of less than 20 bp are not recognised.

One example for a small exon overlooked by the automatic gene prediction programs is shown in Figure 1. All myosins contain a highly conserved motif "LLEKSR". Part of this motif belongs to a small exon with split codons "XLLEK" in e.g. class-VIII, class-X, class-XV, and class-XXI myosins that is not recognised by automatic gene prediction programs.

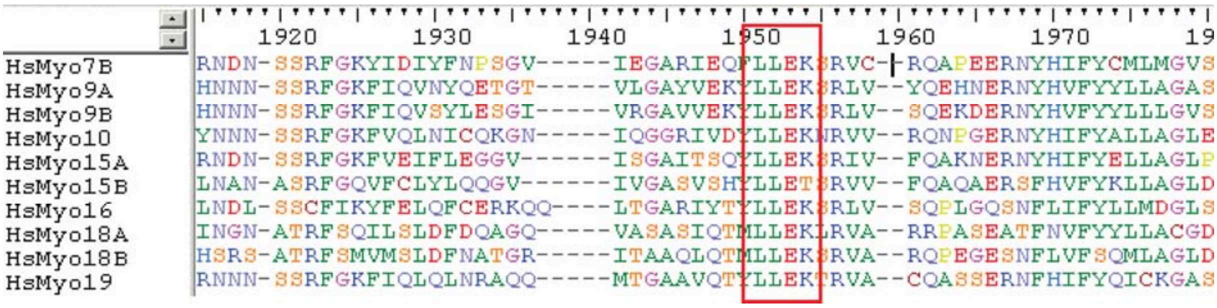

Figure 1: Sequence alignment showing the conserved LLEKSR motif.

Problems with the prediction of gene borders:

EST data negatively biases gene annotation in the first instance. Genome sequencing centers use EST data for the prediction of the genes, if possible, and only in later stages of the annotation process, when data from closely related organisms is available, the original data gets revised.

In principle, small starting exons (e.g. exons consisting of only the starting methionine, as found in a subset of class-I myosins), or starting exons consisting of only a few bases as found in almost all other class-I, the class-V, class-VI, class-VII, and class-X myosins, to name a few, are not recognised at all. In addition, promoters and other small intergenic regions are hardly recognised leading to fused genes. One example is shown in Figure 2. The *Um*Myo1 gene has been submitted to GenBank twice, once in the correct form, and once as fused gene with a helicase. The fused gene is the wrong one, as A) *Ustilago* genes almost never contain introns, B) the introns of the fused genes are between the helicase and the myosin followed by two regions of low complexity, C) all other Fungi (Ascomyotes and Basidiomycotes) have the same gene structure for their class-I myosin, but none has a helicase at its N-terminus, D) the other Basidiomycotes have a homolog of the helicase but the gene is further upstream by dozens or hundreds of kb.

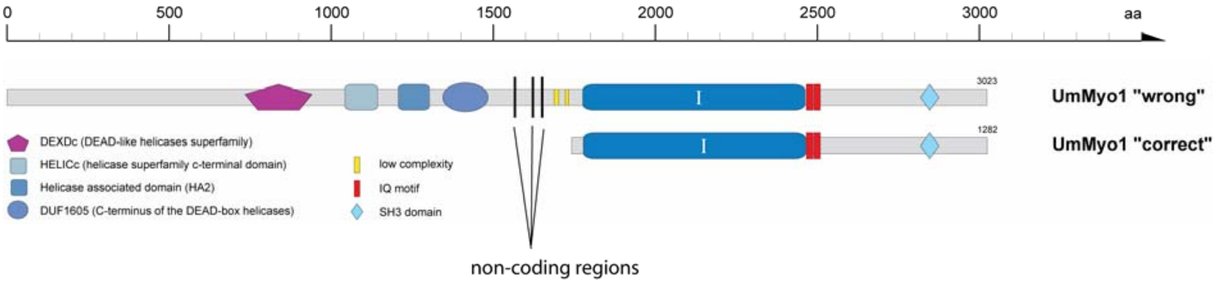

Figure 2: Comparing the wrong and the correct *Ustilago* class-I myosins that have been submitted to GenBank.

Problems with gene identification

Sometimes the intron and exon structures are so uncommon that genes are not identified at all. In most of these cases the genes represent the most divergent homologs. The sensitivity of the BLAST algorithm is also too low to recognize all homologous regions. Thus one has to query all genomes not only with one or two example proteins, but with most if not all of the most divergent homologs. Only with the help of an optimal multiple sequence alignment it is possible to recognize all exons in these divergent homologs.

B. Problems with previously published protein sequences that have been obtained from genomic DNA or cDNA/mRNA

These mispredictions are in most cases very subtle as these errors only become visible in large multiple sequence alignments. Either the methods have not been accurate enough at that time (sequencing methods), or there hasn’t been enough data from homologs to identify the correct exon borders.

Problem: Data has to be reanalysed because of

1. Wrong prediction of coding regions (from genomic DNA).

For example, the *Acanthamoeba castellanii* Myo1B protein has wrongly been predicted at two positions. In Figure 3A four residues are missing in an absolutely conserved motif.

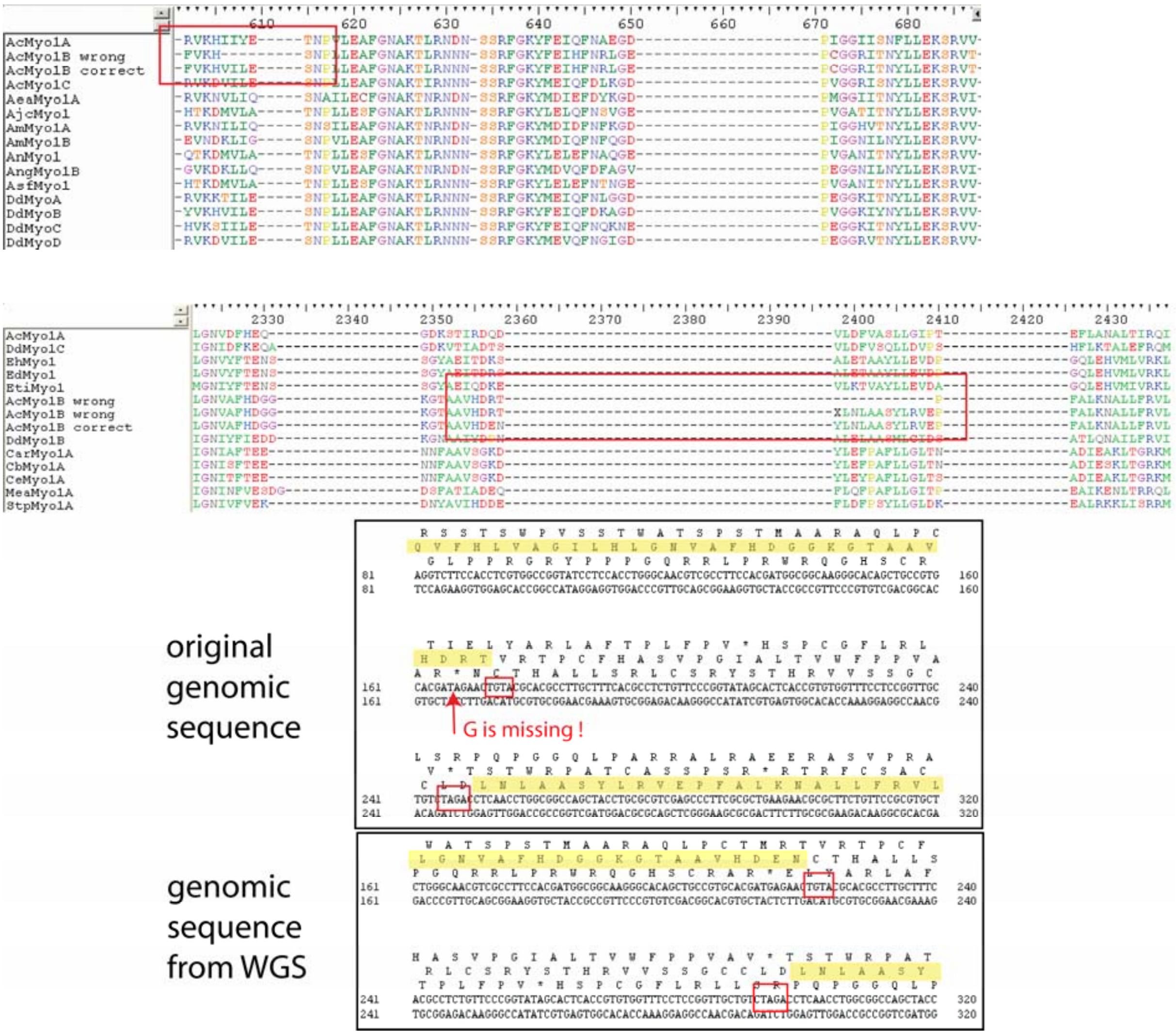

Figure 3: A) Misprediction of a 5’ intron splice site leading to missing four amino acids that are completely conserved in all myosins. B) Comparison of the original (a sequencing error containing) genomic sequence of *Ac*Myo1B with the sequence derived from the whole genome sequencing project.

In Figure 3B, the intron borders at a different position of the same *Myo1B* gene have been mispredicted leading to missing 12 residues. This was due to a sequencing error in the genomic sequence. A reinterpretation of the data suggested that the intron splice sites have been shifted by one basepair on either site. A clone from the whole genome sequencing project finally showed that the intron borders are correct but that a basepair is missing in the 5' exon leading to a frame shift.

2. Bad sequencing results (mRNA/cDNA).

Examples of wrongly sequenced genes are shown in Figure 4. The examples represent the Myo1A (published 1989), the Myo1C (published 1995), and the Myo1E (published 1993) genes from *Dictyostelium discoideum*. Every possible sequencing error has happened: there are additional bases, missing bases, frame shifts in short stretches, or just different bases.

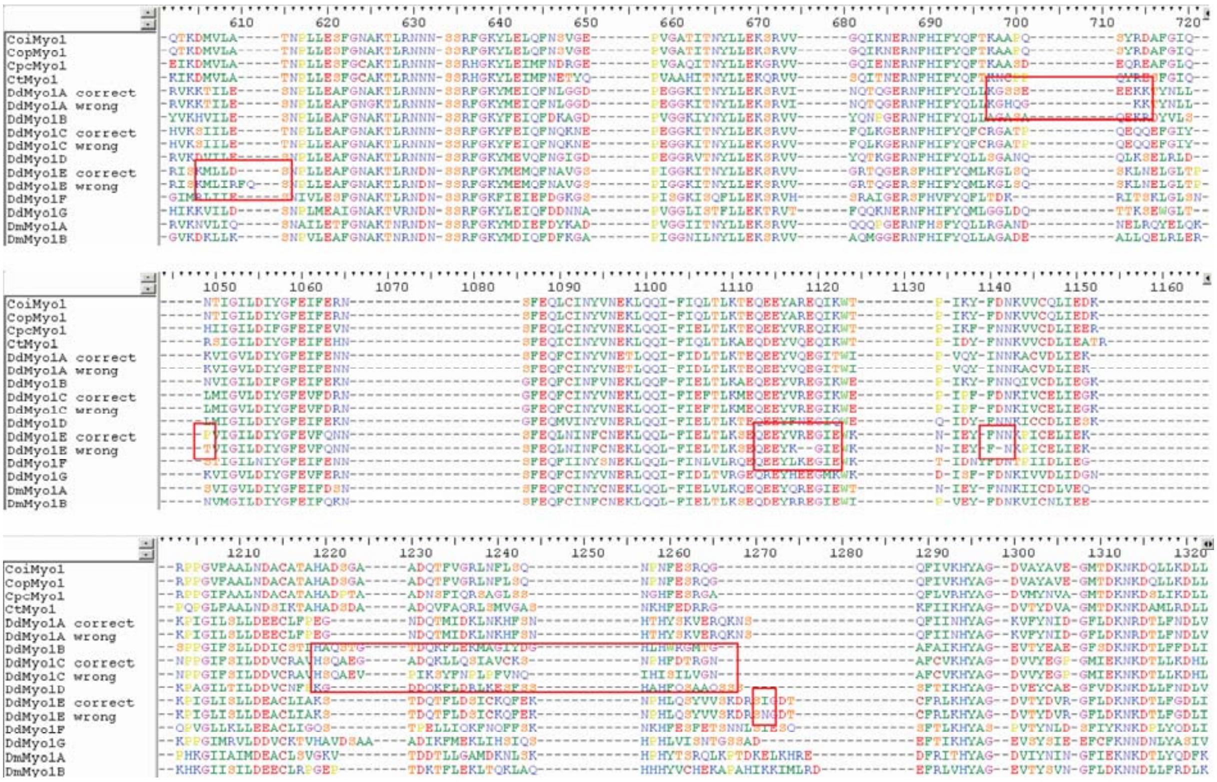

Figure 4: Alignments of class-I myosins demonstrating all kinds of sequencing errors in three *Dictyostelium* sequences.

3. Wrong prediction of N- and C-termini

In many cases, where it has been claimed that the published mRNA represents the full-length transcription of the gene, long stretches are still missing, especially at the termini. Normally, the first methionine is the starting point for the translation. Figure 5 shows an example in

which the complete N-terminus has been missed in the predicted translation although most of it is included in the mRNA. These mistakes in the prediction of the gene termini can easily be overcome by comparing the gene of interest with some close homologs.

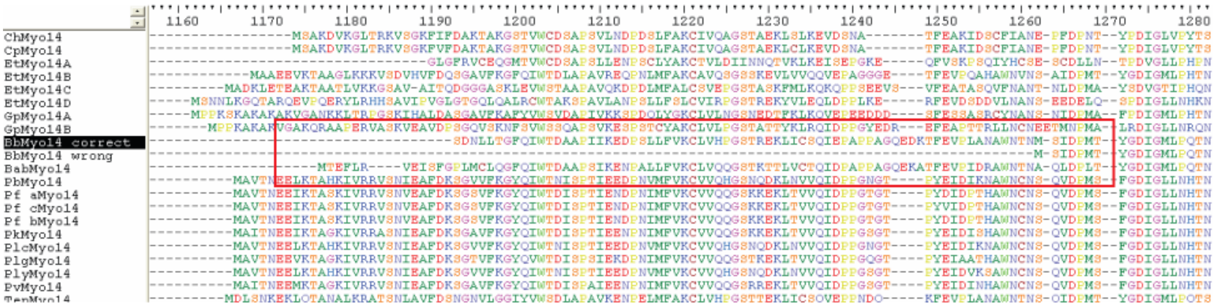

Figure 5: Alignment of the N-termini of the class-XIV myosins. The published predicted sequence of the *Babesia bovis* class-XIV myosin (BbMyo14) is missing its complete N-terminus (about 80 residues), while most of the correct sequence is still encoded by the mRNA. The mRNA is just not representing the full-length gene and thus the starting methionine has not been observed assigning the translational start to the second methionine.

2. Generation of multiple sequence alignments

Multiple sequence alignments are almost always created using ClustalW. All automatic alignment programs rely on the substitution matrices and don't incorporate any knowledge about the 3-dimentional structure. Figure 6 shows the sequence alignment of the myosins at the start of the domain as generated by Pfam. It is obvious that the alignment doesn't follow structural elements. Otherwise it wouldn't show this number of gaps and aligned parts of only 1-2 residues. E.g., the myosin motor domain starts with an alpha-helix of 11 residues. The computer-generated alignment shows six gaps of 1-2 positions in that short motif. If an insertion would really exist, it would mean, that the rest of the alpha-helix would also turn by about 100° thus completely disrupting and changing the interactions of the following residues, that are still conserved according to the alignment. It is absolutely unlikely that an insertion of a residue into the alpha-helix would disrupt the helix at that position while leaving the remaining part intact. In almost all cases, the misaligned part of the computer-generated alignment can be corrected manually removing artificial insertions (Figure 6).

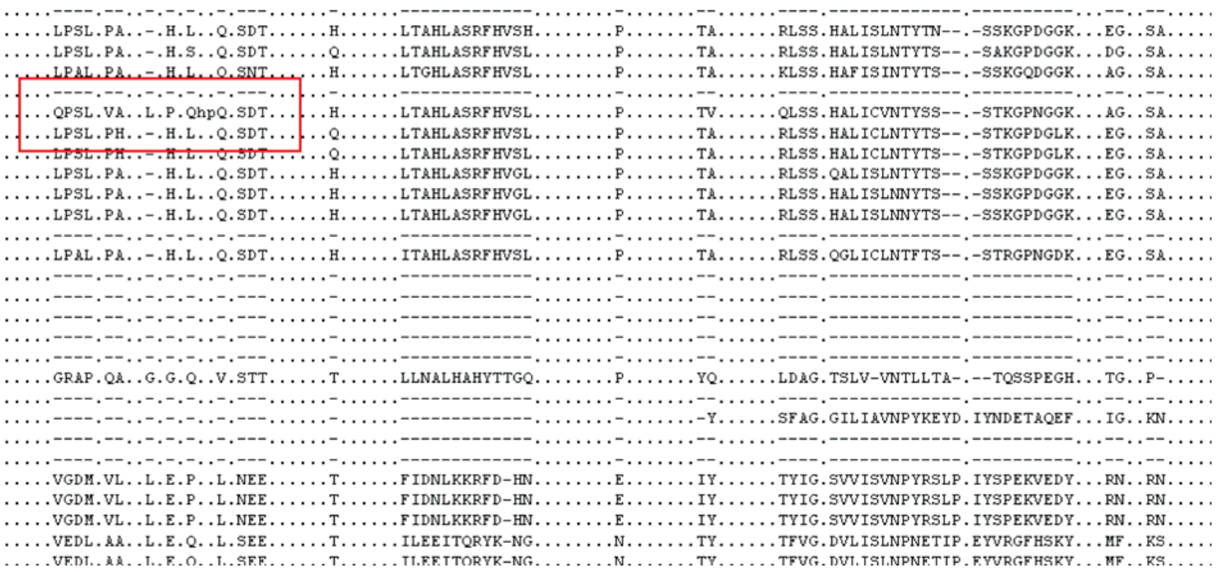

```
...LPAL.PA..-.H.L..Q.SNT......H
...QPSL.VA..L.P.QhpQ.SDT......H   wrongly aligned
QPSLVAL.PQ..-.H.P..Q.SDT......H   correct
...LPSL.PH..-.H.L..Q.SDT......H
```

Figure 6: Computer-generated alignment of the myosin motor domain. It is obvious that the alignment algorithm generated many insertions of single amino acids and many aligned (and thus "conserved") features of just 1-2 residues. This does absolutely not correspond to structural elements of the motor domain but instead introduces insertions of single amino acids into otherwise conserved motifs. The computer-generated misalignments can be resolved by manual inspection and correction (see lower part of the figure).

3. Domain predictions using online databases.

The domain profiles of the domain databases are based on multiple sequence alignments. As outlined above, multiple sequence alignments are heavily dependent on the included sequences, and the manual improvement of the computer-generated alignments. The domain profiles have been determined automatically from the sequences available via generation of a multiple sequence alignment. Thus, they incorporate wrong sequences and duplicates of specific sequences (dependent on how often a sequence has been submitted to GenBank). But, most important, they are only built on a small number of sequences that are most often biased towards certain taxa.

For example, an N-terminal "SH3-like" domain is only predicted for one of the four class 8 myosins of *Arabidopsis thaliana*. The others are either not recognised, or with high e-values.

(E-values for the domain prediction of the N-terminal “SH3-like” domain: AtMyo8A => 1.10e-01 threshold; AtMyo8B => > 1e+03 not recognised; AtMyo8C => 1.40e+00 threshold; AtMyo8D => 2.40e-02 correct prediction). However, the multiple sequence alignment of the corresponding region clearly shows the high similarity of the sequences (Figure 6). Thus, all of them are expected to have an N-terminal “SH3-like” domain.

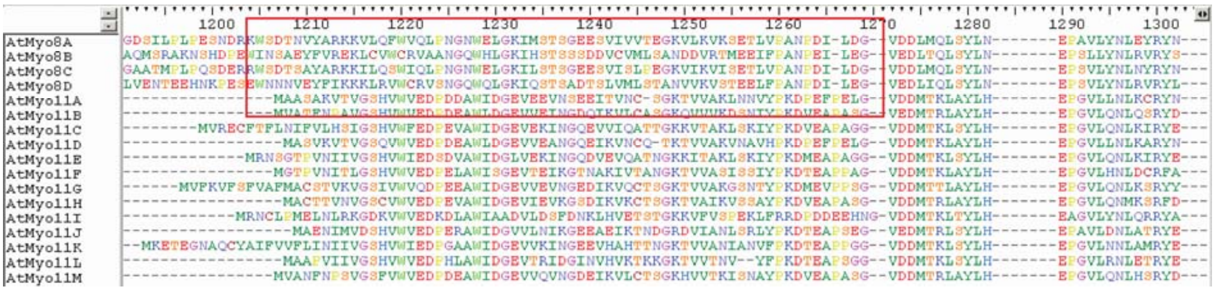

Figure 6: Part of the sequence alignment of *Arabidopsis thaliana* myosins showing the region around the N-teminal “SH3-like” domain.
