# Supplementary figures and images for: Drawing the tree of eukaryotic life based on the analysis of 2,269 manually annotated myosins from 328 species
Source: Genome Biol. 2007 Sep 18;8(9):R196. doi: 10.1186/gb-2007-8-9-r196 (PMC2375034; doi:10.1186/gb-2007-8-9-r196)

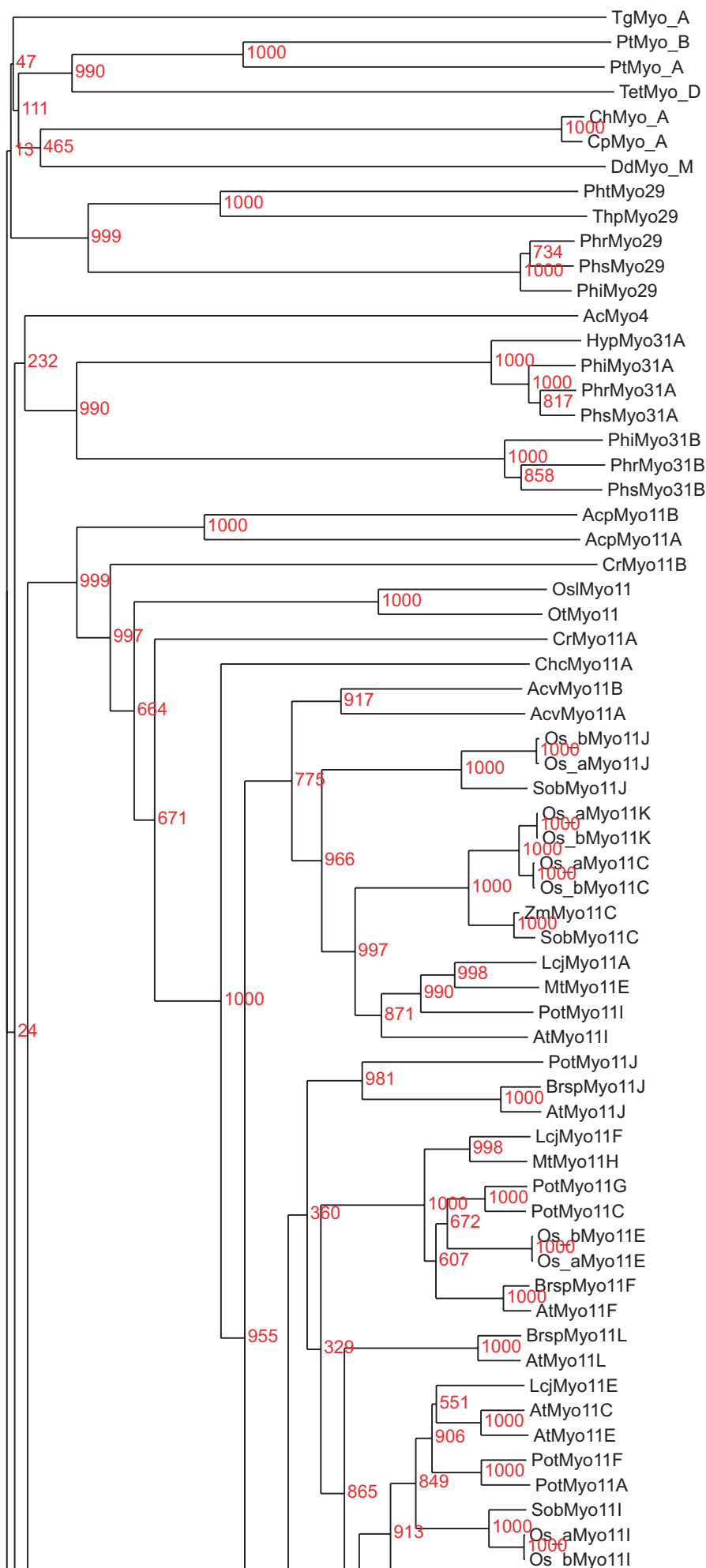

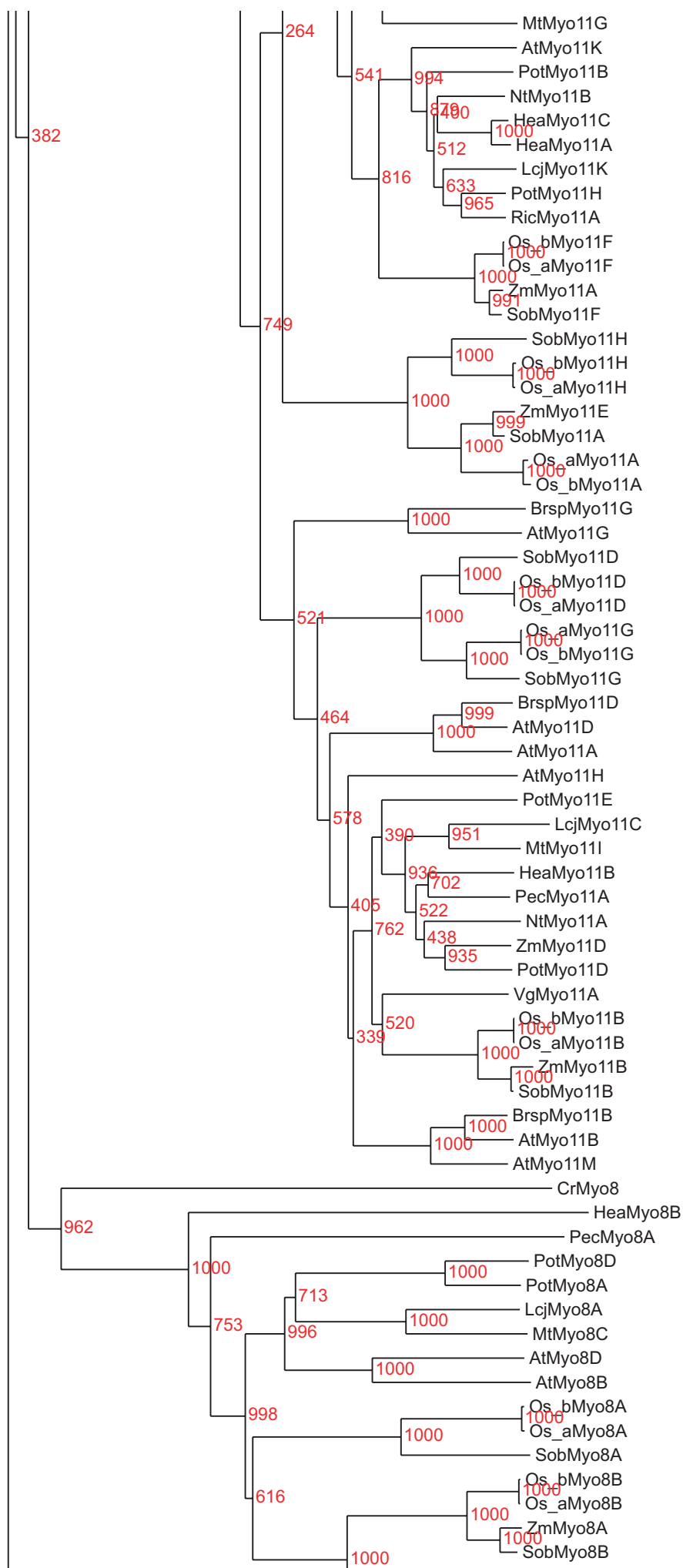

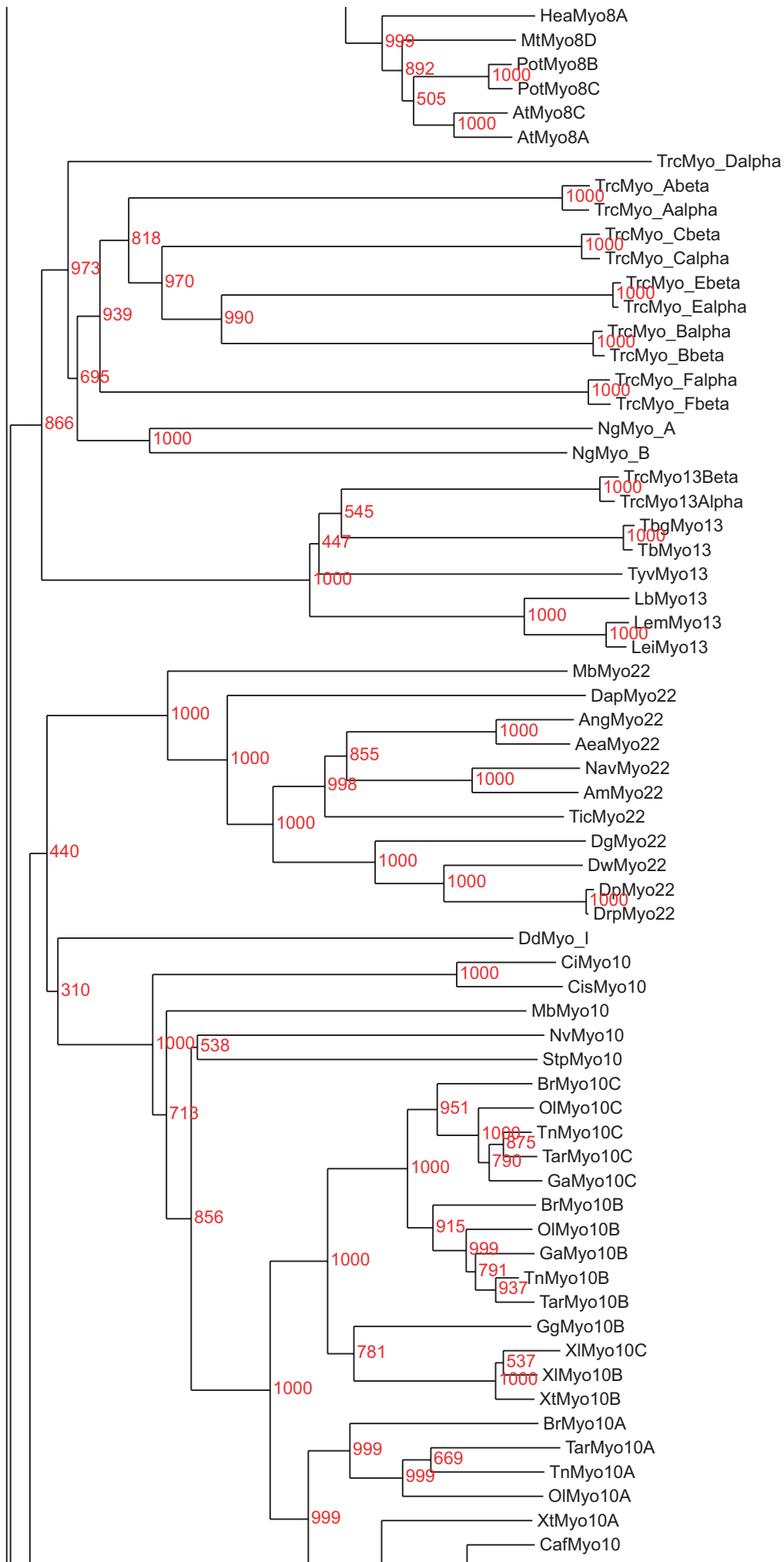

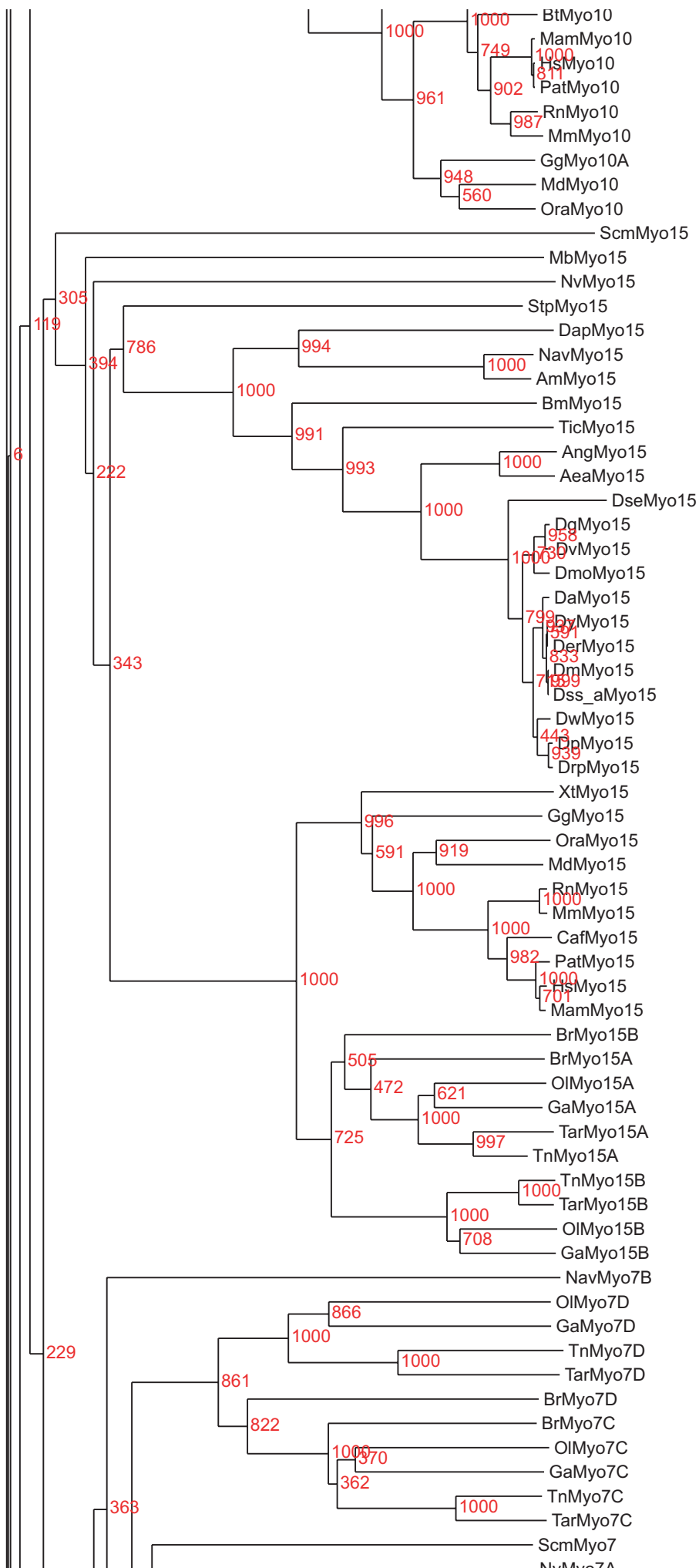

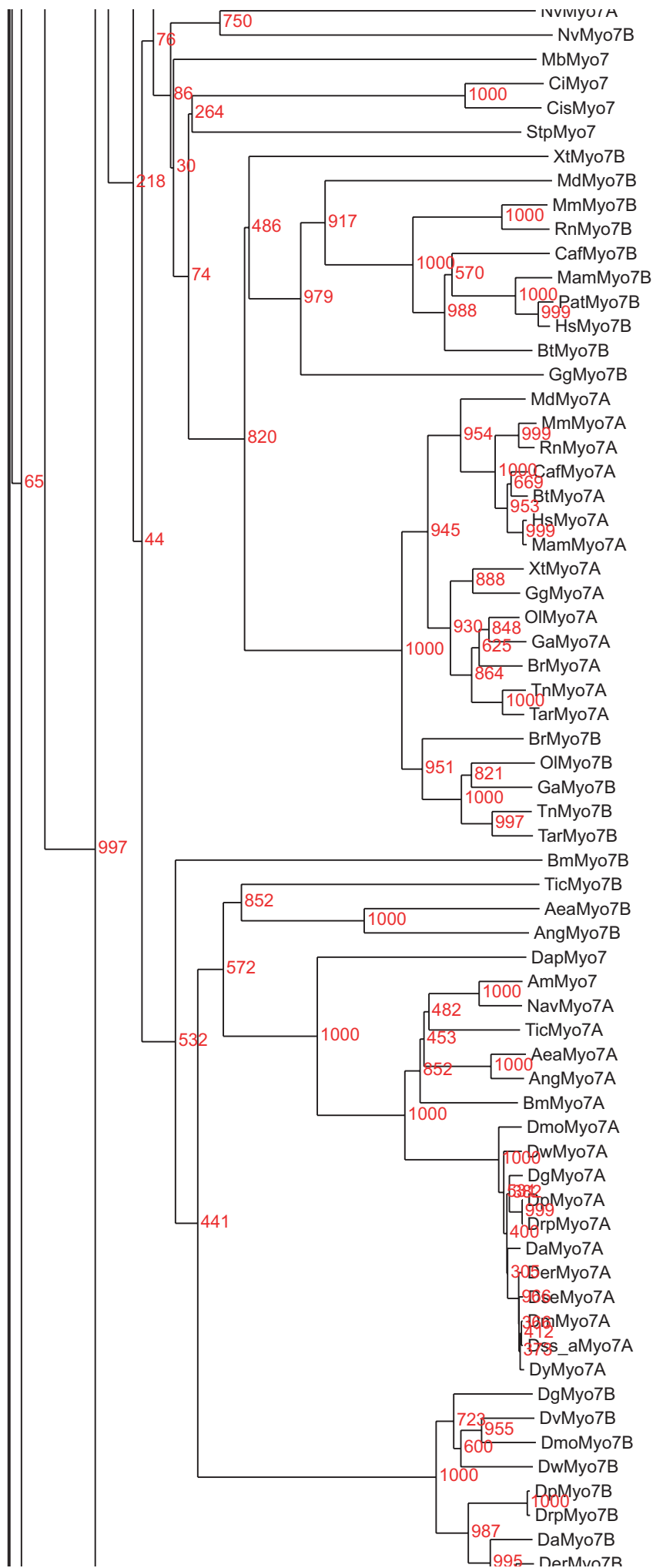

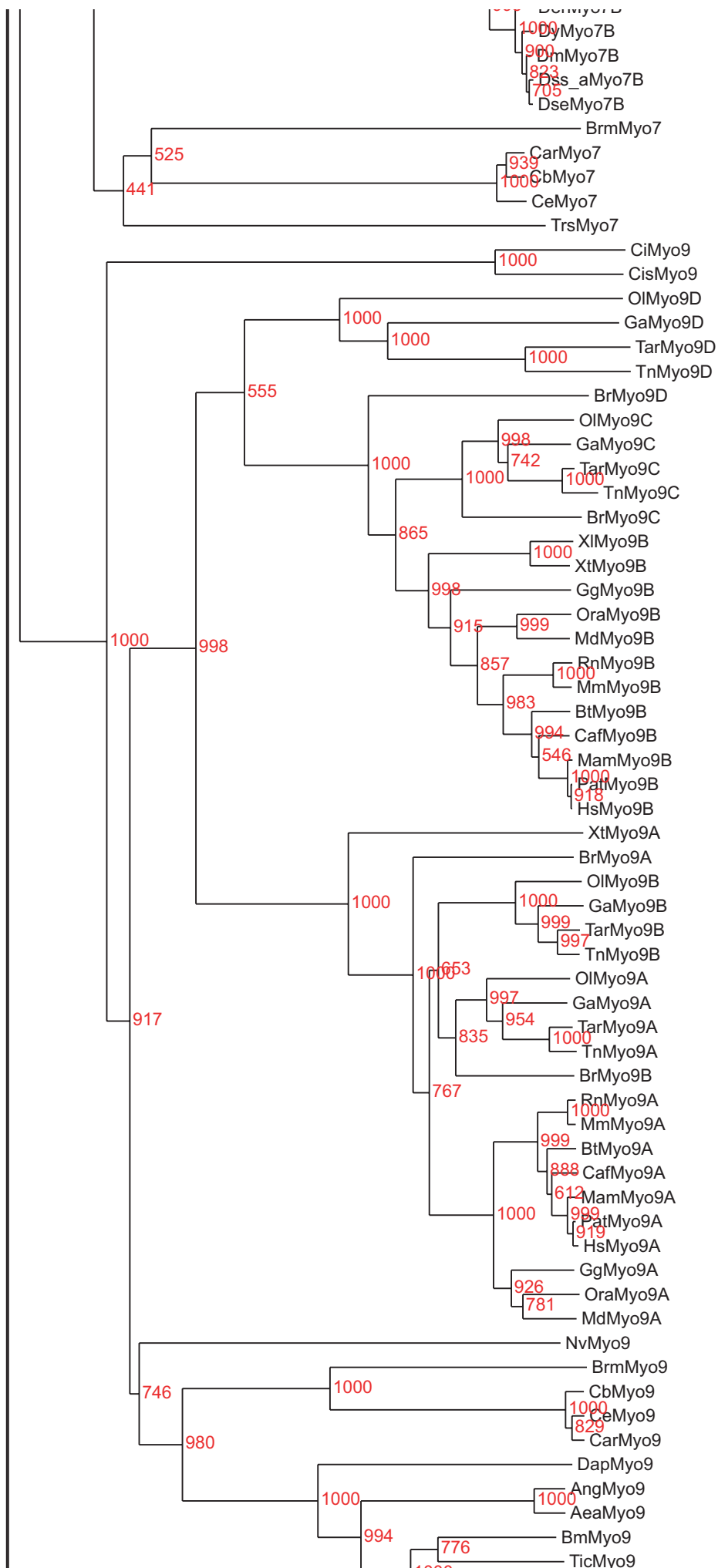

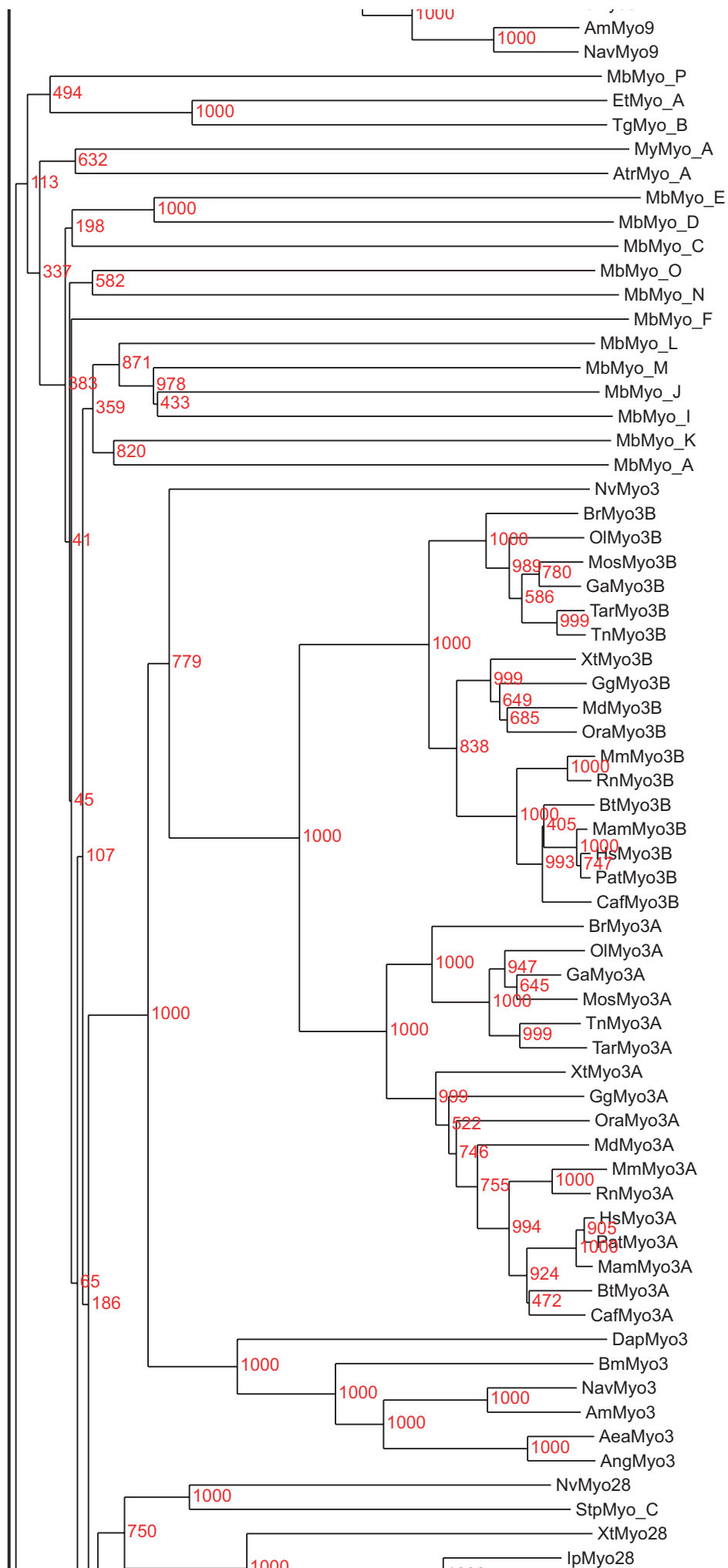

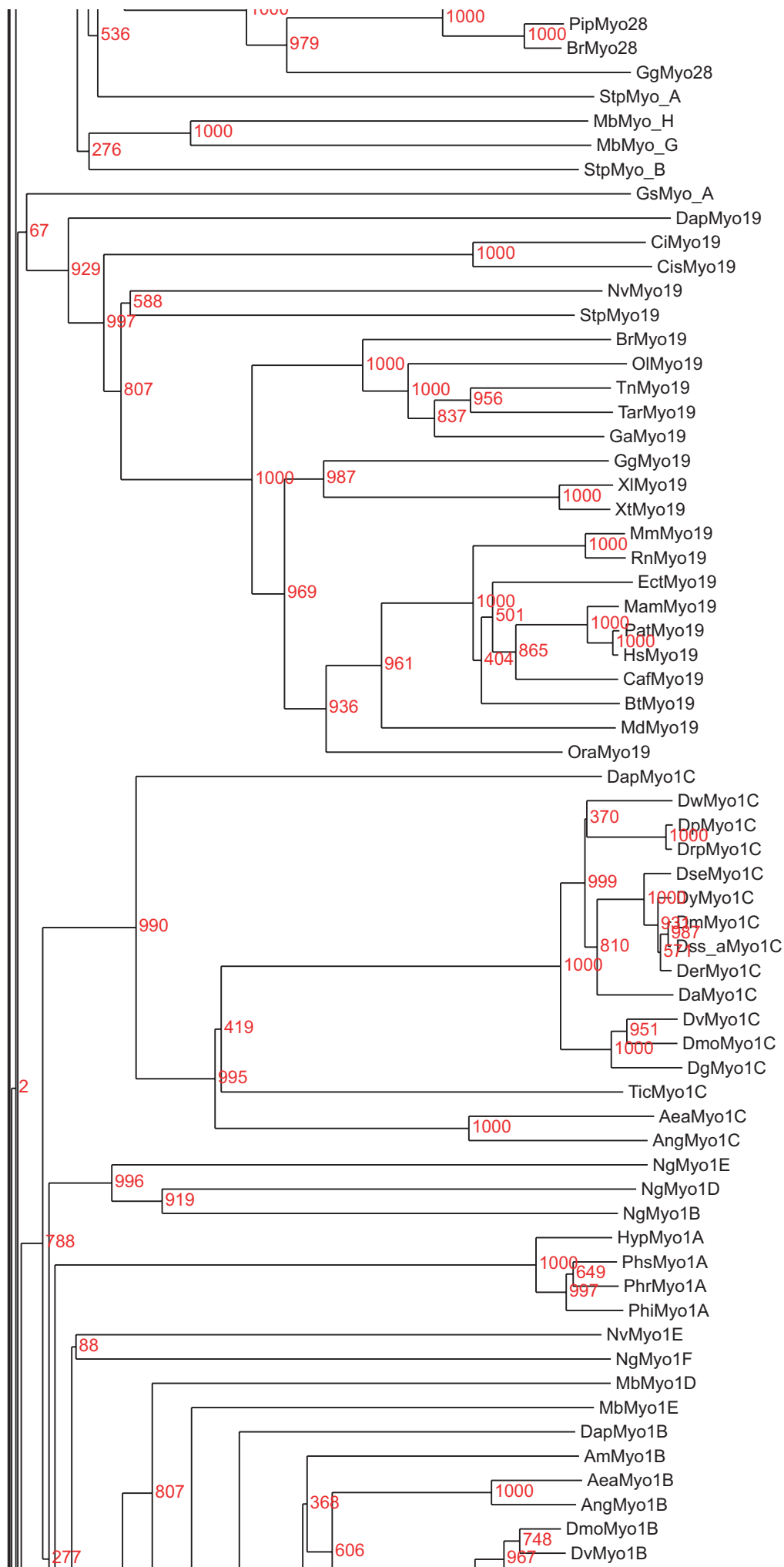

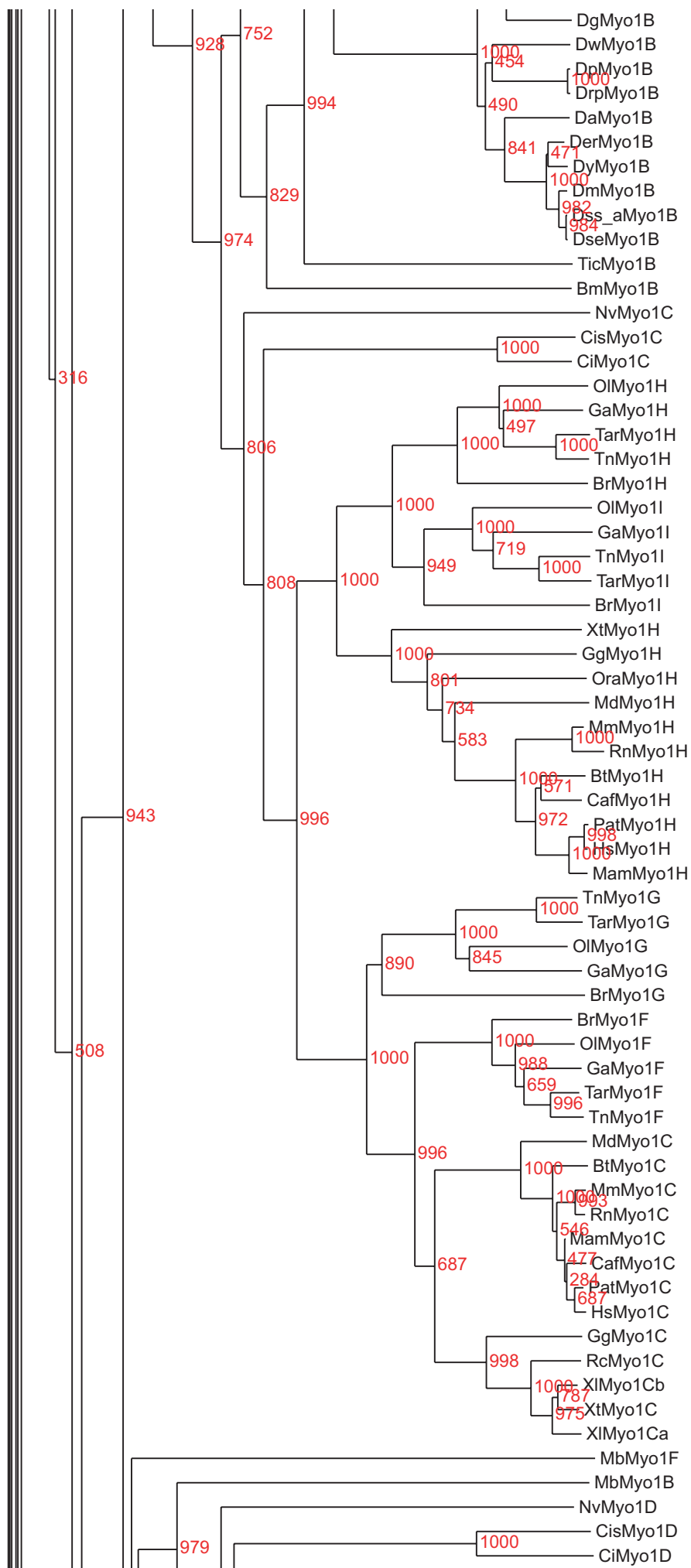

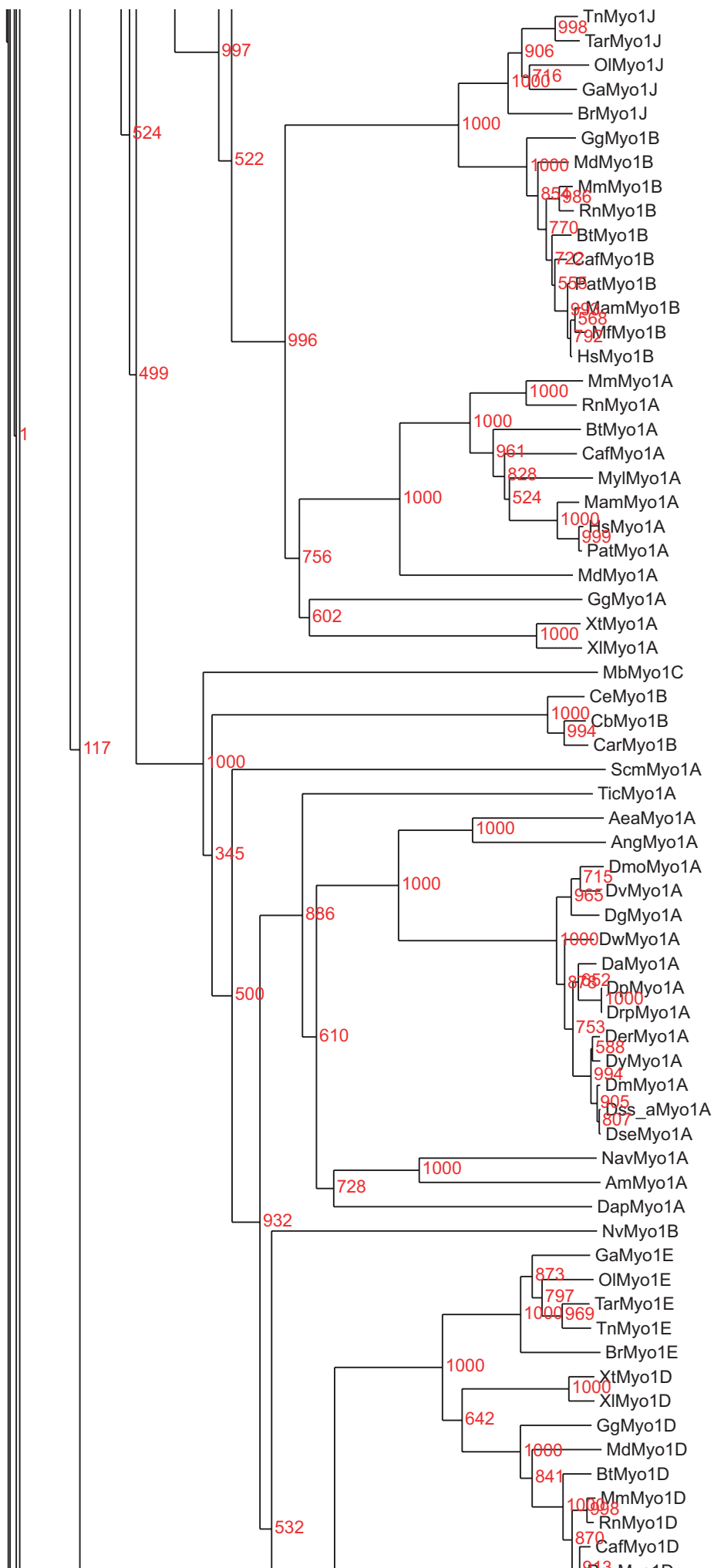

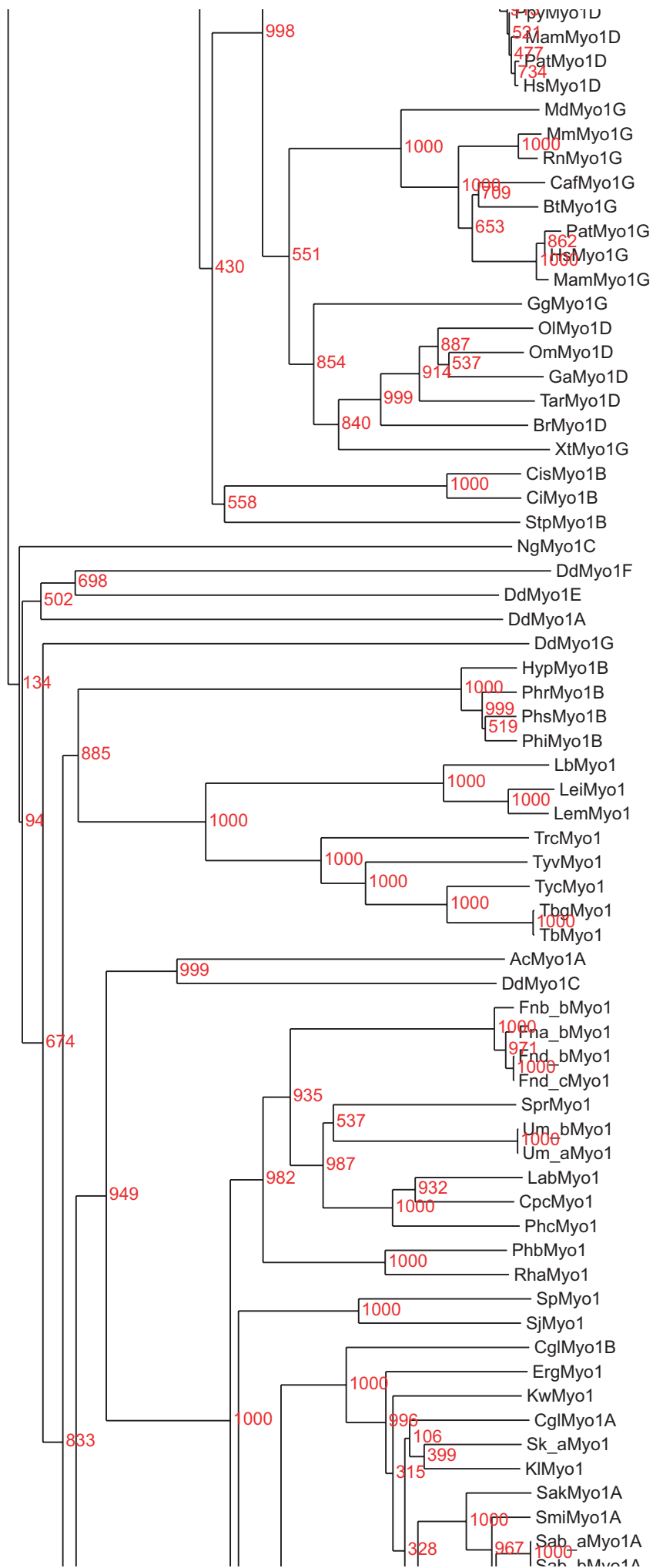

2

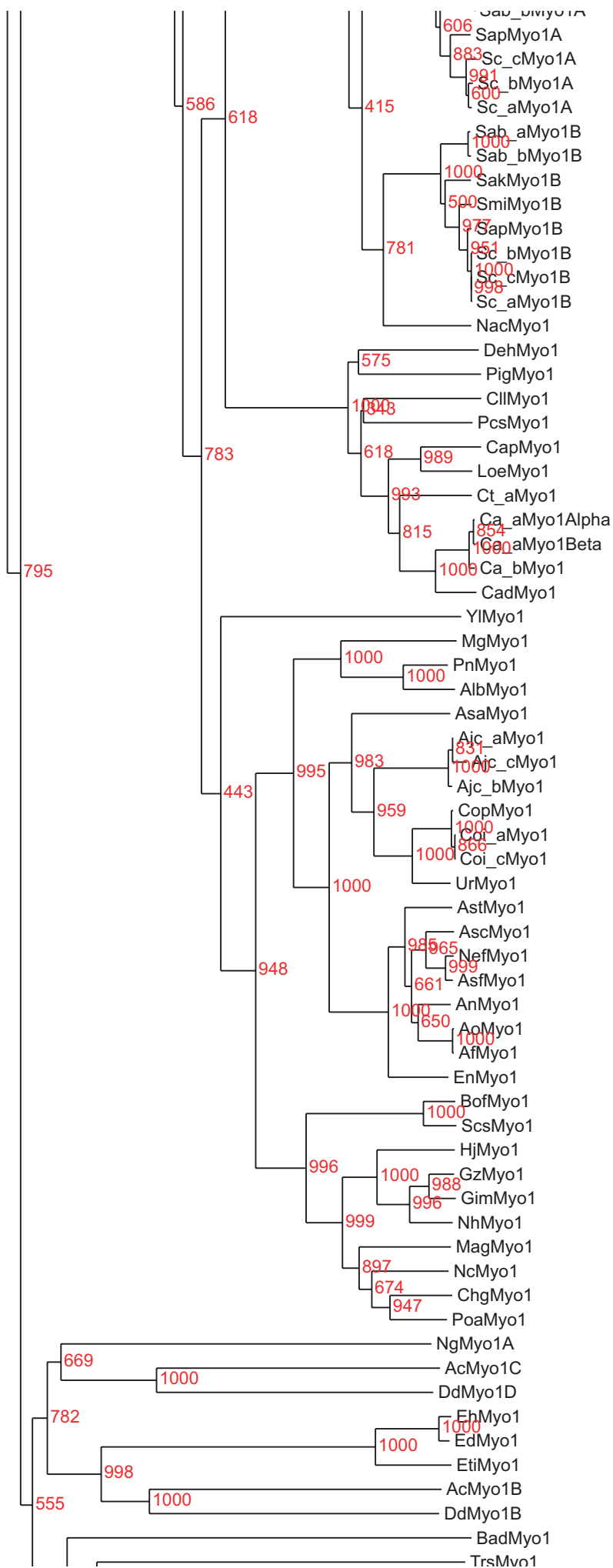

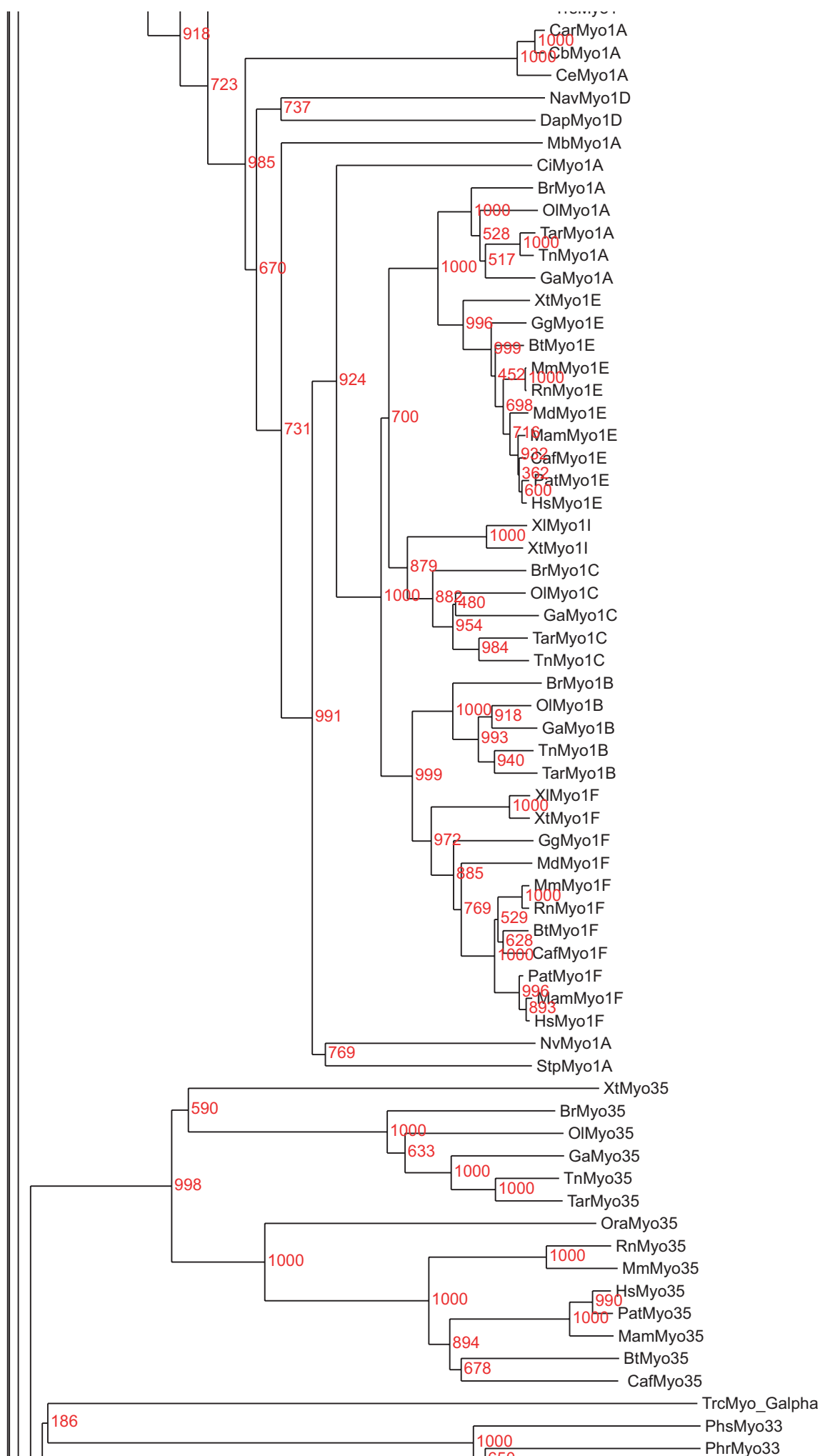

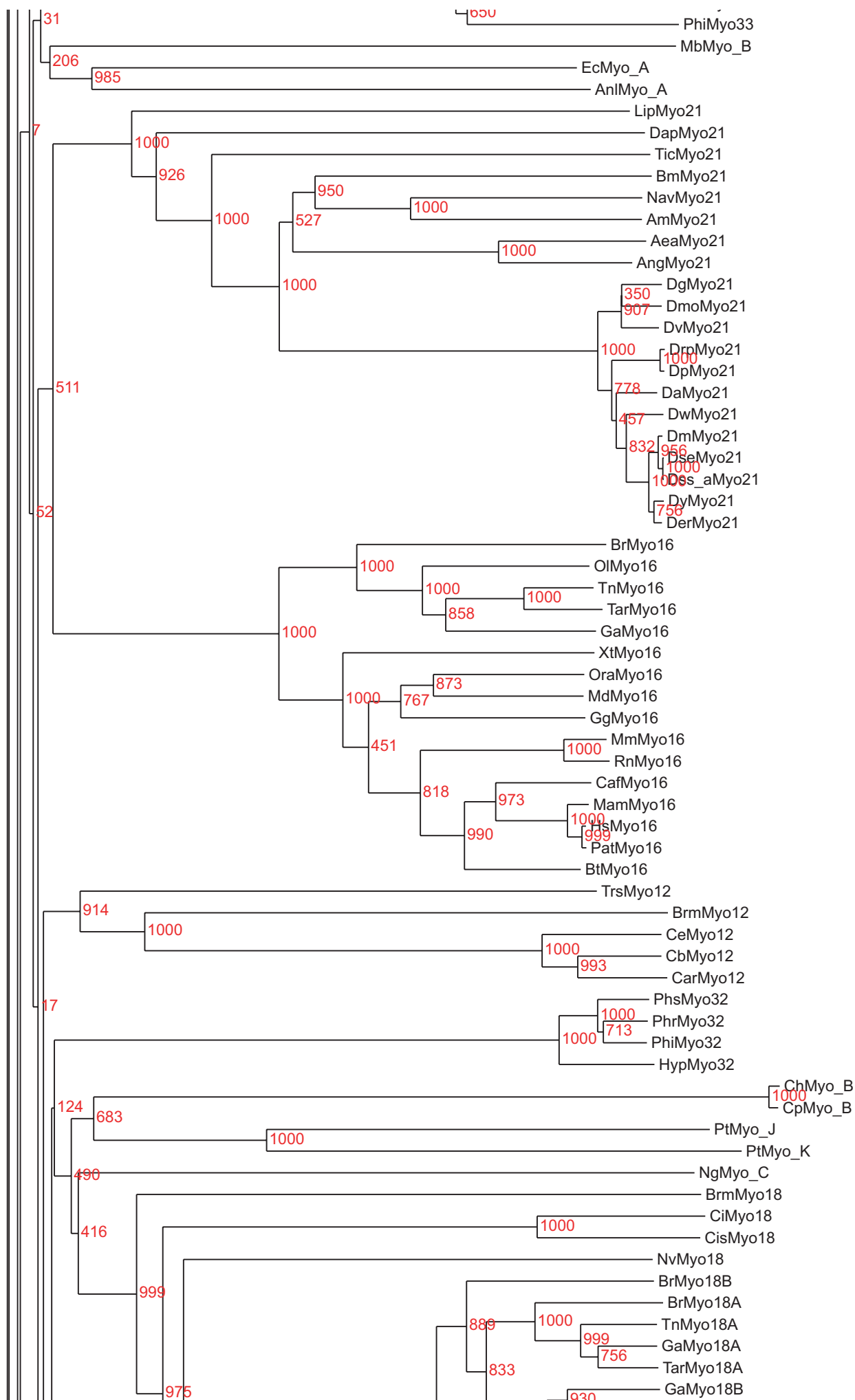

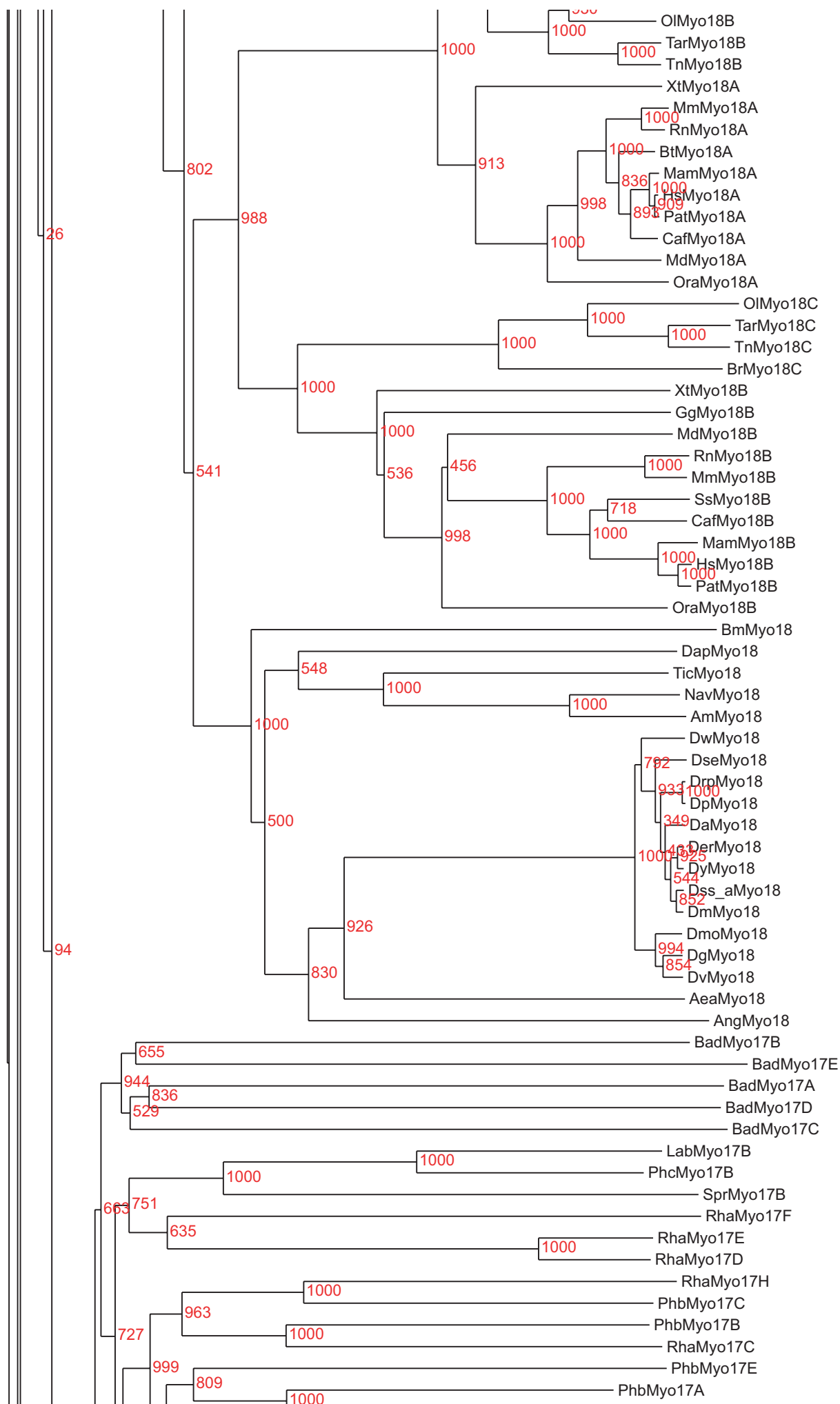

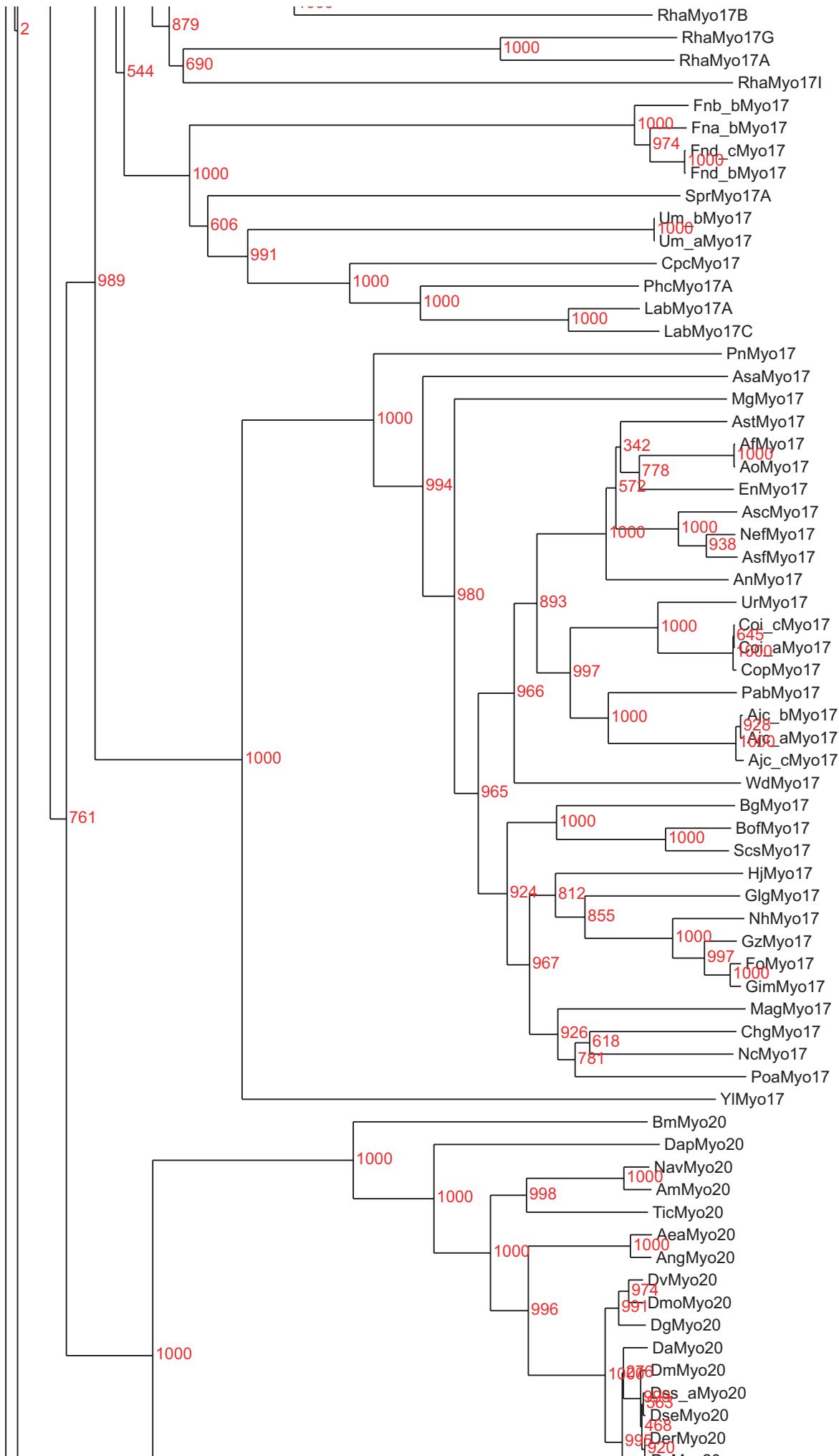

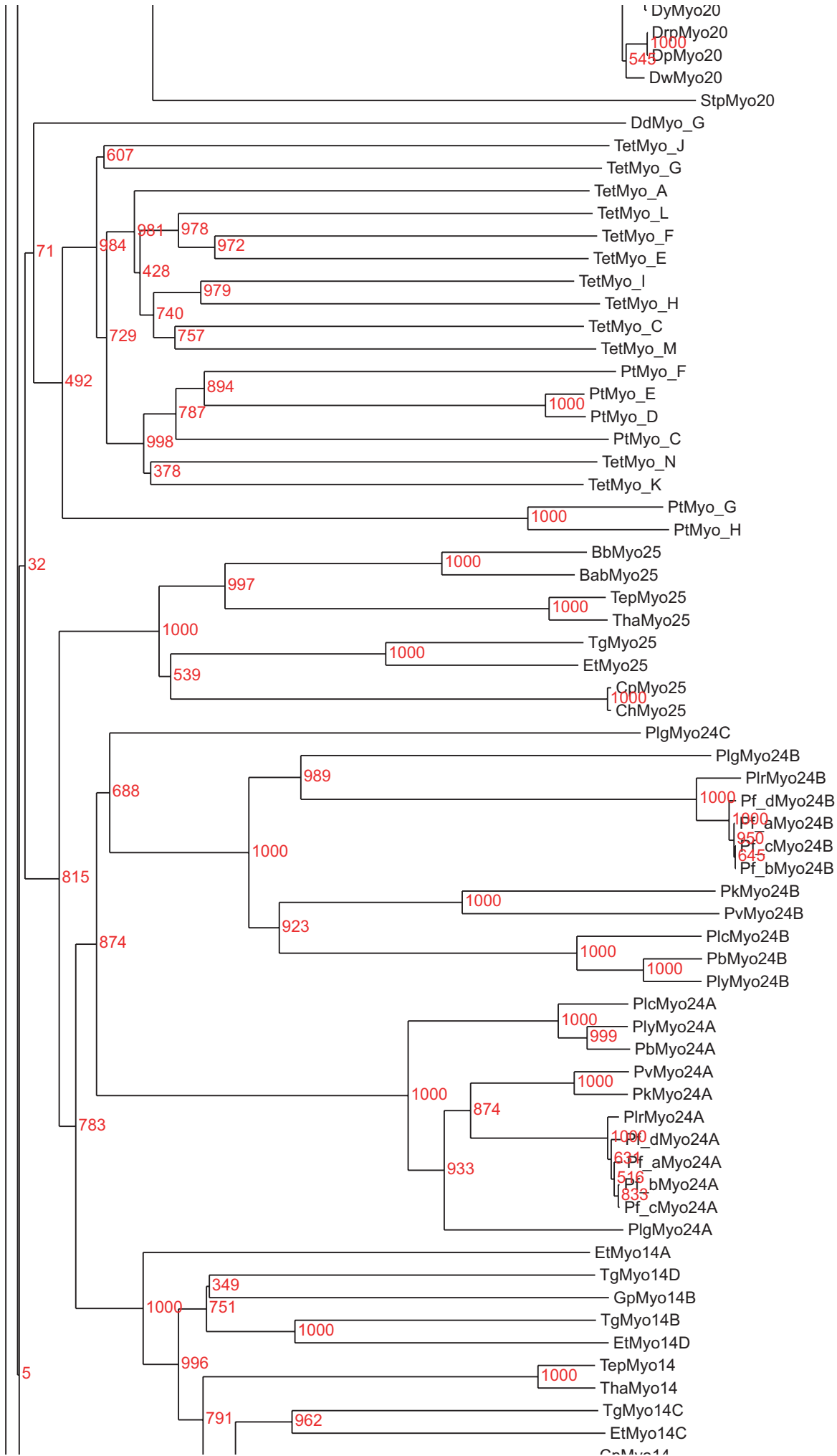

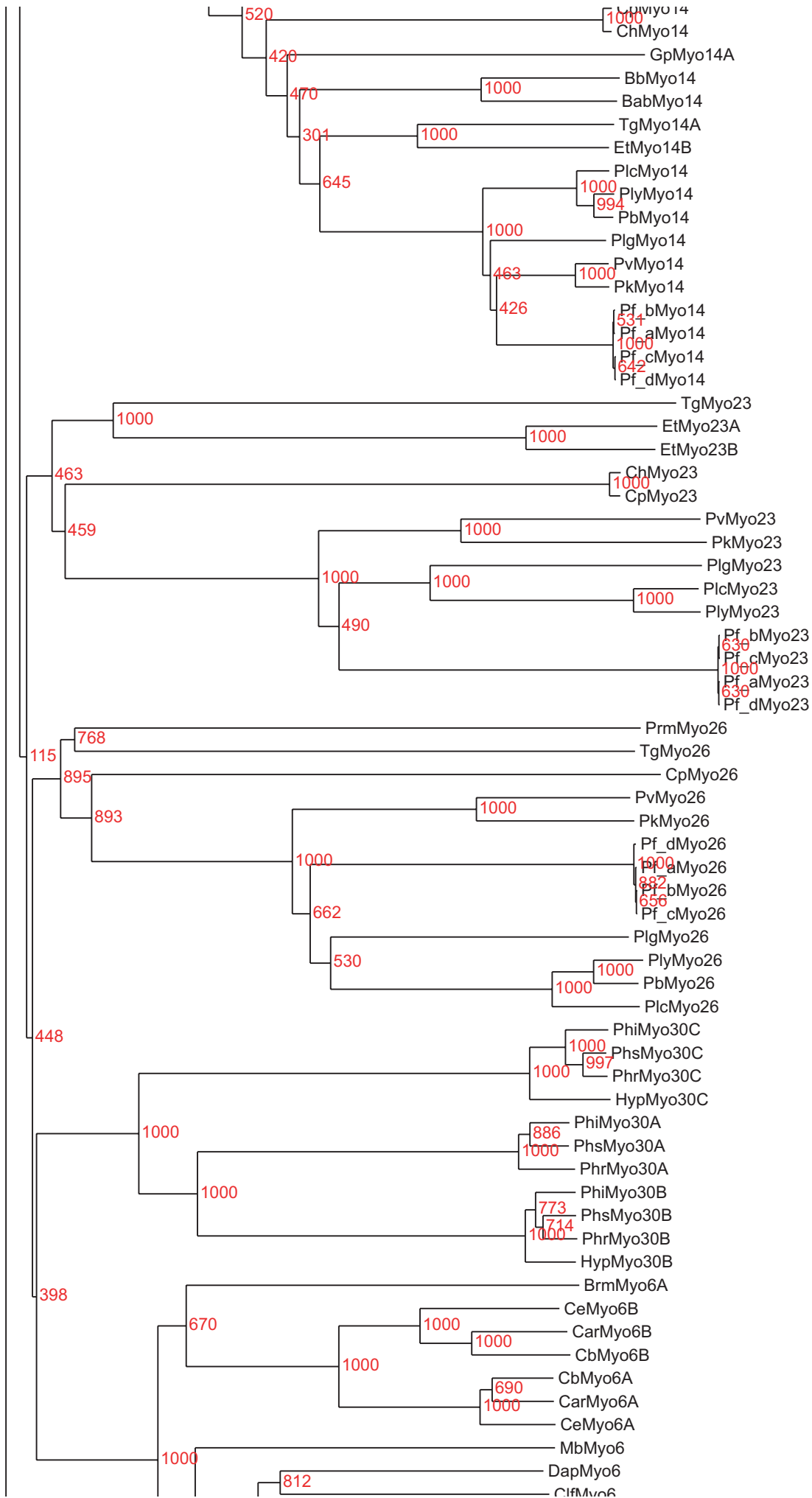

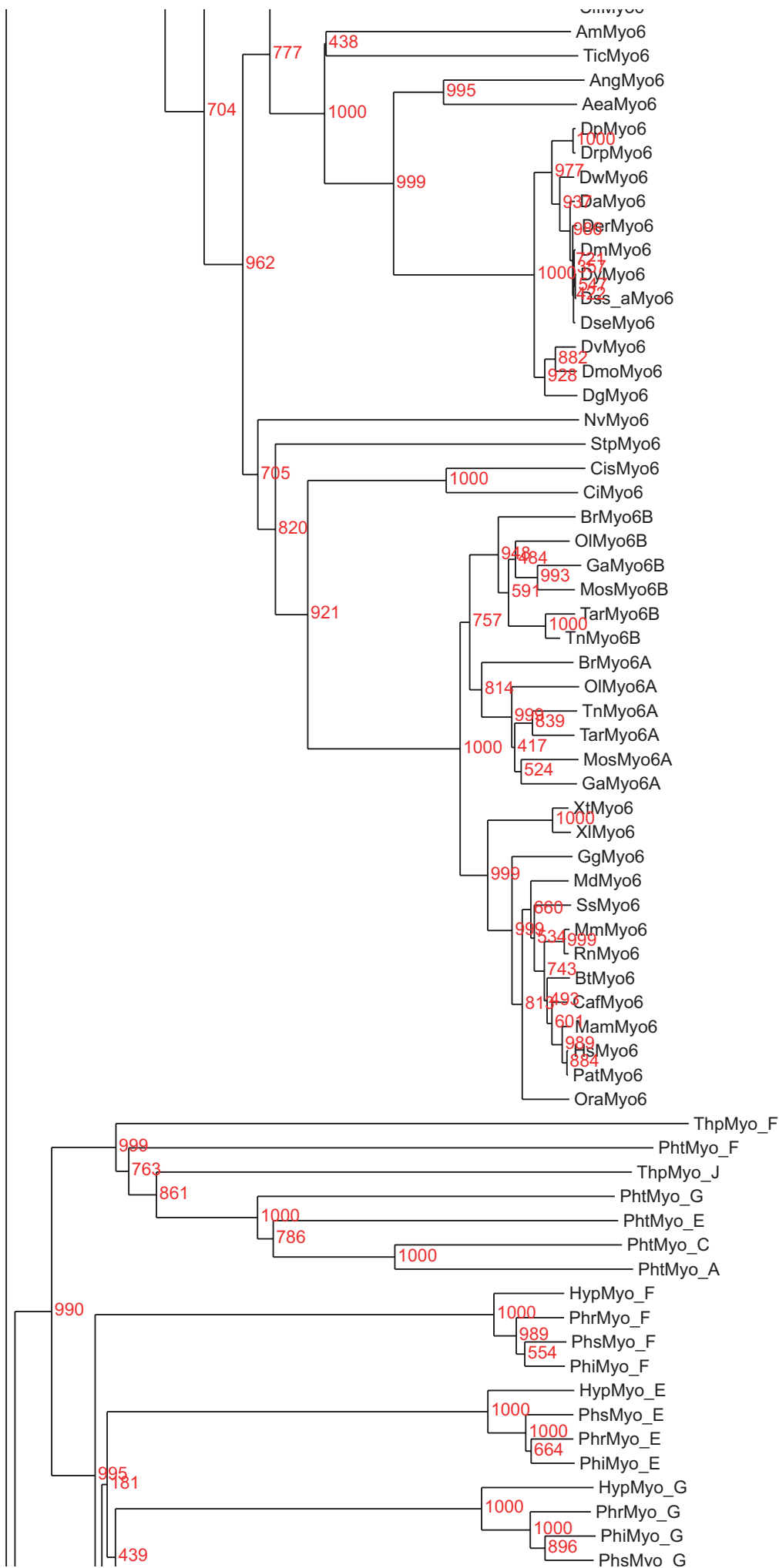

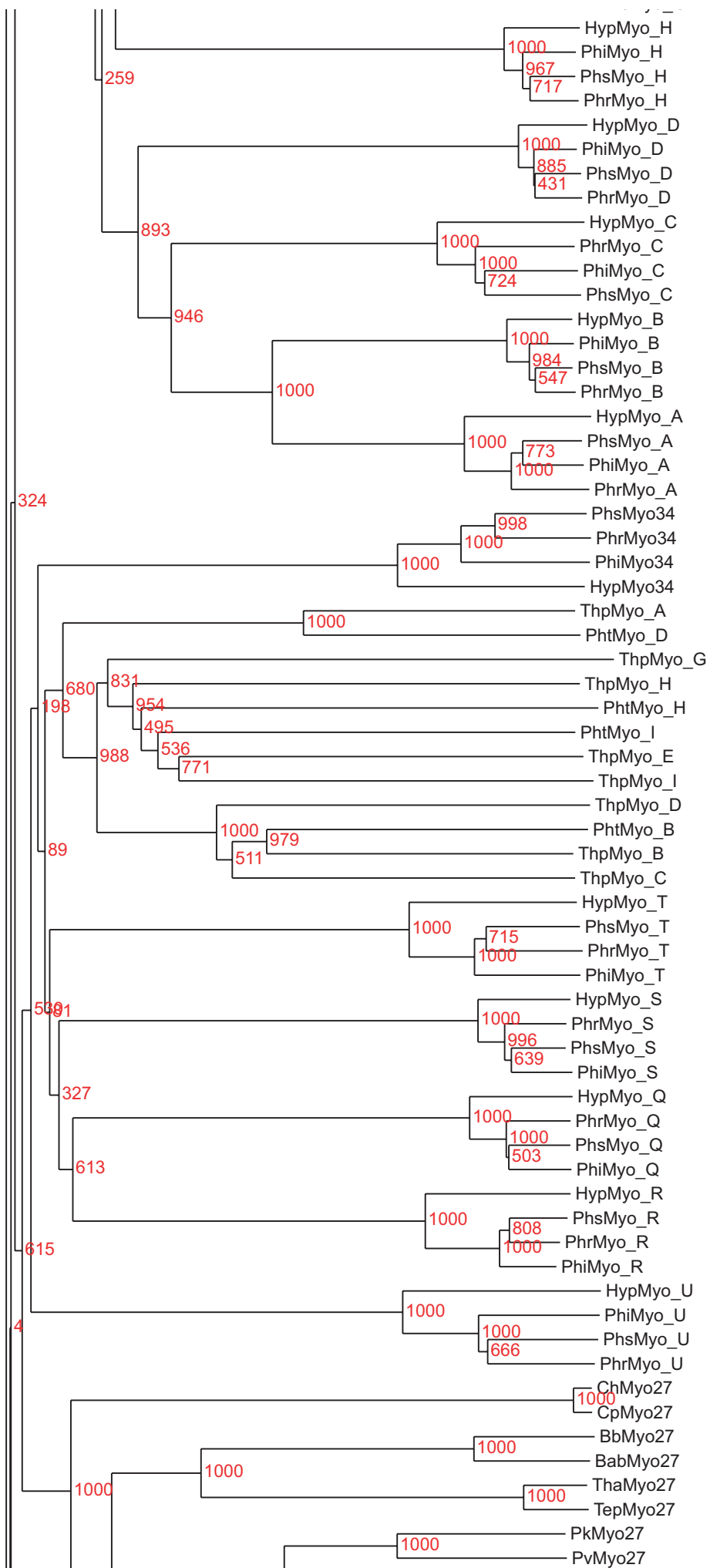

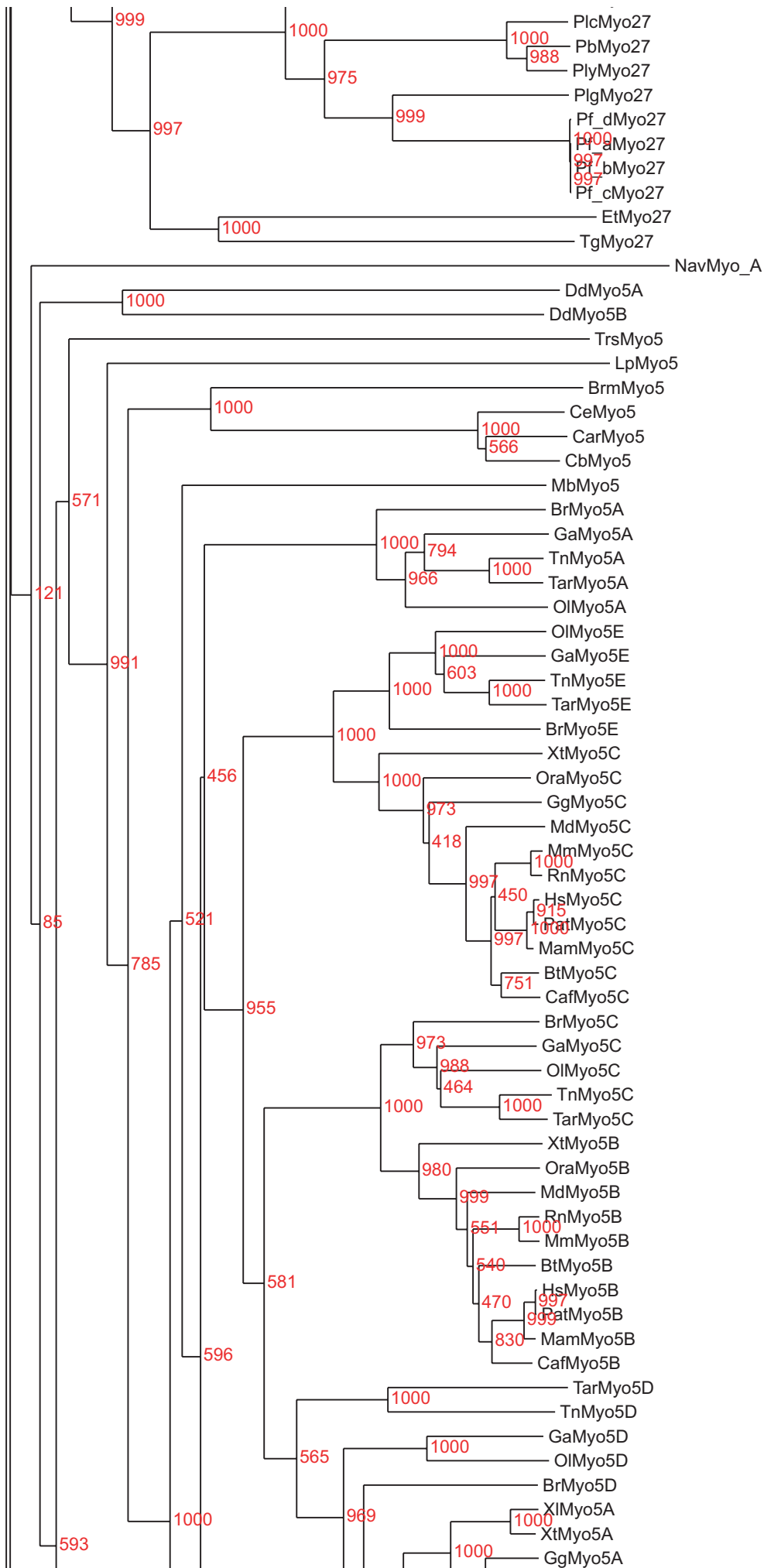

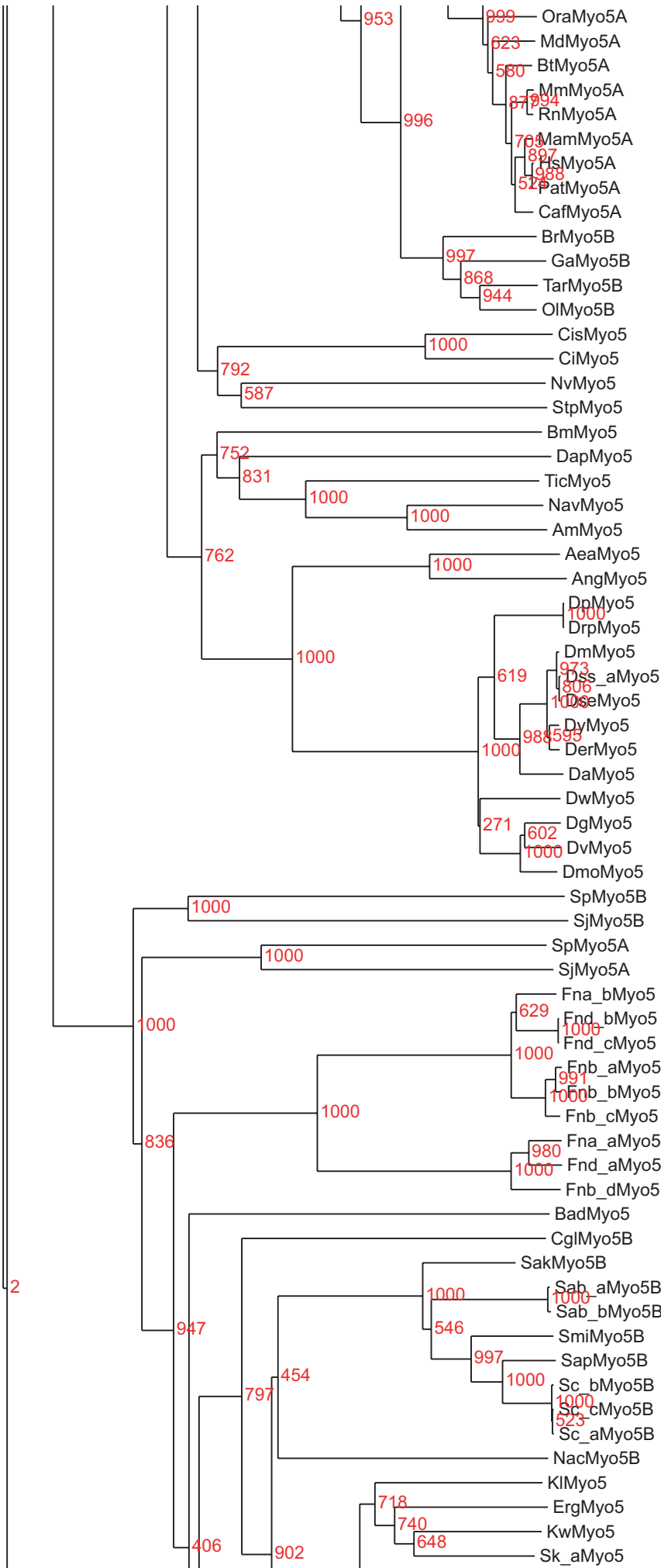

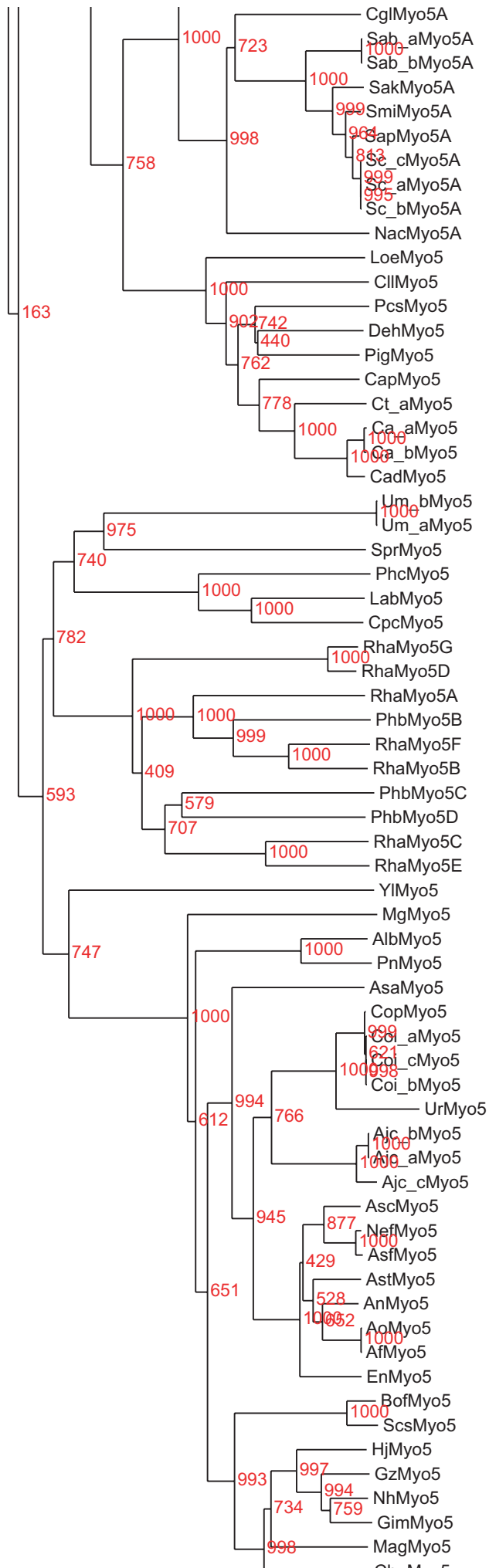

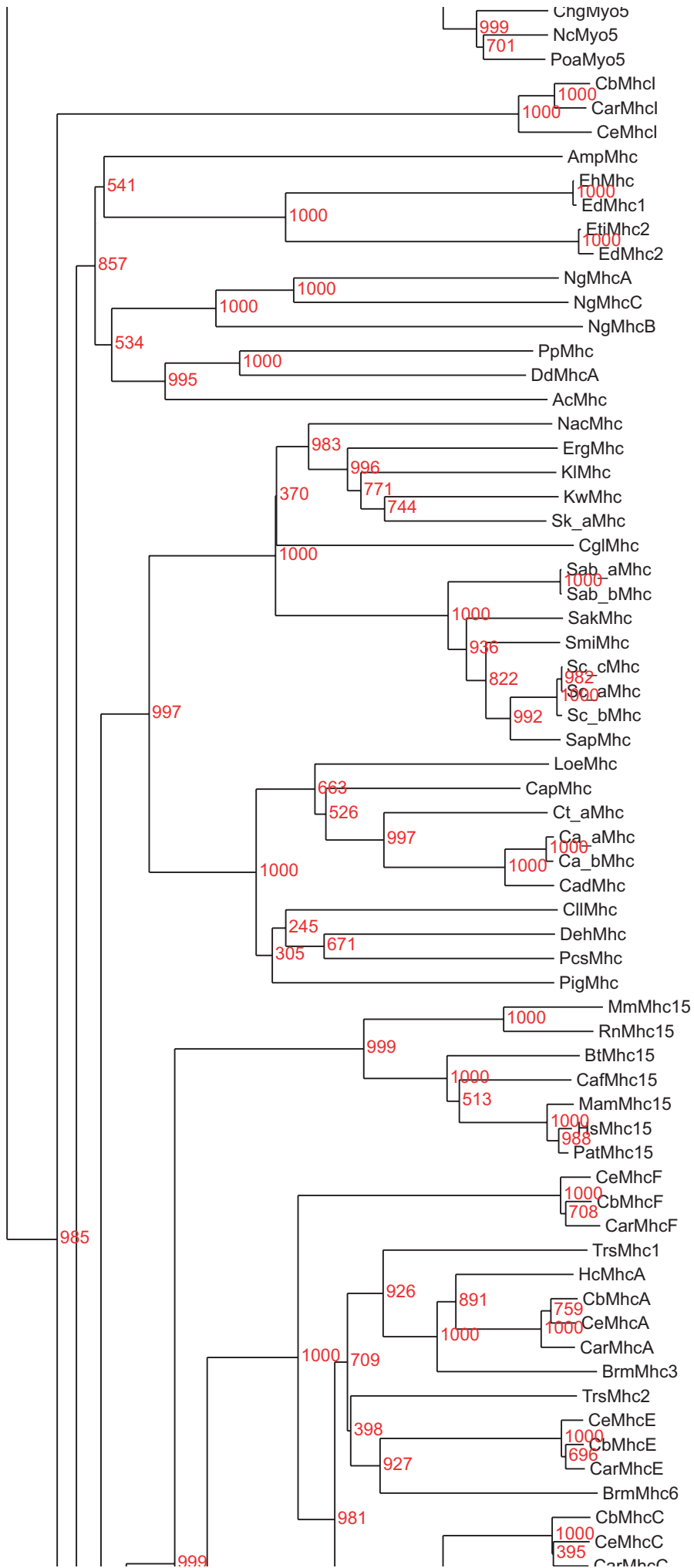

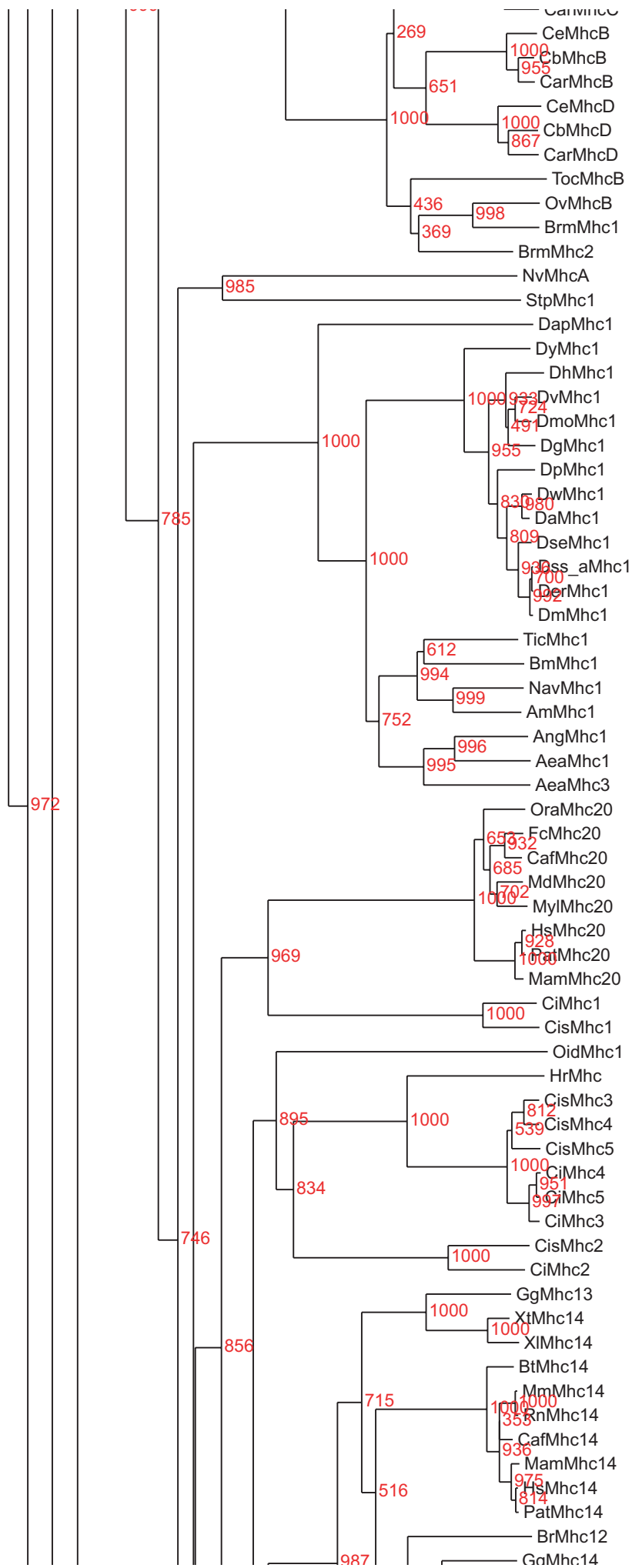

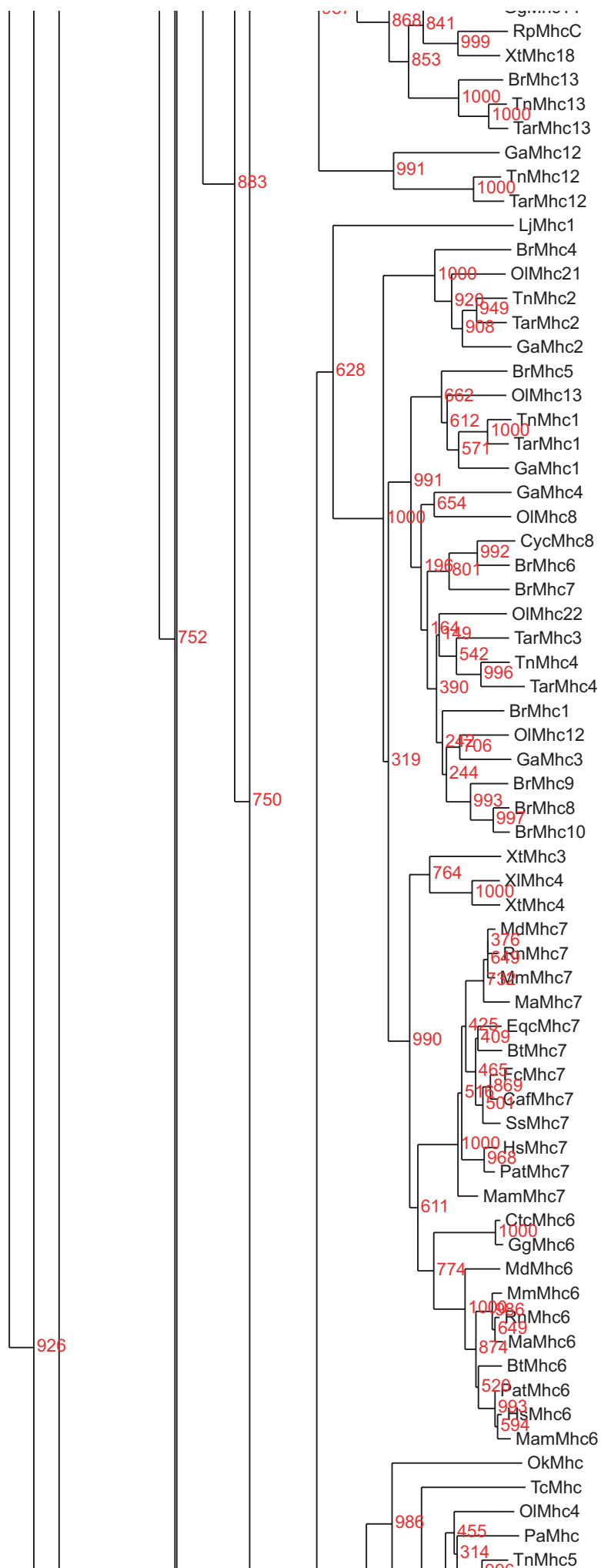

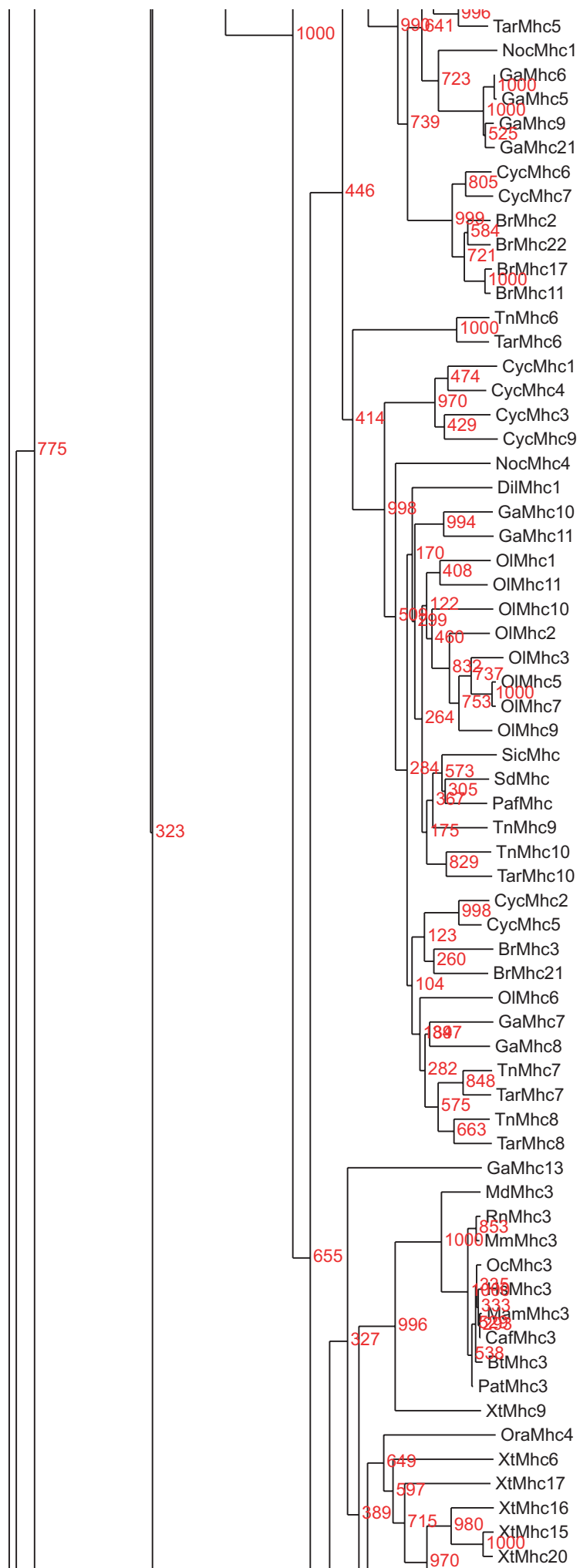

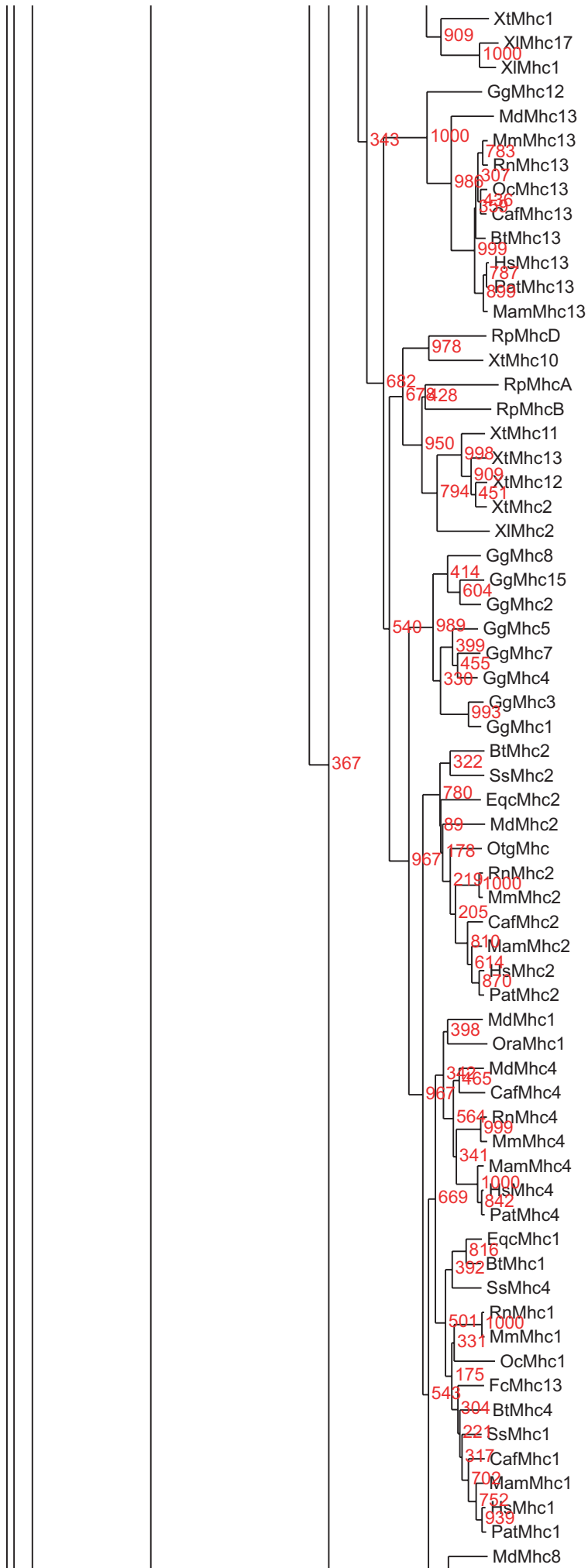

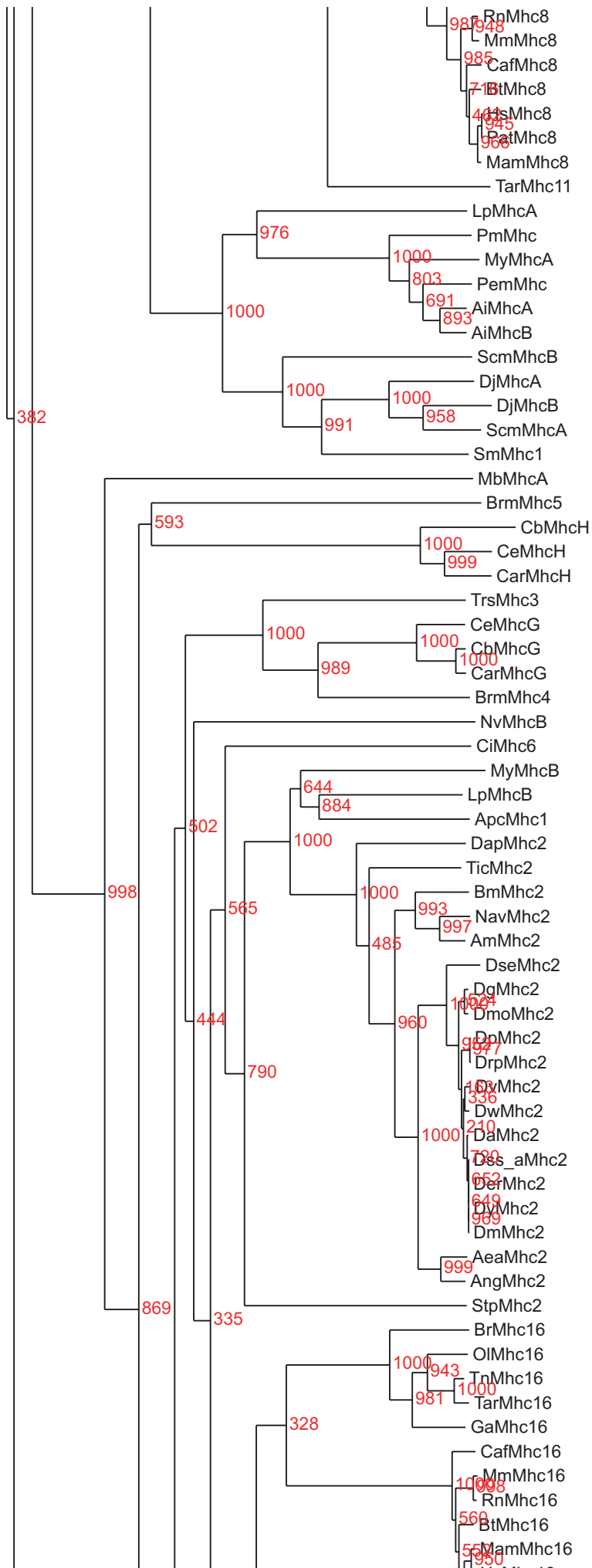

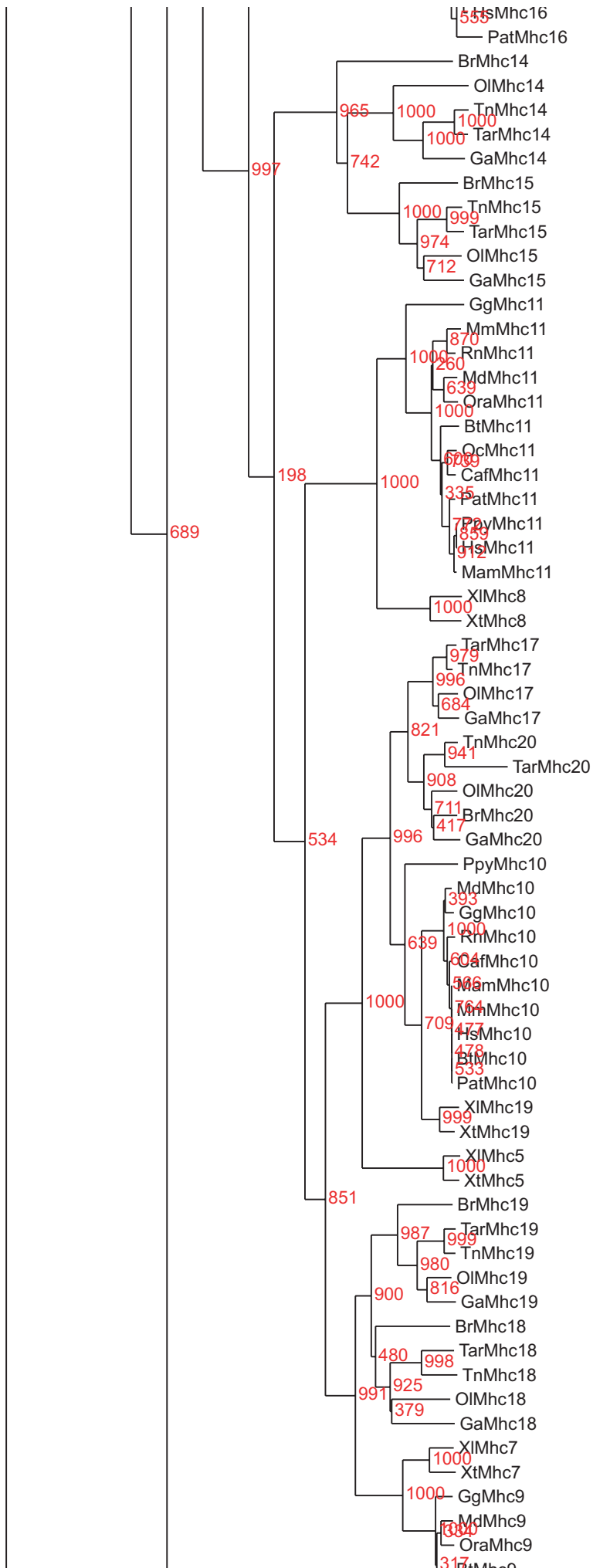

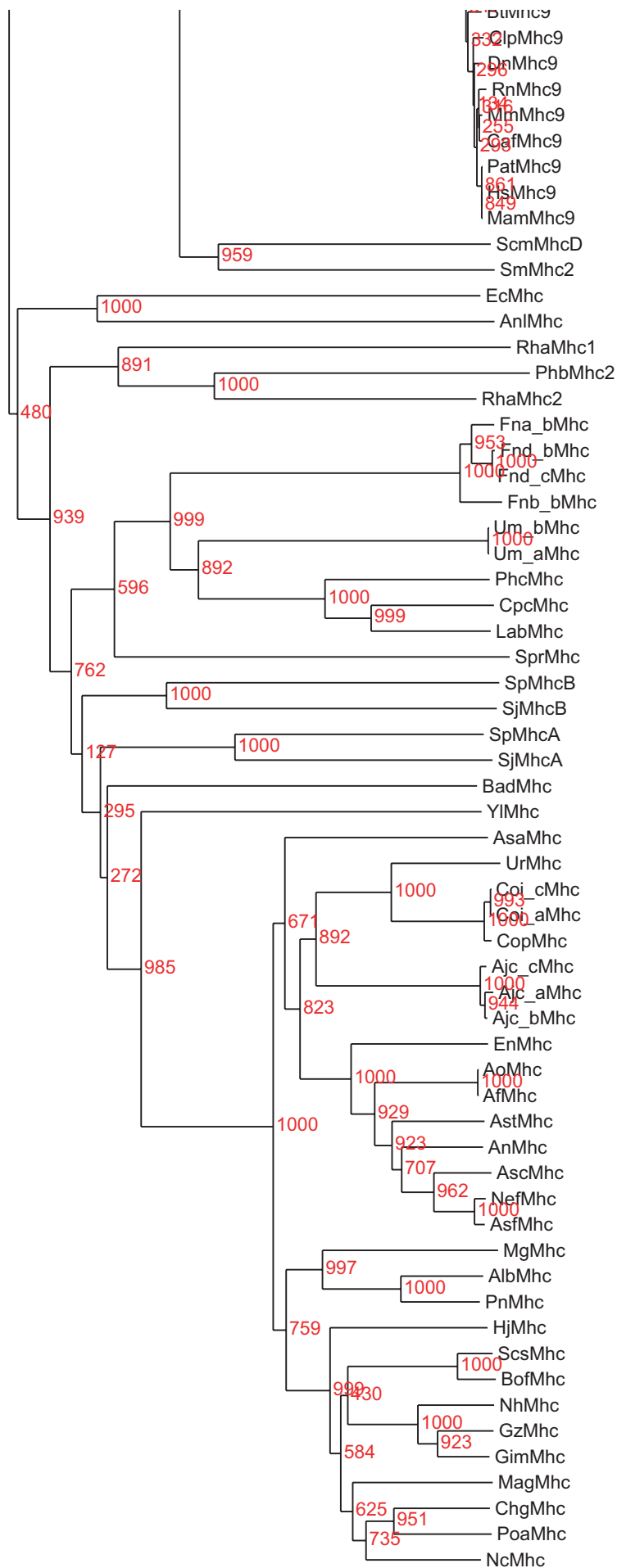

Supplement: Additional data file 2 — Complete phylogenetic tree of 1,984 myosins. [file gb-2007-8-9-r196-S2.pdf]
