# Supplementary material for: Drawing the tree of eukaryotic life based on the analysis of 2,269 manually annotated myosins from 328 species
Source: Genome Biol. 2007 Sep 18;8(9):R196. doi: 10.1186/gb-2007-8-9-r196 (PMC2375034; doi:10.1186/gb-2007-8-9-r196)
Supplement: Additional data file 3 — Complete myosin inventory of all 328 species. [file gb-2007-8-9-r196-S3.pdf]

cellular organisms	Compl.	No. Class	No. Prot	1	2	3	4	5	6	7	8	9	10	11	12	13	14	15	16	17	18	19	20	21	22	23	24	25	26	27	28	29	30	31	32	33	34	35	O
Acanthamoeba castellanii	N	3	5	3	1	-	1	-	-	-	-	-	-	-	-	-	-	-	-	-	-	-	-	-	-	-	-	-	-	-	-	-	-	-	-	-	-	-	-
Monosiga brevicollis	Y	8	29	6	1	-	-	1	1	1	-	-	1	-	-	-	-	1	-	-	-	-	-	-	1	-	-	-	-	-	-	-	-	-	-	-	-	-	16
Naegleria gruberi NEG-M	Y	2	12	6	3	-	-	-	-	-	-	-	-	-	-	-	-	-	-	-	-	-	-	-	-	-	-	-	-	-	-	-	-	-	-	-	-	-	3
Amoeba proteus	N	1	1	-	1	-	-	-	-	-	-	-	-	-	-	-	-	-	-	-	-	-	-	-	-	-	-	-	-	-	-	-	-	-	-	-	-	-	-
Hartmannella vermiformis	N	1	1	1	-	-	-	-	-	-	-	-	-	-	-	-	-	-	-	-	-	-	-	-	-	-	-	-	-	-	-	-	-	-	-	-	-	-	-
Physarum polycephalum	N	1	1	-	1	-	-	-	-	-	-	-	-	-	-	-	-	-	-	-	-	-	-	-	-	-	-	-	-	-	-	-	-	-	-	-	-	-	-

Alveolata	Compl.	No. Class	No. Prot	1	2	3	4	5	6	7	8	9	10	11	12	13	14	15	16	17	18	19	20	21	22	23	24	25	26	27	28	29	30	31	32	33	34	35	O
Tetrahymena thermophila SB210	Y	0	14	-	-	-	-	-	-	-	-	-	-	-	-	-	-	-	-	-	-	-	-	-	-	-	-	-	-	-	-	-	-	-	-	-	-	-	14
Paramecium tetraurelia	Y	0	11	-	-	-	-	-	-	-	-	-	-	-	-	-	-	-	-	-	-	-	-	-	-	-	-	-	-	-	-	-	-	-	-	-	-	-	11
Karlodinium micrum	N	1	1	-	-	-	-	-	-	-	-	-	-	-	-	-	-	-	-	-	-	-	-	-	-	-	-	-	-	1	-	-	-	-	-	-	-	-	-
Perkinsus marinus	N	2	4	-	-	-	-	-	-	-	-	-	-	-	-	-	-	-	-	-	-	-	-	-	-	-	-	-	1	2	-	-	-	-	-	-	-	-	1

Apicomplexa	Compl.	No. Class	No. Prot	1	2	3	4	5	6	7	8	9	10	11	12	13	14	15	16	17	18	19	20	21	22	23	24	25	26	27	28	29	30	31	32	33	34	35	O
Cryptosporidium parvum	Y	5	7	-	-	-	-	-	-	-	-	-	-	-	-	-	1	-	-	-	-	-	-	-	-	1	-	1	1	1	-	-	-	-	-	-	-	-	2
Cryptosporidium hominis TU502	Y	5	7	-	-	-	-	-	-	-	-	-	-	-	-	-	1	-	-	-	-	-	-	-	-	1	-	1	1	1	-	-	-	-	-	-	-	-	2
Eimeria tenella	Y	4	11	-	-	-	-	-	-	-	-	-	-	-	-	-	5	-	-	-	-	-	-	-	-	2	-	1	-	1	-	-	-	-	-	-	-	-	2
Neospora caninum	N	1	1	-	-	-	-	-	-	-	-	-	-	-	-	-	1	-	-	-	-	-	-	-	-	-	-	-	-	-	-	-	-	-	-	-	-	-	-
Toxoplasma gondii RH	Y	5	10	-	-	-	-	-	-	-	-	-	-	-	-	-	4	-	-	-	-	-	-	-	-	1	-	1	1	1	-	-	-	-	-	-	-	-	2
Gregarina polymorpha	N	1	2	-	-	-	-	-	-	-	-	-	-	-	-	-	2	-	-	-	-	-	-	-	-	-	-	-	-	-	-	-	-	-	-	-	-	-	-
Babesia bigemina	Y	3	3	-	-	-	-	-	-	-	-	-	-	-	-	-	1	-	-	-	-	-	-	-	-	-	-	1	-	1	-	-	-	-	-	-	-	-	-
Babesia bovis str. Texas T2Bo	Y	3	3	-	-	-	-	-	-	-	-	-	-	-	-	-	1	-	-	-	-	-	-	-	-	-	-	1	-	1	-	-	-	-	-	-	-	-	-
Theileria annulata str. Ankara	Y	3	3	-	-	-	-	-	-	-	-	-	-	-	-	-	1	-	-	-	-	-	-	-	-	-	-	1	-	1	-	-	-	-	-	-	-	-	-
Theileria parva str. Muguga	Y	3	3	-	-	-	-	-	-	-	-	-	-	-	-	-	1	-	-	-	-	-	-	-	-	-	-	1	-	1	-	-	-	-	-	-	-	-	-

Haemosporida	Compl.	No. Class	No. Prot	1	2	3	4	5	6	7	8	9	10	11	12	13	14	15	16	17	18	19	20	21	22	23	24	25	26	27	28	29	30	31	32	33	34	35	O
Plasmodium gallinaceum	Y	5	7	-	-	-	-	-	-	-	-	-	-	-	-	-	1	-	-	-	-	-	-	-	-	1	3	-	1	1	-	-	-	-	-	-	-	-	-
Plasmodium knowlesi	Y	5	6	-	-	-	-	-	-	-	-	-	-	-	-	-	1	-	-	-	-	-	-	-	-	1	2	-	1	1	-	-	-	-	-	-	-	-	-
Plasmodium reichenowi	N	2	3	-	-	-	-	-	-	-	-	-	-	-	-	-	-	-	-	-	-	-	-	-	-	-	2	-	-	1	-	-	-	-	-	-	-	-	-
Plasmodium berghei str. ANKA	Y	5	6	-	-	-	-	-	-	-	-	-	-	-	-	-	1	-	-	-	-	-	-	-	-	1	2	-	1	1	-	-	-	-	-	-	-	-	-
Plasmodium chabaudi	Y	5	6	-	-	-	-	-	-	-	-	-	-	-	-	-	1	-	-	-	-	-	-	-	-	1	2	-	1	1	-	-	-	-	-	-	-	-	-
Plasmodium vivax str. Salvador I	Y	5	6	-	-	-	-	-	-	-	-	-	-	-	-	-	1	-	-	-	-	-	-	-	-	1	2	-	1	1	-	-	-	-	-	-	-	-	-
Plasmodium falciparum Ghanaian Isolate	Y	5	6	-	-	-	-	-	-	-	-	-	-	-	-	-	1	-	-	-	-	-	-	-	-	1	2	-	1	1	-	-	-	-	-	-	-	-	-
Plasmodium falciparum Dd2	Y	5	6	-	-	-	-	-	-	-	-	-	-	-	-	-	1	-	-	-	-	-	-	-	-	1	2	-	1	1	-	-	-	-	-	-	-	-	-
Plasmodium falciparum HB3	Y	5	6	-	-	-	-	-	-	-	-	-	-	-	-	-	1	-	-	-	-	-	-	-	-	1	2	-	1	1	-	-	-	-	-	-	-	-	-
Plasmodium falciparum 3D7	Y	5	6	-	-	-	-	-	-	-	-	-	-	-	-	-	1	-	-	-	-	-	-	-	-	1	2	-	1	1	-	-	-	-	-	-	-	-	-
Plasmodium yoelii yoelii 17XNL	Y	5	6	-	-	-	-	-	-	-	-	-	-	-	-	-	1	-	-	-	-	-	-	-	-	1	2	-	1	1	-	-	-	-	-	-	-	-	-

Entamoebidae	Compl.	No. Class	No. Prot	1	2	3	4	5	6	7	8	9	10	11	12	13	14	15	16	17	18	19	20	21	22	23	24	25	26	27	28	29	30	31	32	33	34	35	O
Entamoeba histolytica HM-1:IMSS	Y	2	2	1	1	-	-	-	-	-	-	-	-	-	-	-	-	-	-	-	-	-	-	-	-	-	-	-	-	-	-	-	-	-	-	-	-	-	-
Entamoeba dispar SAW760	N	2	3	1	2	-	-	-	-	-	-	-	-	-	-	-	-	-	-	-	-	-	-	-	-	-	-	-	-	-	-	-	-	-	-	-	-	-	-
Entamoeba invadens IP1	N	2	3	1	2	-	-	-	-	-	-	-	-	-	-	-	-	-	-	-	-	-	-	-	-	-	-	-	-	-	-	-	-	-	-	-	-	-	-

Euglenozoa	Compl.	No. Class	No. Prot	1	2	3	4	5	6	7	8	9	10	11	12	13	14	15	16	17	18	19	20	21	22	23	24	25	26	27	28	29	30	31	32	33	34	35	O
Leishmania infantum JPCM5	Y	2	2	1	-	-	-	-	-	-	-	-	-	-	-	1	-	-	-	-	-	-	-	-	-	-	-	-	-	-	-	-	-	-	-	-	-	-	-
Leishmania major str. Friedlin	Y	2	2	1	-	-	-	-	-	-	-	-	-	-	-	1	-	-	-	-	-	-	-	-	-	-	-	-	-	-	-	-	-	-	-	-	-	-	-
Leishmania braziliensis	Y	2	2	1	-	-	-	-	-	-	-	-	-	-	-	1	-	-	-	-	-	-	-	-	-	-	-	-	-	-	-	-	-	-	-	-	-	-	-
Trypanosoma vivax	Y	2	2	1	-	-	-	-	-	-	-	-	-	-	-	1	-	-	-	-	-	-	-	-	-	-	-	-	-	-	-	-	-	-	-	-	-	-	-
Trypanosoma congolense	N	1	1	1	-	-	-	-	-	-	-	-	-	-	-	-	-	-	-	-	-	-	-	-	-	-	-	-	-	-	-	-	-	-	-	-	-	-	-
Trypanosoma cruzi CL Brener	Y	2	17	1	-	-	-	-	-	-	-	-	-	-	-	2	-	-	-	-	-	-	-	-	-	-	-	-	-	-	-	-	-	-	-	-	-	-	14
Trypanosoma brucei TREU927	Y	2	2	1	-	-	-	-	-	-	-	-	-	-	-	1	-	-	-	-	-	-	-	-	-	-	-	-	-	-	-	-	-	-	-	-	-	-	-
Trypanosoma brucei gambiense	Y	2	2	1	-	-	-	-	-	-	-	-	-	-	-	1	-	-	-	-	-	-	-	-	-	-	-	-	-	-	-	-	-	-	-	-	-	-	-

Ascomycota	Compl.	No. Class	No. Prot	1	2	3	4	5	6	7	8	9	10	11	12	13	14	15	16	17	18	19	20	21	22	23	24	25	26	27	28	29	30	31	32	33	34	35	O
Wangiella dermatitidis	N	1	1	-	-	-	-	-	-	-	-	-	-	-	-	-	-	-	-	1	-	-	-	-	-	-	-	-	-	-	-	-	-	-	-	-	-	-	-

Mycosphaerella graminicola	Y	4	4	1	1	-	-	1	-	-	-	-	-	-	-	-	-	-	-	1	-	-	-	-	-	-	-	-	-	-	-	-	-	-	-	-	-	-	-
Phaeosphaeria nodorum SN15	Y	4	4	1	1	-	-	1	-	-	-	-	-	-	-	-	-	-	-	1	-	-	-	-	-	-	-	-	-	-	-	-	-	-	-	-	-	-	-
Alternaria brassicicola ATCC 96836	Y	4	4	1	1	-	-	1	-	-	-	-	-	-	-	-	-	-	-	1	-	-	-	-	-	-	-	-	-	-	-	-	-	-	-	-	-	-	-
Blumeria graminis	N	1	1	-	-	-	-	-	-	-	-	-	-	-	-	-	-	-	-	1	-	-	-	-	-	-	-	-	-	-	-	-	-	-	-	-	-	-	-
Botryotinia fuckeliana B05.10	Y	4	4	1	1	-	-	1	-	-	-	-	-	-	-	-	-	-	-	1	-	-	-	-	-	-	-	-	-	-	-	-	-	-	-	-	-	-	-
Sclerotinia sclerotiorum 1980	Y	4	4	1	1	-	-	1	-	-	-	-	-	-	-	-	-	-	-	1	-	-	-	-	-	-	-	-	-	-	-	-	-	-	-	-	-	-	-
Hypocrea jecorina QM9414	Y	4	4	1	1	-	-	1	-	-	-	-	-	-	-	-	-	-	-	1	-	-	-	-	-	-	-	-	-	-	-	-	-	-	-	-	-	-	-
Fusarium oxysporum	N	1	1	-	-	-	-	-	-	-	-	-	-	-	-	-	-	-	-	1	-	-	-	-	-	-	-	-	-	-	-	-	-	-	-	-	-	-	-
Gibberella zeae PH-1	Y	4	4	1	1	-	-	1	-	-	-	-	-	-	-	-	-	-	-	1	-	-	-	-	-	-	-	-	-	-	-	-	-	-	-	-	-	-	-
Gibberella moniliformis 7600	Y	4	4	1	1	-	-	1	-	-	-	-	-	-	-	-	-	-	-	1	-	-	-	-	-	-	-	-	-	-	-	-	-	-	-	-	-	-	-
Nectria haematococca MPVI	Y	4	4	1	1	-	-	1	-	-	-	-	-	-	-	-	-	-	-	1	-	-	-	-	-	-	-	-	-	-	-	-	-	-	-	-	-	-	-
Magnaporthe grisea 70-15	Y	4	4	1	1	-	-	1	-	-	-	-	-	-	-	-	-	-	-	1	-	-	-	-	-	-	-	-	-	-	-	-	-	-	-	-	-	-	-
Glomerella graminicola	N	1	1	-	-	-	-	-	-	-	-	-	-	-	-	-	-	-	-	1	-	-	-	-	-	-	-	-	-	-	-	-	-	-	-	-	-	-	-
Colletotrichum trifolii	N	1	1	-	-	-	-	1	-	-	-	-	-	-	-	-	-	-	-	-	-	-	-	-	-	-	-	-	-	-	-	-	-	-	-	-	-	-	-
Chaetomium globosum CBS 148.51	Y	4	4	1	1	-	-	1	-	-	-	-	-	-	-	-	-	-	-	1	-	-	-	-	-	-	-	-	-	-	-	-	-	-	-	-	-	-	-
Podospora anserina	Y	4	4	1	1	-	-	1	-	-	-	-	-	-	-	-	-	-	-	1	-	-	-	-	-	-	-	-	-	-	-	-	-	-	-	-	-	-	-
Neurospora crassa OR74A	Y	4	4	1	1	-	-	1	-	-	-	-	-	-	-	-	-	-	-	1	-	-	-	-	-	-	-	-	-	-	-	-	-	-	-	-	-	-	-
Schizosaccharomyces pombe 972h-	Y	3	5	1	2	-	-	2	-	-	-	-	-	-	-	-	-	-	-	-	-	-	-	-	-	-	-	-	-	-	-	-	-	-	-	-	-	-	-
Schizosaccharomyces japonicus yFS275	Y	3	5	1	2	-	-	2	-	-	-	-	-	-	-	-	-	-	-	-	-	-	-	-	-	-	-	-	-	-	-	-	-	-	-	-	-	-	-
Eurotiomycetes	Compl.	No. Class	No. Prot	1	2	3	4	5	6	7	8	9	10	11	12	13	14	15	16	17	18	19	20	21	22	23	24	25	26	27	28	29	30	31	32	33	34	35	O
Emericella nidulans FGSC A4	Y	4	4	1	1	-	-	1	-	-	-	-	-	-	-	-	-	-	-	1	-	-	-	-	-	-	-	-	-	-	-	-	-	-	-	-	-	-	-
Aspergillus fumigatus Af293	Y	4	4	1	1	-	-	1	-	-	-	-	-	-	-	-	-	-	-	1	-	-	-	-	-	-	-	-	-	-	-	-	-	-	-	-	-	-	-
Aspergillus niger ATCC 1015	Y	4	4	1	1	-	-	1	-	-	-	-	-	-	-	-	-	-	-	1	-	-	-	-	-	-	-	-	-	-	-	-	-	-	-	-	-	-	-
Aspergillus flavus NRRL3357	Y	4	4	1	1	-	-	1	-	-	-	-	-	-	-	-	-	-	-	1	-	-	-	-	-	-	-	-	-	-	-	-	-	-	-	-	-	-	-
Aspergillus clavatus NRRL 1	Y	4	4	1	1	-	-	1	-	-	-	-	-	-	-	-	-	-	-	1	-	-	-	-	-	-	-	-	-	-	-	-	-	-	-	-	-	-	-
Aspergillus terreus NIH2624	Y	4	4	1	1	-	-	1	-	-	-	-	-	-	-	-	-	-	-	1	-	-	-	-	-	-	-	-	-	-	-	-	-	-	-	-	-	-	-
Aspergillus oryzae RIB40	Y	4	4	1	1	-	-	1	-	-	-	-	-	-	-	-	-	-	-	1	-	-	-	-	-	-	-	-	-	-	-	-	-	-	-	-	-	-	-
Neosartorya fischeri NRRL 181	Y	4	4	1	1	-	-	1	-	-	-	-	-	-	-	-	-	-	-	1	-	-	-	-	-	-	-	-	-	-	-	-	-	-	-	-	-	-	-
Ajellomyces capsulatus NAmI WU24	Y	4	4	1	1	-	-	1	-	-	-	-	-	-	-	-	-	-	-	1	-	-	-	-	-	-	-	-	-	-	-	-	-	-	-	-	-	-	-
Ajellomyces capsulatus NAmII G186AR	Y	4	4	1	1	-	-	1	-	-	-	-	-	-	-	-	-	-	-	1	-	-	-	-	-	-	-	-	-	-	-	-	-	-	-	-	-	-	-
Ajellomyces capsulatus NAmII G217B	Y	4	4	1	1	-	-	1	-	-	-	-	-	-	-	-	-	-	-	1	-	-	-	-	-	-	-	-	-	-	-	-	-	-	-	-	-	-	-
Ascosphaera apis USDA-ARSEF 7405	Y	4	4	1	1	-	-	1	-	-	-	-	-	-	-	-	-	-	-	1	-	-	-	-	-	-	-	-	-	-	-	-	-	-	-	-	-	-	-
Coccidioides immitis RS	Y	4	4	1	1	-	-	1	-	-	-	-	-	-	-	-	-	-	-	1	-	-	-	-	-	-	-	-	-	-	-	-	-	-	-	-	-	-	-
Coccidioides immitis RMSCC 2394	Y	4	4	1	1	-	-	1	-	-	-	-	-	-	-	-	-	-	-	1	-	-	-	-	-	-	-	-	-	-	-	-	-	-	-	-	-	-	-
Coccidioides immitis H538.4	Y	4	4	1	1	-	-	1	-	-	-	-	-	-	-	-	-	-	-	1	-	-	-	-	-	-	-	-	-	-	-	-	-	-	-	-	-	-	-
Coccidioides posadasii C735	Y	4	4	1	1	-	-	1	-	-	-	-	-	-	-	-	-	-	-	1	-	-	-	-	-	-	-	-	-	-	-	-	-	-	-	-	-	-	-
Paracoccidioides brasiliensis	N	1	1	-	-	-	-	-	-	-	-	-	-	-	-	-	-	-	-	1	-	-	-	-	-	-	-	-	-	-	-	-	-	-	-	-	-	-	-
Uncinocarpus reesii 1704	Y	4	4	1	1	-	-	1	-	-	-	-	-	-	-	-	-	-	-	1	-	-	-	-	-	-	-	-	-	-	-	-	-	-	-	-	-	-	-
Saccharomycetes	Compl.	No. Class	No. Prot	1	2	3	4	5	6	7	8	9	10	11	12	13	14	15	16	17	18	19	20	21	22	23	24	25	26	27	28	29	30	31	32	33	34	35	O
Yarrowia lipolytica CLIB99	Y	4	4	1	1	-	-	1	-	-	-	-	-	-	-	-	-	-	-	1	-	-	-	-	-	-	-	-	-	-	-	-	-	-	-	-	-	-	-
Clavispora lusitaniae ATCC 42720	Y	3	3	1	1	-	-	1	-	-	-	-	-	-	-	-	-	-	-	-	-	-	-	-	-	-	-	-	-	-	-	-	-	-	-	-	-	-	-
Candida glabrata CBS138	Y	3	5	2	1	-	-	2	-	-	-	-	-	-	-	-	-	-	-	-	-	-	-	-	-	-	-	-	-	-	-	-	-	-	-	-	-	-	-
Candida dubliniensis CD36	Y	3	3	1	1	-	-	1	-	-	-	-	-	-	-	-	-	-	-	-	-	-	-	-	-	-	-	-	-	-	-	-	-	-	-	-	-	-	-
Candida parapsilosis	Y	3	3	1	1	-	-	1	-	-	-	-	-	-	-	-	-	-	-	-	-	-	-	-	-	-	-	-	-	-	-	-	-	-	-	-	-	-	-
Candida albicans SC5314	Y	3	4	2	1	-	-	1	-	-	-	-	-	-	-	-	-	-	-	-	-	-	-	-	-	-	-	-	-	-	-	-	-	-	-	-	-	-	-
Candida albicans WO-1	Y	3	3	1	1	-	-	1	-	-	-	-	-	-	-	-	-	-	-	-	-	-	-	-	-	-	-	-	-	-	-	-	-	-	-	-	-	-	-

Candida tropicalis MYA-3404	Y	3	3	1	1	-	-	1	-	-	-	-	-	-	-	-	-	-	-	-	-	-	-	-	-	-	-	-	-	-	-	-	-	-	-	-	-	-	-
Debaryomyces hansenii CBS767	Y	3	3	1	1	-	-	1	-	-	-	-	-	-	-	-	-	-	-	-	-	-	-	-	-	-	-	-	-	-	-	-	-	-	-	-	-	-	-
Eremothecium gossypii ATCC 10895	Y	3	3	1	1	-	-	1	-	-	-	-	-	-	-	-	-	-	-	-	-	-	-	-	-	-	-	-	-	-	-	-	-	-	-	-	-	-	-
Kluyveromyces thermotolerans CBS 6340	N	1	1	1	-	-	-	-	-	-	-	-	-	-	-	-	-	-	-	-	-	-	-	-	-	-	-	-	-	-	-	-	-	-	-	-	-	-	-
Kluyveromyces lactis NRRL Y-1140	Y	3	3	1	1	-	-	1	-	-	-	-	-	-	-	-	-	-	-	-	-	-	-	-	-	-	-	-	-	-	-	-	-	-	-	-	-	-	-
Kluyveromyces waltii NCYC 2644	Y	3	3	1	1	-	-	1	-	-	-	-	-	-	-	-	-	-	-	-	-	-	-	-	-	-	-	-	-	-	-	-	-	-	-	-	-	-	-
Kluyveromyces marxianus CBS712	N	2	2	1	-	-	-	1	-	-	-	-	-	-	-	-	-	-	-	-	-	-	-	-	-	-	-	-	-	-	-	-	-	-	-	-	-	-	-
Lodderomyces elongisporus NRLL YB-4239	Y	3	3	1	1	-	-	1	-	-	-	-	-	-	-	-	-	-	-	-	-	-	-	-	-	-	-	-	-	-	-	-	-	-	-	-	-	-	-
Naumovia castellii NRRL Y-12630	Y	3	4	1	1	-	-	2	-	-	-	-	-	-	-	-	-	-	-	-	-	-	-	-	-	-	-	-	-	-	-	-	-	-	-	-	-	-	-
Pichia guilliermondii ATCC 6260	Y	3	3	1	1	-	-	1	-	-	-	-	-	-	-	-	-	-	-	-	-	-	-	-	-	-	-	-	-	-	-	-	-	-	-	-	-	-	-
Pichia farinosa CBS7064	N	3	3	1	1	-	-	1	-	-	-	-	-	-	-	-	-	-	-	-	-	-	-	-	-	-	-	-	-	-	-	-	-	-	-	-	-	-	-
Pichia stipitis CBS 6054	Y	3	3	1	1	-	-	1	-	-	-	-	-	-	-	-	-	-	-	-	-	-	-	-	-	-	-	-	-	-	-	-	-	-	-	-	-	-	-
Pichia angusta CBS 4732	N	2	2	-	1	-	-	1	-	-	-	-	-	-	-	-	-	-	-	-	-	-	-	-	-	-	-	-	-	-	-	-	-	-	-	-	-	-	-
Saccharomyces mikatae IFO 1815	Y	3	5	2	1	-	-	2	-	-	-	-	-	-	-	-	-	-	-	-	-	-	-	-	-	-	-	-	-	-	-	-	-	-	-	-	-	-	-
Saccharomyces kudriavzevii IFO 1802	Y	3	5	2	1	-	-	2	-	-	-	-	-	-	-	-	-	-	-	-	-	-	-	-	-	-	-	-	-	-	-	-	-	-	-	-	-	-	-
Saccharomyces paradoxus NRRL Y-17217	Y	3	5	2	1	-	-	2	-	-	-	-	-	-	-	-	-	-	-	-	-	-	-	-	-	-	-	-	-	-	-	-	-	-	-	-	-	-	-
Saccharomyces servazzii CBS4311	N	1	1	1	-	-	-	-	-	-	-	-	-	-	-	-	-	-	-	-	-	-	-	-	-	-	-	-	-	-	-	-	-	-	-	-	-	-	-
Kazachstania exigua CBS379	N	1	2	-	-	-	-	2	-	-	-	-	-	-	-	-	-	-	-	-	-	-	-	-	-	-	-	-	-	-	-	-	-	-	-	-	-	-	-
Saccharomyces bayanus MCYC 623	Y	3	5	2	1	-	-	2	-	-	-	-	-	-	-	-	-	-	-	-	-	-	-	-	-	-	-	-	-	-	-	-	-	-	-	-	-	-	-
Saccharomyces bayanus var. uvarum	N	1	1	-	-	-	-	1	-	-	-	-	-	-	-	-	-	-	-	-	-	-	-	-	-	-	-	-	-	-	-	-	-	-	-	-	-	-	-
Saccharomyces bayanus 623-6C	Y	3	5	2	1	-	-	2	-	-	-	-	-	-	-	-	-	-	-	-	-	-	-	-	-	-	-	-	-	-	-	-	-	-	-	-	-	-	-
Saccharomyces cerevisiae RM11-1a	Y	3	5	2	1	-	-	2	-	-	-	-	-	-	-	-	-	-	-	-	-	-	-	-	-	-	-	-	-	-	-	-	-	-	-	-	-	-	-
Saccharomyces cerevisiae YJM789	Y	3	5	2	1	-	-	2	-	-	-	-	-	-	-	-	-	-	-	-	-	-	-	-	-	-	-	-	-	-	-	-	-	-	-	-	-	-	-
Saccharomyces cerevisiae S288c	Y	3	5	2	1	-	-	2	-	-	-	-	-	-	-	-	-	-	-	-	-	-	-	-	-	-	-	-	-	-	-	-	-	-	-	-	-	-	-
Saccharomyces kluyveri NRRL Y-12651	Y	3	3	1	1	-	-	1	-	-	-	-	-	-	-	-	-	-	-	-	-	-	-	-	-	-	-	-	-	-	-	-	-	-	-	-	-	-	-
Zygosaccharomyces rouxii CBS732	N	2	2	1	-	-	-	1	-	-	-	-	-	-	-	-	-	-	-	-	-	-	-	-	-	-	-	-	-	-	-	-	-	-	-	-	-	-	-
Basidiomycota	Compl.	No. Class	No. Prot	1	2	3	4	5	6	7	8	9	10	11	12	13	14	15	16	17	18	19	20	21	22	23	24	25	26	27	28	29	30	31	32	33	34	35	O
Filobasidiella neoformans var. bacillispora WM276	N	1	1	-	-	-	-	1	-	-	-	-	-	-	-	-	-	-	-	-	-	-	-	-	-	-	-	-	-	-	-	-	-	-	-	-	-	-	-
Filobasidiella neoformans var. bacillispora ATCC32609	N	1	1	-	-	-	-	1	-	-	-	-	-	-	-	-	-	-	-	-	-	-	-	-	-	-	-	-	-	-	-	-	-	-	-	-	-	-	-
Filobasidiella neoformans var. bacillispora R265	Y	4	4	1	1	-	-	1	-	-	-	-	-	-	-	-	-	-	-	1	-	-	-	-	-	-	-	-	-	-	-	-	-	-	-	-	-	-	-
Filobasidiella neoformans var. bacillispora E566	N	1	1	-	-	-	-	1	-	-	-	-	-	-	-	-	-	-	-	-	-	-	-	-	-	-	-	-	-	-	-	-	-	-	-	-	-	-	-
Filobasidiella neoformans var. neoformans 125.91	N	1	1	-	-	-	-	1	-	-	-	-	-	-	-	-	-	-	-	-	-	-	-	-	-	-	-	-	-	-	-	-	-	-	-	-	-	-	-
Filobasidiella neoformans var. neoformans H99	Y	4	4	1	1	-	-	1	-	-	-	-	-	-	-	-	-	-	-	1	-	-	-	-	-	-	-	-	-	-	-	-	-	-	-	-	-	-	-
Filobasidiella neoformans var. neoformans B-3501A	Y	4	4	1	1	-	-	1	-	-	-	-	-	-	-	-	-	-	-	1	-	-	-	-	-	-	-	-	-	-	-	-	-	-	-	-	-	-	-
Filobasidiella neoformans var. neoformans JEC21	Y	4	4	1	1	-	-	1	-	-	-	-	-	-	-	-	-	-	-	1	-	-	-	-	-	-	-	-	-	-	-	-	-	-	-	-	-	-	-
Filobasidiella neoformans var. neoformans JEC20	N	1	1	-	-	-	-	1	-	-	-	-	-	-	-	-	-	-	-	-	-	-	-	-	-	-	-	-	-	-	-	-	-	-	-	-	-	-	-
Coprinopsis cinerea okayama7#130	Y	4	4	1	1	-	-	1	-	-	-	-	-	-	-	-	-	-	-	1	-	-	-	-	-	-	-	-	-	-	-	-	-	-	-	-	-	-	-
Laccaria bicolor S238N	Y	4	7	1	1	-	-	1	-	-	-	-	-	-	-	-	-	-	-	4	-	-	-	-	-	-	-	-	-	-	-	-	-	-	-	-	-	-	-

				1	2	3	4	5	6	7	8	9	10	11	12	13	14	15	16	17	18	19	20	21	22	23	24	25	26	27	28	29	30	31	32	33	34	35	O
Phanerochaete chrysosporium RP-78	Y	4	5	1	1	-	-	1	-	-	-	-	-	-	-	-	-	-	-	2	-	-	-	-	-	-	-	-	-	-	-	-	-	-	-	-	-	-	-
Paxillus involutus	N	1	1	-	1	-	-	-	-	-	-	-	-	-	-	-	-	-	-	-	-	-	-	-	-	-	-	-	-	-	-	-	-	-	-	-	-	-	-
Sporobolomyces roseus IAM 13481	Y	4	5	1	1	-	-	1	-	-	-	-	-	-	-	-	-	-	-	2	-	-	-	-	-	-	-	-	-	-	-	-	-	-	-	-	-	-	-
Ustilago maydis 521	Y	4	4	1	1	-	-	1	-	-	-	-	-	-	-	-	-	-	-	1	-	-	-	-	-	-	-	-	-	-	-	-	-	-	-	-	-	-	-
Ustilago maydis FB1	Y	4	4	1	1	-	-	1	-	-	-	-	-	-	-	-	-	-	-	1	-	-	-	-	-	-	-	-	-	-	-	-	-	-	-	-	-	-	-

Chytridiomycota	Compl.	No. Class	No. Prot	1	2	3	4	5	6	7	8	9	10	11	12	13	14	15	16	17	18	19	20	21	22	23	24	25	26	27	28	29	30	31	32	33	34	35	O
Batrachochytrium dendrobatidis JEL423	Y	4	8	1	1	-	-	1	-	-	-	-	-	-	-	-	-	-	-	5	-	-	-	-	-	-	-	-	-	-	-	-	-	-	-	-	-	-	-

Microsporidia	Compl.	No. Class	No. Prot	1	2	3	4	5	6	7	8	9	10	11	12	13	14	15	16	17	18	19	20	21	22	23	24	25	26	27	28	29	30	31	32	33	34	35	O
Encephalitozoon cuniculi GB-M1	Y	1	2	-	1	-	-	-	-	-	-	-	-	-	-	-	-	-	-	-	-	-	-	-	-	-	-	-	-	-	-	-	-	-	-	-	-	-	1
Antonospora locustae	Y	1	2	-	1	-	-	-	-	-	-	-	-	-	-	-	-	-	-	-	-	-	-	-	-	-	-	-	-	-	-	-	-	-	-	-	-	-	1

Zygomycota	Compl.	No. Class	No. Prot	1	2	3	4	5	6	7	8	9	10	11	12	13	14	15	16	17	18	19	20	21	22	23	24	25	26	27	28	29	30	31	32	33	34	35	O
Phycomyces blakesleeanus	N	4	12	1	2	-	-	4	-	-	-	-	-	-	-	-	-	-	-	5	-	-	-	-	-	-	-	-	-	-	-	-	-	-	-	-	-	-	-
Rhizopus arrhizus RA 99-880	Y	4	19	1	2	-	-	7	-	-	-	-	-	-	-	-	-	-	-	9	-	-	-	-	-	-	-	-	-	-	-	-	-	-	-	-	-	-	-

Metazoa	Compl.	No. Class	No. Prot	1	2	3	4	5	6	7	8	9	10	11	12	13	14	15	16	17	18	19	20	21	22	23	24	25	26	27	28	29	30	31	32	33	34	35	O
Schistosoma mansoni	N	1	2	-	2	-	-	-	-	-	-	-	-	-	-	-	-	-	-	-	-	-	-	-	-	-	-	-	-	-	-	-	-	-	-	-	-	-	-
Schistosoma japonicum	N	1	3	-	3	-	-	-	-	-	-	-	-	-	-	-	-	-	-	-	-	-	-	-	-	-	-	-	-	-	-	-	-	-	-	-	-	-	-
Convoluta convoluta	N	1	1	-	1	-	-	-	-	-	-	-	-	-	-	-	-	-	-	-	-	-	-	-	-	-	-	-	-	-	-	-	-	-	-	-	-	-	-
Paratomella rubra	N	1	1	-	1	-	-	-	-	-	-	-	-	-	-	-	-	-	-	-	-	-	-	-	-	-	-	-	-	-	-	-	-	-	-	-	-	-	-
Symsagittifera roscofensis	N	1	1	-	1	-	-	-	-	-	-	-	-	-	-	-	-	-	-	-	-	-	-	-	-	-	-	-	-	-	-	-	-	-	-	-	-	-	-
Nemertoderma westbladi	N	1	1	-	1	-	-	-	-	-	-	-	-	-	-	-	-	-	-	-	-	-	-	-	-	-	-	-	-	-	-	-	-	-	-	-	-	-	-
Discocelis tigrina	N	1	1	-	1	-	-	-	-	-	-	-	-	-	-	-	-	-	-	-	-	-	-	-	-	-	-	-	-	-	-	-	-	-	-	-	-	-	-
Thysanozoon sp. IRT-2002	N	1	1	-	1	-	-	-	-	-	-	-	-	-	-	-	-	-	-	-	-	-	-	-	-	-	-	-	-	-	-	-	-	-	-	-	-	-	-
Dugesia japonica	N	1	2	-	2	-	-	-	-	-	-	-	-	-	-	-	-	-	-	-	-	-	-	-	-	-	-	-	-	-	-	-	-	-	-	-	-	-	-
Girardia tigrina	N	1	1	-	1	-	-	-	-	-	-	-	-	-	-	-	-	-	-	-	-	-	-	-	-	-	-	-	-	-	-	-	-	-	-	-	-	-	-
Schmidtea mediterranea	N	4	7	1	4	-	-	-	-	1	-	-	-	-	-	-	-	1	-	-	-	-	-	-	-	-	-	-	-	-	-	-	-	-	-	-	-	-	-
Erpobdellid sp. IRT-2002	N	1	1	-	1	-	-	-	-	-	-	-	-	-	-	-	-	-	-	-	-	-	-	-	-	-	-	-	-	-	-	-	-	-	-	-	-	-	-
Oligochaete sp. IRT-2002	N	1	1	-	1	-	-	-	-	-	-	-	-	-	-	-	-	-	-	-	-	-	-	-	-	-	-	-	-	-	-	-	-	-	-	-	-	-	-
Nereis sp. IRT-2002	N	1	1	-	1	-	-	-	-	-	-	-	-	-	-	-	-	-	-	-	-	-	-	-	-	-	-	-	-	-	-	-	-	-	-	-	-	-	-
Bonellia viridis	N	1	1	-	1	-	-	-	-	-	-	-	-	-	-	-	-	-	-	-	-	-	-	-	-	-	-	-	-	-	-	-	-	-	-	-	-	-	-
Phoronis hippocrepia	N	1	1	-	1	-	-	-	-	-	-	-	-	-	-	-	-	-	-	-	-	-	-	-	-	-	-	-	-	-	-	-	-	-	-	-	-	-	-
Brachiopod sp. IRT-2002	N	1	1	-	1	-	-	-	-	-	-	-	-	-	-	-	-	-	-	-	-	-	-	-	-	-	-	-	-	-	-	-	-	-	-	-	-	-	-
Lineus sp. IRT-2002	N	1	1	-	1	-	-	-	-	-	-	-	-	-	-	-	-	-	-	-	-	-	-	-	-	-	-	-	-	-	-	-	-	-	-	-	-	-	-
Priapulus caudatus	N	1	1	-	1	-	-	-	-	-	-	-	-	-	-	-	-	-	-	-	-	-	-	-	-	-	-	-	-	-	-	-	-	-	-	-	-	-	-
Phascolosoma granulatum	N	1	1	-	1	-	-	-	-	-	-	-	-	-	-	-	-	-	-	-	-	-	-	-	-	-	-	-	-	-	-	-	-	-	-	-	-	-	-
Brachionus plicatilis	N	1	1	-	1	-	-	-	-	-	-	-	-	-	-	-	-	-	-	-	-	-	-	-	-	-	-	-	-	-	-	-	-	-	-	-	-	-	-
Nematostella vectensis	Y	12	18	5	2	1	-	1	1	2	-	1	1	-	-	-	-	1	-	-	1	1	-	-	-	-	-	-	-	-	1	-	-	-	-	-	-	-	-
Anemonia sulcata	N	1	1	-	1	-	-	-	-	-	-	-	-	-	-	-	-	-	-	-	-	-	-	-	-	-	-	-	-	-	-	-	-	-	-	-	-	-	-
Bunodactis verrucosa	N	1	1	-	1	-	-	-	-	-	-	-	-	-	-	-	-	-	-	-	-	-	-	-	-	-	-	-	-	-	-	-	-	-	-	-	-	-	-
Podocoryne carnea	N	1	1	-	1	-	-	-	-	-	-	-	-	-	-	-	-	-	-	-	-	-	-	-	-	-	-	-	-	-	-	-	-	-	-	-	-	-	-
Hydra magnipapillata	N	1	2	2	-	-	-	-	-	-	-	-	-	-	-	-	-	-	-	-	-	-	-	-	-	-	-	-	-	-	-	-	-	-	-	-	-	-	-

Chordata	Compl.	No. Class	No. Prot	1	2	3	4	5	6	7	8	9	10	11	12	13	14	15	16	17	18	19	20	21	22	23	24	25	26	27	28	29	30	31	32	33	34	35	O
Lethenteron japonicum	N	1	2	-	2	-	-	-	-	-	-	-	-	-	-	-	-	-	-	-	-	-	-	-	-	-	-	-	-	-	-	-	-	-	-	-	-	-	-
Oikopleura dioica	N	1	1	-	1	-	-	-	-	-	-	-	-	-	-	-	-	-	-	-	-	-	-	-	-	-	-	-	-	-	-	-	-	-	-	-	-	-	-

Actinopterygii	Compl.	No. Class	No. Prot	1	2	3	4	5	6	7	8	9	10	11	12	13	14	15	16	17	18	19	20	21	22	23	24	25	26	27	28	29	30	31	32	33	34	35	O
Seriola dumerili	N	1	1	-	1	-	-	-	-	-	-	-	-	-	-	-	-	-	-	-	-	-	-	-	-	-	-	-	-	-	-	-	-	-	-	-	-	-	-
Ptyochromis sp. 'redtail sheller'	?	1	2	-	2	-	-	-	-	-	-	-	-	-	-	-	-	-	-	-	-	-	-	-	-	-	-	-	-	-	-	-	-	-	-	-	-	-	-
Notothenia coriiceps	N	1	2	-	2	-	-	-	-	-	-	-	-	-	-	-	-	-	-	-	-	-	-	-	-	-	-	-	-	-	-	-	-	-	-	-	-	-	-
Paracirrhites forsteri	N	1	1	-	1	-	-	-	-	-	-	-	-	-	-	-	-	-	-	-	-	-	-	-	-	-	-	-	-	-	-	-	-	-	-	-	-	-	-
Dicentrarchus labrax	N	1	1	-	1	-	-	-	-	-	-	-	-	-	-	-	-	-	-	-	-	-	-	-	-	-	-	-	-	-	-	-	-	-	-	-	-	-	-
Morone saxatilis	N	2	4	-	-	2	-	-	2	-	-	-	-	-	-	-	-	-	-	-	-	-	-	-	-	-	-	-	-	-	-	-	-	-	-	-	-	-	-
Pennahia argentata	N	1	1	-	1	-	-	-	-	-	-	-	-	-	-	-	-	-	-	-	-	-	-	-	-	-	-	-	-	-	-	-	-	-	-	-	-	-	-
Siniperca chuatsi	N	1	1	-	1	-	-	-	-	-	-	-	-	-	-	-	-	-	-	-	-	-	-	-	-	-	-	-	-	-	-	-	-	-	-	-	-	-	-
Oryzias latipes str. Hd-rR	Y	13	60	10	23	2	-	5	2	4	-	4	3	-	-	-	-	2	1	-	2	1	-	-	-	-	-	-	-	-	-	-	-	-	-	-	-	1	-
Fundulus heteroclitus	N	1	2	-	2	-	-	-	-	-	-	-	-	-	-	-	-	-	-	-	-	-	-	-	-	-	-	-	-	-	-	-	-	-	-	-	-	-	-
Gasterosteus aculeatus	Y	13	59	10	21	2	-	5	2	4	-	4	3	-	-	-	-	2	1	-	3	1	-	-	-	-	-	-	-	-	-	-	-	-	-	-	-	1	-
Takifugu rubripes	Y	13	59	10	21	2	-	5	2	4	-	4	3	-	-	-	-	2	1	-	3	1	-	-	-	-	-	-	-	-	-	-	-	-	-	-	-	1	-
Tetraodon nigroviridis	Y	13	60	10	22	2	-	5	2	4	-	4	3	-	-	-	-	2	1	-	3	1	-	-	-	-	-	-	-	-	-	-	-	-	-	-	-	1	-
Gadus morhua	N	1	2	-	2	-	-	-	-	-	-	-	-	-	-	-	-	-	-	-	-	-	-	-	-	-	-	-	-	-	-	-	-	-	-	-	-	-	-

Theragra chalcogramma	N	1	1	-	1	-	-	-	-	-	-	-	-	-	-	-	-	-	-	-	-	-	-	-	-	-	-	-	-	-	-	-	-	-	-	-	-	-	-
Oncorhynchus keta	N	1	1	-	1	-	-	-	-	-	-	-	-	-	-	-	-	-	-	-	-	-	-	-	-	-	-	-	-	-	-	-	-	-	-	-	-	-	-
Oncorhynchus mykiss	N	3	15	8	6	-	-	-	-	-	-	-	-	-	-	-	-	-	-	-	-	-	-	-	-	-	-	-	-	-	1	-	-	-	-	-	-	-	-
Salmo salar	N	3	6	2	3	-	-	-	-	-	-	-	-	-	-	-	-	-	-	-	-	-	-	-	-	-	-	-	-	-	1	-	-	-	-	-	-	-	-
Cyprinus carpio	N	1	9	-	9	-	-	-	-	-	-	-	-	-	-	-	-	-	-	-	-	-	-	-	-	-	-	-	-	-	-	-	-	-	-	-	-	-	-
Pimephales promelas	N	2	2	-	-	-	-	-	-	-	-	-	-	-	-	-	-	-	-	-	-	-	1	-	-	-	-	-	-	-	-	1	-	-	-	-	-	-	-
Brachydanio rerio	Y	14	61	10	22	2	-	5	2	4	-	4	3	-	-	-	-	2	1	-	3	1	-	-	-	-	-	-	-	-	1	-	-	-	-	-	-	1	-
Ictalurus punctatus	N	4	5	2	-	-	-	-	-	-	-	-	1	-	-	-	-	1	-	-	-	-	-	-	-	-	-	-	-	-	1	-	-	-	-	-	-	-	-
Mammalia	Compl.	No. Class	No. Prot	1	2	3	4	5	6	7	8	9	10	11	12	13	14	15	16	17	18	19	20	21	22	23	24	25	26	27	28	29	30	31	32	33	34	35	O
Ornithorhynchus anatinus	N	12	26	4	7	2	-	3	1	-	-	2	1	-	-	-	-	1	1	-	2	1	-	-	-	-	-	-	-	-	-	-	-	-	-	-	-	1	-
Echinops telfairi	N	1	1	-	-	-	-	-	-	-	-	-	-	-	-	-	-	-	-	-	-	1	-	-	-	-	-	-	-	-	-	-	-	-	-	-	-	-	-
Oryctolagus cuniculus	N	1	8	-	8	-	-	-	-	-	-	-	-	-	-	-	-	-	-	-	-	-	-	-	-	-	-	-	-	-	-	-	-	-	-	-	-	-	-
Canis familiaris	Y	13	40	8	15	2	-	3	1	2	-	2	1	-	-	-	-	1	1	-	2	1	-	-	-	-	-	-	-	-	-	-	-	-	-	-	-	1	-
Felis catus	N	1	3	-	3	-	-	-	-	-	-	-	-	-	-	-	-	-	-	-	-	-	-	-	-	-	-	-	-	-	-	-	-	-	-	-	-	-	-
Bos taurus	Y	13	39	8	14	2	-	3	1	2	-	2	1	-	-	-	-	1	1	-	2	1	-	-	-	-	-	-	-	-	-	-	-	-	-	-	-	1	-
Sus scrofa domestica	N	7	18	7	6	-	-	1	1	1	-	-	-	-	-	-	-	-	-	-	1	1	-	-	-	-	-	-	-	-	-	-	-	-	-	-	-	-	-
Carollia perspicillata	Y	1	1	-	1	-	-	-	-	-	-	-	-	-	-	-	-	-	-	-	-	-	-	-	-	-	-	-	-	-	-	-	-	-	-	-	-	-	-
Myotis lucifugus	N	2	2	1	1	-	-	-	-	-	-	-	-	-	-	-	-	-	-	-	-	-	-	-	-	-	-	-	-	-	-	-	-	-	-	-	-	-	-
Equus caballus	N	1	3	-	3	-	-	-	-	-	-	-	-	-	-	-	-	-	-	-	-	-	-	-	-	-	-	-	-	-	-	-	-	-	-	-	-	-	-
Dasypus novemcinctus	N	1	1	-	1	-	-	-	-	-	-	-	-	-	-	-	-	-	-	-	-	-	-	-	-	-	-	-	-	-	-	-	-	-	-	-	-	-	-
Monodelphis domestica	Y	12	39	8	15	2	-	3	1	2	-	2	1	-	-	-	-	1	1	-	2	1	-	-	-	-	-	-	-	-	-	-	-	-	-	-	-	-	-
Trichosurus vulpecula	N	1	1	-	-	-	-	-	-	-	-	-	-	-	-	-	-	-	-	-	-	1	-	-	-	-	-	-	-	-	-	-	-	-	-	-	-	-	-
Rodentia	Compl.	No. Class	No. Prot	1	2	3	4	5	6	7	8	9	10	11	12	13	14	15	16	17	18	19	20	21	22	23	24	25	26	27	28	29	30	31	32	33	34	35	O
Mesocricetus auratus	N	1	2	-	2	-	-	-	-	-	-	-	-	-	-	-	-	-	-	-	-	-	-	-	-	-	-	-	-	-	-	-	-	-	-	-	-	-	-
Mus musculus	Y	13	39	8	14	2	-	3	1	2	-	2	1	-	-	-	-	1	1	-	2	1	-	-	-	-	-	-	-	-	-	-	-	-	-	-	-	1	-
Rattus norvegicus	Y	13	39	8	14	2	-	3	1	2	-	2	1	-	-	-	-	1	1	-	2	1	-	-	-	-	-	-	-	-	-	-	-	-	-	-	-	1	-
Spermophilus tridecemlineatus	N	1	1	-	-	-	-	-	-	-	-	-	-	-	-	-	-	-	-	-	-	1	-	-	-	-	-	-	-	-	-	-	-	-	-	-	-	-	-
Primates	Compl.	No. Class	No. Prot	1	2	3	4	5	6	7	8	9	10	11	12	13	14	15	16	17	18	19	20	21	22	23	24	25	26	27	28	29	30	31	32	33	34	35	O
Macaca mulatta	N	13	40	8	15	2	-	3	1	2	-	2	1	-	-	-	-	1	1	-	2	1	-	-	-	-	-	-	-	-	-	-	-	-	-	-	-	1	-
Macaca fascicularis	N	2	3	2	-	-	-	-	-	-	-	1	-	-	-	-	-	-	-	-	-	-	-	-	-	-	-	-	-	-	-	-	-	-	-	-	-	-	-
Homo sapiens	Y	13	40	8	15	2	-	3	1	2	-	2	1	-	-	-	-	1	1	-	2	1	-	-	-	-	-	-	-	-	-	-	-	-	-	-	-	1	-
Pan troglodytes	Y	13	40	8	15	2	-	3	1	2	-	2	1	-	-	-	-	1	1	-	2	1	-	-	-	-	-	-	-	-	-	-	-	-	-	-	-	1	-
Pongo pygmaeus	N	2	3	1	2	-	-	-	-	-	-	-	-	-	-	-	-	-	-	-	-	-	-	-	-	-	-	-	-	-	-	-	-	-	-	-	-	-	-
Otolemur garnettii	?	1	1	-	1	-	-	-	-	-	-	-	-	-	-	-	-	-	-	-	-	-	-	-	-	-	-	-	-	-	-	-	-	-	-	-	-	-	-
Aves	Compl.	No. Class	No. Prot	1	2	3	4	5	6	7	8	9	10	11	12	13	14	15	16	17	18	19	20	21	22	23	24	25	26	27	28	29	30	31	32	33	34	35	O
Coturnix coturnix	N	1	2	-	2	-	-	-	-	-	-	-	-	-	-	-	-	-	-	-	-	-	-	-	-	-	-	-	-	-	-	-	-	-	-	-	-	-	-
Gallus gallus	Y	13	41	8	15	2	-	3	1	2	-	2	2	-	-	-	-	1	1	-	2	1	-	-	-	-	-	-	-	-	1	-	-	-	-	-	-	-	-
Amphibia	Compl.	No. Class	No. Prot	1	2	3	4	5	6	7	8	9	10	11	12	13	14	15	16	17	18	19	20	21	22	23	24	25	26	27	28	29	30	31	32	33	34	35	O
Xenopus tropicalis	Y	14	47	8	20	2	-	3	1	2	-	2	2	-	-	-	-	1	1	-	2	1	-	-	-	-	-	-	-	-	1	-	-	-	-	-	-	1	-
Xenopus laevis	N	7	23	6	11	-	-	1	1	-	-	1	2	-	-	-	-	-	-	-	-	1	-	-	-	-	-	-	-	-	-	-	-	-	-	-	-	-	-
Rana catesbeiana	N	6	10	2	4	-	-	1	1	1	-	-	1	-	-	-	-	-	-	-	-	-	-	-	-	-	-	-	-	-	-	-	-	-	-	-	-	-	-
Rana pipiens	N	1	4	-	4	-	-	-	-	-	-	-	-	-	-	-	-	-	-	-	-	-	-	-	-	-	-	-	-	-	-	-	-	-	-	-	-	-	-
Ascidiacea	Compl.	No. Class	No. Prot	1	2	3	4	5	6	7	8	9	10	11	12	13	14	15	16	17	18	19	20	21	22	23	24	25	26	27	28	29	30	31	32	33	34	35	O
Ciona intestinalis	Y	9	17	4	6	-	-	1	1	1	-	1	1	-	-	-	-	-	-	-	1	1	-	-	-	-	-	-	-	-	-	-	-	-	-	-	-	-	-
Ciona savignyi	Y	9	17	4	6	-	-	1	1	1	-	1	1	-	-	-	-	-	-	-	1	1	-	-	-	-	-	-	-	-	-	-	-	-	-	-	-	-	-
Halocynthia roretzi	N	1	1	-	1	-	-	-	-	-	-	-	-	-	-	-	-	-	-	-	-	-	-	-	-	-	-	-	-	-	-	-	-	-	-	-	-	-	-
Echinodermata	Compl.	No. Class	No. Prot	1	2	3	4	5	6	7	8	9	10	11	12	13	14	15	16	17	18	19	20	21	22	23	24	25	26	27	28	29	30	31	32	33	34	35	O
Strongylocentrotus purpuratus	N	12	18	3	2	-	-	1	1	1	-	2	1	-	-	-	-	1	-	-	1	1	1	-	-	-	-	-	-	-	1	-	-	-	-	-	-	-	2
Mollusca	Compl.	No. Class	No. Prot	1	2	3	4	5	6	7	8	9	10	11	12	13	14	15	16	17	18	19	20	21	22	23	24	25	26	27	28	29	30	31	32	33	34	35	O
Spisula solidissima	N	1	1	-	1	-	-	-	-	-	-	-	-	-	-	-	-	-	-	-	-	-	-	-	-	-	-	-	-	-	-	-	-	-	-	-	-	-	-
Mytilus galloprovincialis	N	1	2	-	2	-	-	-	-	-	-	-	-	-	-	-	-	-	-	-	-	-	-	-	-	-	-	-	-	-	-	-	-	-	-	-	-	-	-
Argopecten irradians	N	1	2	-	2	-	-	-	-	-	-	-	-	-	-	-	-	-	-	-	-	-	-	-	-	-	-	-	-	-	-	-	-	-	-	-	-	-	-
Chlamys farreri	N	1	1	-	-	-	-	-	1	-	-	-	-	-	-	-	-	-	-	-	-	-	-	-	-	-	-	-	-	-	-	-	-	-	-	-	-	-	-
Mizuhopecten yessoensis	N	3	5	-	2	-	-	-	-	1	-	-	-	-	-	-	-	-	-	-	-	-	-	-	1	-	-	-	-	-	-	-	-	-	-	-	-	-	1
Pecten maximus	N	1	1	-	1	-	-	-	-	-	-	-	-	-	-	-	-	-	-	-	-	-	-	-	-	-	-	-	-	-	-	-	-	-	-	-	-	-	-
Placopecten magellanicus	N	1	1	-	1	-	-	-	-	-	-	-	-	-	-	-	-	-	-	-	-	-	-	-	-	-	-	-	-	-	-	-	-	-	-	-	-	-	-
Atrina rigida	N	0	1	-	-	-	-	-	-	-	-	-	-	-	-	-	-	-	-	-	-	-	-	-	-	-	-	-	-	-	-	-	-	-	-	-	-	-	1
Loligo pealeii	N	2	4	-	3	-	-	1	-	-	-	-	-	-	-	-	-	-	-	-	-	-	-	-	-	-	-	-	-	-	-	-	-	-	-	-	-	-	-
Ilyanassa obsoleta	N	1	1	-	1	-	-	-	-	-	-	-	-	-	-	-	-	-	-	-	-	-	-	-	-	-	-	-	-	-	-	-	-	-	-	-	-	-	-
Aplysia californica	N	1	1	-	1	-	-	-	-	-	-	-	-	-	-	-	-	-	-	-	-	-	-	-	-	-	-	-	-	-	-	-	-	-	-	-	-	-	-
Arthropoda	Compl.	No. Class	No. Prot	1	2	3	4	5	6	7	8	9	10	11	12	13	14	15	16	17	18	19	20	21	22	23	24	25	26	27	28	29	30	31	32	33	34	35	O
Rhipicephalus appendiculatus	N	3	4	1	2	-	-	-	1	-	-	-	-	-	-	-	-	-	-	-	-	-	-	-	-	-	-	-	-	-	-	-	-	-	-	-	-	-	-
Cyrtophora citricola	N	1	1	-	1	-	-	-	-	-	-	-	-	-	-	-	-	-	-	-	-	-	-	-	-	-	-	-	-	-	-	-	-	-	-	-	-	-	-

				1	2	3	4	5	6	7	8	9	10	11	12	13	14	15	16	17	18	19	20	21	22	23	24	25	26	27	28	29	30	31	32	33	34	35	O
Opiliones sp. IRT-2002	N	1	1	-	1	-	-	-	-	-	-	-	-	-	-	-	-	-	-	-	-	-	-	-	-	-	-	-	-	-	-	-	-	-	-	-	-	-	-
Euscorpius flavicaudis	N	1	1	-	1	-	-	-	-	-	-	-	-	-	-	-	-	-	-	-	-	-	-	-	-	-	-	-	-	-	-	-	-	-	-	-	-	-	-
Limulus polyphemus	N	1	1	-	-	-	-	-	-	-	-	-	-	-	-	-	-	-	-	-	-	-	-	1	-	-	-	-	-	-	-	-	-	-	-	-	-	-	-
Scutigera coleoptrata	N	1	1	-	1	-	-	-	-	-	-	-	-	-	-	-	-	-	-	-	-	-	-	-	-	-	-	-	-	-	-	-	-	-	-	-	-	-	-
Lithobius sp. IRT-2002	N	1	1	-	1	-	-	-	-	-	-	-	-	-	-	-	-	-	-	-	-	-	-	-	-	-	-	-	-	-	-	-	-	-	-	-	-	-	-
Daphnia pulex	Y	13	17	4	2	1	-	1	1	1	-	1	-	-	-	-	-	1	-	-	1	1	1	1	1	-	-	-	-	-	-	-	-	-	-	-	-	-	-
Homarus gammarus	N	1	3	-	3	-	-	-	-	-	-	-	-	-	-	-	-	-	-	-	-	-	-	-	-	-	-	-	-	-	-	-	-	-	-	-	-	-	-
Idotea resecata	N	1	1	-	1	-	-	-	-	-	-	-	-	-	-	-	-	-	-	-	-	-	-	-	-	-	-	-	-	-	-	-	-	-	-	-	-	-	-
Insecta	Compl.	No. Class	No. Prot	1	2	3	4	5	6	7	8	9	10	11	12	13	14	15	16	17	18	19	20	21	22	23	24	25	26	27	28	29	30	31	32	33	34	35	O
Bombyx mori str. Dazao	N	12	15	2	2	1	-	1	1	2	-	1	-	-	-	-	-	1	-	-	1	-	1	1	1	-	-	-	-	-	-	-	-	-	-	-	-	-	-
Tribolium castaneum str. Georgia GA2	Y	11	15	3	2	-	-	1	1	2	-	1	-	-	-	-	-	1	-	-	1	-	1	1	1	-	-	-	-	-	-	-	-	-	-	-	-	-	-
Apis mellifera str. DH4	Y	12	14	2	2	1	-	1	1	1	-	1	-	-	-	-	-	1	-	-	1	-	1	1	1	-	-	-	-	-	-	-	-	-	-	-	-	-	-
Ammophila sp. IRT-2002	N	1	1	-	1	-	-	-	-	-	-	-	-	-	-	-	-	-	-	-	-	-	-	-	-	-	-	-	-	-	-	-	-	-	-	-	-	-	-
Nasonia vitripennis	Y	11	15	2	2	1	-	1	-	2	-	1	-	-	-	-	-	1	-	-	1	-	1	1	1	-	-	-	-	-	-	-	-	-	-	-	-	-	1
Blatta sp. IRT-2002	N	1	1	-	1	-	-	-	-	-	-	-	-	-	-	-	-	-	-	-	-	-	-	-	-	-	-	-	-	-	-	-	-	-	-	-	-	-	-
Empusa sp. IRT-2002	N	1	1	-	1	-	-	-	-	-	-	-	-	-	-	-	-	-	-	-	-	-	-	-	-	-	-	-	-	-	-	-	-	-	-	-	-	-	-
Lepisma saccharina	N	1	1	-	1	-	-	-	-	-	-	-	-	-	-	-	-	-	-	-	-	-	-	-	-	-	-	-	-	-	-	-	-	-	-	-	-	-	-
Diptera	Compl.	No. Class	No. Prot	1	2	3	4	5	6	7	8	9	10	11	12	13	14	15	16	17	18	19	20	21	22	23	24	25	26	27	28	29	30	31	32	33	34	35	O
Drosophila grimshawi TSC#15287-2541.00	Y	10	14	3	2	-	-	1	1	2	-	-	-	-	-	-	-	1	-	-	1	-	1	1	1	-	-	-	-	-	-	-	-	-	-	-	-	-	-
Drosophila hydei	N	1	1	-	1	-	-	-	-	-	-	-	-	-	-	-	-	-	-	-	-	-	-	-	-	-	-	-	-	-	-	-	-	-	-	-	-	-	-
Drosophila mojavensis TSC#15081-1352.22	Y	9	13	3	2	-	-	1	1	2	-	-	-	-	-	-	-	1	-	-	1	-	1	1	-	-	-	-	-	-	-	-	-	-	-	-	-	-	-
Drosophila virilis TSC#15010-1051.87	Y	9	13	3	2	-	-	1	1	2	-	-	-	-	-	-	-	1	-	-	1	-	1	1	-	-	-	-	-	-	-	-	-	-	-	-	-	-	-
Drosophila ananassae TSC#14024-0371.13	Y	9	13	3	2	-	-	1	1	2	-	-	-	-	-	-	-	1	-	-	1	-	1	1	-	-	-	-	-	-	-	-	-	-	-	-	-	-	-
Drosophila yakuba Tai18E2	Y	9	13	3	2	-	-	1	1	2	-	-	-	-	-	-	-	1	-	-	1	-	1	1	-	-	-	-	-	-	-	-	-	-	-	-	-	-	-
Drosophila melanogaster	Y	9	13	3	2	-	-	1	1	2	-	-	-	-	-	-	-	1	-	-	1	-	1	1	-	-	-	-	-	-	-	-	-	-	-	-	-	-	-
Drosophila erecta TSC#14021-0224.01	Y	9	13	3	2	-	-	1	1	2	-	-	-	-	-	-	-	1	-	-	1	-	1	1	-	-	-	-	-	-	-	-	-	-	-	-	-	-	-
Drosophila sechellia Rob3c	Y	9	13	3	2	-	-	1	1	2	-	-	-	-	-	-	-	1	-	-	1	-	1	1	-	-	-	-	-	-	-	-	-	-	-	-	-	-	-
Drosophila simulans str. white501	Y	9	13	3	2	-	-	1	1	2	-	-	-	-	-	-	-	1	-	-	1	-	1	1	-	-	-	-	-	-	-	-	-	-	-	-	-	-	-
Drosophila persimilis MSH-3	Y	10	14	3	2	-	-	1	1	2	-	-	-	-	-	-	-	1	-	-	1	-	1	1	1	-	-	-	-	-	-	-	-	-	-	-	-	-	-
Drosophila pseudoobscura MV2-25	Y	10	14	3	2	-	-	1	1	2	-	-	-	-	-	-	-	1	-	-	1	-	1	1	1	-	-	-	-	-	-	-	-	-	-	-	-	-	-
Drosophila willistoni TSC#14030-0811.24	Y	10	14	3	2	-	-	1	1	2	-	-	-	-	-	-	-	1	-	-	1	-	1	1	1	-	-	-	-	-	-	-	-	-	-	-	-	-	-
Glossina morsitans morsitans	N	3	4	2	1	-	-	-	-	-	-	-	-	-	-	-	-	-	-	-	-	-	-	-	1	-	-	-	-	-	-	-	-	-	-	-	-	-	-
Anopheles gambiae str. PEST	Y	12	16	3	2	1	-	1	1	2	-	1	-	-	-	-	-	1	-	-	1	-	1	1	1	-	-	-	-	-	-	-	-	-	-	-	-	-	-
Aedes aegypti str. Liverpool	Y	12	17	3	3	1	-	1	1	2	-	1	-	-	-	-	-	1	-	-	1	-	1	1	1	-	-	-	-	-	-	-	-	-	-	-	-	-	-
Armigeres subalbatus	N	1	1	-	1	-	-	-	-	-	-	-	-	-	-	-	-	-	-	-	-	-	-	-	-	-	-	-	-	-	-	-	-	-	-	-	-	-	-
Nematoda	Compl.	No. Class	No. Prot	1	2	3	4	5	6	7	8	9	10	11	12	13	14	15	16	17	18	19	20	21	22	23	24	25	26	27	28	29	30	31	32	33	34	35	O
Ascaris suum	N	1	2	-	2	-	-	-	-	-	-	-	-	-	-	-	-	-	-	-	-	-	-	-	-	-	-	-	-	-	-	-	-	-	-	-	-	-	-
Toxocara canis	N	1	2	-	2	-	-	-	-	-	-	-	-	-	-	-	-	-	-	-	-	-	-	-	-	-	-	-	-	-	-	-	-	-	-	-	-	-	-
Brugia malayi	N	8	14	1	6	-	-	1	2	1	-	1	-	-	1	-	-	-	-	-	1	-	-	-	-	-	-	-	-	-	-	-	-	-	-	-	-	-	-
Onchocerca volvulus	N	1	2	-	2	-	-	-	-	-	-	-	-	-	-	-	-	-	-	-	-	-	-	-	-	-	-	-	-	-	-	-	-	-	-	-	-	-	-
Meloidogyne incognita	N	1	1	-	1	-	-	-	-	-	-	-	-	-	-	-	-	-	-	-	-	-	-	-	-	-	-	-	-	-	-	-	-	-	-	-	-	-	-
Meloidogyne arenaria	N	1	1	1	-	-	-	-	-	-	-	-	-	-	-	-	-	-	-	-	-	-	-	-	-	-	-	-	-	-	-	-	-	-	-	-	-	-	-
Meloidogyne chitwoodi	N	1	1	-	1	-	-	-	-	-	-	-	-	-	-	-	-	-	-	-	-	-	-	-	-	-	-	-	-	-	-	-	-	-	-	-	-	-	-
Xiphinema index	N	1	1	1	-	-	-	-	-	-	-	-	-	-	-	-	-	-	-	-	-	-	-	-	-	-	-	-	-	-	-	-	-	-	-	-	-	-	-
Trichinella spiralis	N	5	7	1	3	-	-	1	-	1	-	-	-	-	1	-	-	-	-	-	-	-	-	-	-	-	-	-	-	-	-	-	-	-	-	-	-	-	-
Rhabditida	Compl.	No. Class	No. Prot	1	2	3	4	5	6	7	8	9	10	11	12	13	14	15	16	17	18	19	20	21	22	23	24	25	26	27	28	29	30	31	32	33	34	35	O
Strongyloides ratti	N	1	1	-	1	-	-	-	-	-	-	-	-	-	-	-	-	-	-	-	-	-	-	-	-	-	-	-	-	-	-	-	-	-	-	-	-	-	-
Strongyloides stercoralis	N	2	5	1	4	-	-	-	-	-	-	-	-	-	-	-	-	-	-	-	-	-	-	-	-	-	-	-	-	-	-	-	-	-	-	-	-	-	-
Caenorhabditis remanei PB4641	Y	7	17	2	9	-	-	1	2	1	-	1	-	-	1	-	-	-	-	-	-	-	-	-	-	-	-	-	-	-	-	-	-	-	-	-	-	-	-
Caenorhabditis elegans	Y	7	17	2	9	-	-	1	2	1	-	1	-	-	1	-	-	-	-	-	-	-	-	-	-	-	-	-	-	-	-	-	-	-	-	-	-	-	-
Caenorhabditis briggsae	Y	7	17	2	9	-	-	1	2	1	-	1	-	-	1	-	-	-	-	-	-	-	-	-	-	-	-	-	-	-	-	-	-	-	-	-	-	-	-
Haemonchus contortus	N	1	3	-	3	-	-	-	-	-	-	-	-	-	-	-	-	-	-	-	-	-	-	-	-	-	-	-	-	-	-	-	-	-	-	-	-	-	-
Ostertagia ostertagi	N	1	1	-	1	-	-	-	-	-	-	-	-	-	-	-	-	-	-	-	-	-	-	-	-	-	-	-	-	-	-	-	-	-	-	-	-	-	-
Teladorsagia circumcincta	N	1	1	-	-	-	-	-	-	-	-	-	-	-	1	-	-	-	-	-	-	-	-	-	-	-	-	-	-	-	-	-	-	-	-	-	-	-	-
Dictyosteliida	Compl.	No. Class	No. Prot	1	2	3	4	5	6	7	8	9	10	11	12	13	14	15	16	17	18	19	20	21	22	23	24	25	26	27	28	29	30	31	32	33	34	35	O
Dictyostelium discoideum AX4	Y	3	13	7	1	-	-	2	-	-	-	-	-	-	-	-	-	-	-	-	-	-	-	-	-	-	-	-	-	-	-	-	-	-	-	-	-	-	3

Rhodophyta	Compl.	No. Class	No. Prot	1	2	3	4	5	6	7	8	9	10	11	12	13	14	15	16	17	18	19	20	21	22	23	24	25	26	27	28	29	30	31	32	33	34	35	O
Galdieria sulphuraria	Y	0	1	-	-	-	-	-	-	-	-	-	-	-	-	-	-	-	-	-	-	-	-	-	-	-	-	-	-	-	-	-	-	-	-	-	-	-	1

Viridiplantae	Compl.	No. Class	No. Prot	1	2	3	4	5	6	7	8	9	10	11	12	13	14	15	16	17	18	19	20	21	22	23	24	25	26	27	28	29	30	31	32	33	34	35	O
Chlamydomonas reinhardtii	Y	2	3	-	-	-	-	-	-	-	1	-	-	2	-	-	-	-	-	-	-	-	-	-	-	-	-	-	-	-	-	-	-	-	-	-	-	-	-
Ostreococcus lucimarinus	Y	1	1	-	-	-	-	-	-	-	-	-	-	1	-	-	-	-	-	-	-	-	-	-	-	-	-	-	-	-	-	-	-	-	-	-	-	-	-
Ostreococcus tauri	Y	1	1	-	-	-	-	-	-	-	-	-	-	1	-	-	-	-	-	-	-	-	-	-	-	-	-	-	-	-	-	-	-	-	-	-	-	-	-
Acetabularia peniculus	N	1	2	-	-	-	-	-	-	-	-	-	-	2	-	-	-	-	-	-	-	-	-	-	-	-	-	-	-	-	-	-	-	-	-	-	-	-	-

Streptophyta	Compl.	No. Class	No. Prot	1	2	3	4	5	6	7	8	9	10	11	12	13	14	15	16	17	18	19	20	21	22	23	24	25	26	27	28	29	30	31	32	33	34	35	O
Chara corallina	N	1	1	-	-	-	-	-	-	-	-	-	-	1	-	-	-	-	-	-	-	-	-	-	-	-	-	-	-	-	-	-	-	-	-	-	-	-	-
Adiantum capillus-veneris	N	1	2	-	-	-	-	-	-	-	-	-	-	2	-	-	-	-	-	-	-	-	-	-	-	-	-	-	-	-	-	-	-	-	-	-	-	-	-
Anemia phyllitidis	N	2	2	-	-	-	-	-	-	-	1	-	-	1	-	-	-	-	-	-	-	-	-	-	-	-	-	-	-	-	-	-	-	-	-	-	-	-	-
Pinus taeda	N	1	1	-	-	-	-	-	-	-	-	-	-	1	-	-	-	-	-	-	-	-	-	-	-	-	-	-	-	-	-	-	-	-	-	-	-	-	-
Petroselinum crispum	N	2	2	-	-	-	-	-	-	-	1	-	-	1	-	-	-	-	-	-	-	-	-	-	-	-	-	-	-	-	-	-	-	-	-	-	-	-	-
Helianthus annuus	N	2	5	-	-	-	-	-	-	-	2	-	-	3	-	-	-	-	-	-	-	-	-	-	-	-	-	-	-	-	-	-	-	-	-	-	-	-	-
Lactuca sativa	N	2	2	-	-	-	-	-	-	-	1	-	-	1	-	-	-	-	-	-	-	-	-	-	-	-	-	-	-	-	-	-	-	-	-	-	-	-	-
Capsicum annuum	N	1	1	-	-	-	-	-	-	-	1	-	-	-	-	-	-	-	-	-	-	-	-	-	-	-	-	-	-	-	-	-	-	-	-	-	-	-	-
Nicotiana tabacum	N	1	2	-	-	-	-	-	-	-	-	-	-	2	-	-	-	-	-	-	-	-	-	-	-	-	-	-	-	-	-	-	-	-	-	-	-	-	-
Solanum tuberosum	N	1	2	-	-	-	-	-	-	-	-	-	-	2	-	-	-	-	-	-	-	-	-	-	-	-	-	-	-	-	-	-	-	-	-	-	-	-	-
Solanum habrochaites	N	1	2	-	-	-	-	-	-	-	-	-	-	2	-	-	-	-	-	-	-	-	-	-	-	-	-	-	-	-	-	-	-	-	-	-	-	-	-
Lycopersicon esculentum	N	1	1	-	-	-	-	-	-	-	-	-	-	1	-	-	-	-	-	-	-	-	-	-	-	-	-	-	-	-	-	-	-	-	-	-	-	-	-
Beta vulgaris	N	1	1	-	-	-	-	-	-	-	-	-	-	1	-	-	-	-	-	-	-	-	-	-	-	-	-	-	-	-	-	-	-	-	-	-	-	-	-
Lotus japonicus	N	2	9	-	-	-	-	-	-	-	3	-	-	6	-	-	-	-	-	-	-	-	-	-	-	-	-	-	-	-	-	-	-	-	-	-	-	-	-
Glycine max	N	2	5	-	-	-	-	-	-	-	3	-	-	2	-	-	-	-	-	-	-	-	-	-	-	-	-	-	-	-	-	-	-	-	-	-	-	-	-
Phaseolus vulgaris	N	2	2	-	-	-	-	-	-	-	1	-	-	1	-	-	-	-	-	-	-	-	-	-	-	-	-	-	-	-	-	-	-	-	-	-	-	-	-
Medicago truncatula	N	2	12	-	-	-	-	-	-	-	4	-	-	8	-	-	-	-	-	-	-	-	-	-	-	-	-	-	-	-	-	-	-	-	-	-	-	-	-
Ricinus communis	N	2	9	-	-	-	-	-	-	-	2	-	-	7	-	-	-	-	-	-	-	-	-	-	-	-	-	-	-	-	-	-	-	-	-	-	-	-	-
Populus trichocarpa	Y	2	14	-	-	-	-	-	-	-	4	-	-	10	-	-	-	-	-	-	-	-	-	-	-	-	-	-	-	-	-	-	-	-	-	-	-	-	-
Arabidopsis thaliana	Y	2	17	-	-	-	-	-	-	-	4	-	-	13	-	-	-	-	-	-	-	-	-	-	-	-	-	-	-	-	-	-	-	-	-	-	-	-	-
Brassica napus	N	1	1	-	-	-	-	-	-	-	1	-	-	-	-	-	-	-	-	-	-	-	-	-	-	-	-	-	-	-	-	-	-	-	-	-	-	-	-
Brassica rapa subsp. pekinensis	N	1	6	-	-	-	-	-	-	-	-	-	-	6	-	-	-	-	-	-	-	-	-	-	-	-	-	-	-	-	-	-	-	-	-	-	-	-	-
Gossypium hirsutum	N	1	1	-	-	-	-	-	-	-	1	-	-	-	-	-	-	-	-	-	-	-	-	-	-	-	-	-	-	-	-	-	-	-	-	-	-	-	-
Gossypium raimondii	N	1	2	-	-	-	-	-	-	-	2	-	-	-	-	-	-	-	-	-	-	-	-	-	-	-	-	-	-	-	-	-	-	-	-	-	-	-	-
Vitis vinifera	N	2	2	-	-	-	-	-	-	-	1	-	-	1	-	-	-	-	-	-	-	-	-	-	-	-	-	-	-	-	-	-	-	-	-	-	-	-	-
Aquilegia	N	1	2	-	-	-	-	-	-	-	2	-	-	-	-	-	-	-	-	-	-	-	-	-	-	-	-	-	-	-	-	-	-	-	-	-	-	-	-
Vallisneria gigantea	N	2	2	-	-	-	-	-	-	-	1	-	-	1	-	-	-	-	-	-	-	-	-	-	-	-	-	-	-	-	-	-	-	-	-	-	-	-	-
Allium cepa	N	1	1	-	-	-	-	-	-	-	1	-	-	-	-	-	-	-	-	-	-	-	-	-	-	-	-	-	-	-	-	-	-	-	-	-	-	-	-
Oryza sativa (indica cultivar-group)	Y	2	13	-	-	-	-	-	-	-	2	-	-	11	-	-	-	-	-	-	-	-	-	-	-	-	-	-	-	-	-	-	-	-	-	-	-	-	-
Oryza sativa (japonica cultivar-group)	Y	2	13	-	-	-	-	-	-	-	2	-	-	11	-	-	-	-	-	-	-	-	-	-	-	-	-	-	-	-	-	-	-	-	-	-	-	-	-
Saccharum officinarum	N	2	6	-	-	-	-	-	-	-	3	-	-	3	-	-	-	-	-	-	-	-	-	-	-	-	-	-	-	-	-	-	-	-	-	-	-	-	-
Sorghum bicolor	Y	2	12	-	-	-	-	-	-	-	2	-	-	10	-	-	-	-	-	-	-	-	-	-	-	-	-	-	-	-	-	-	-	-	-	-	-	-	-
Zea mays	N	2	7	-	-	-	-	-	-	-	1	-	-	6	-	-	-	-	-	-	-	-	-	-	-	-	-	-	-	-	-	-	-	-	-	-	-	-	-
Hordeum vulgare subsp. vulgare	N	2	5	-	-	-	-	-	-	-	2	-	-	3	-	-	-	-	-	-	-	-	-	-	-	-	-	-	-	-	-	-	-	-	-	-	-	-	-
Triticum aestivum	N	2	8	-	-	-	-	-	-	-	4	-	-	4	-	-	-	-	-	-	-	-	-	-	-	-	-	-	-	-	-	-	-	-	-	-	-	-	-

stramenopiles	Compl.	No. Class	No. Prot	1	2	3	4	5	6	7	8	9	10	11	12	13	14	15	16	17	18	19	20	21	22	23	24	25	26	27	28	29	30	31	32	33	34	35	O
Phaeodactylum tricornutum CCAP1055/1	Y	1	10	-	-	-	-	-	-	-	-	-	-	-	-	-	-	-	-	-	-	-	-	-	-	-	-	-	-	-	-	1	-	-	-	-	-	-	9
Thalassiosira pseudonana CCMP1335	Y	1	11	-	-	-	-	-	-	-	-	-	-	-	-	-	-	-	-	-	-	-	-	-	-	-	-	-	-	-	-	1	-	-	-	-	-	-	10
Hyaloperonospora parasitica	Y	6	22	2	-	-	-	-	-	-	-	-	-	-	-	-	-	-	-	-	-	-	-	-	-	-	-	-	-	-	-	1	3	1	1	-	1	-	13
Phytophthora parasitica	N	1	1	1	-	-	-	-	-	-	-	-	-	-	-	-	-	-	-	-	-	-	-	-	-	-	-	-	-	-	-	-	-	-	-	-	-	-	-
Phytophthora ramorum Pr102	Y	7	24	2	-	-	-	-	-	-	-	-	-	-	-	-	-	-	-	-	-	-	-	-	-	-	-	-	-	-	-	1	3	2	1	1	1	-	13
Phytophthora infestans T30-4	Y	7	24	2	-	-	-	-	-	-	-	-	-	-	-	-	-	-	-	-	-	-	-	-	-	-	-	-	-	-	-	1	3	2	1	1	1	-	13
Phytophthora sojae P6497	Y	7	24	2	-	-	-	-	-	-	-	-	-	-	-	-	-	-	-	-	-	-	-	-	-	-	-	-	-	-	-	1	3	2	1	1	1	-	13
