# Supplementary material for: Drawing the tree of eukaryotic life based on the analysis of 2,269 manually annotated myosins from 328 species
Source: Genome Biol. 2007 Sep 18;8(9):R196. doi: 10.1186/gb-2007-8-9-r196 (PMC2375034; doi:10.1186/gb-2007-8-9-r196)
Supplement: Additional data file 4 — All organisms and related publications with references and links to genome sequencing centers. [file gb-2007-8-9-r196-S4.pdf]

***Acanthamoeba castellanii* (Ac)**
Project/s:
TBestDB - Taxonomically Broad EST Database: Acanthamoeba castellanii (http://tbestdb.bcm.umontreal.ca/searches/organism.php?orgID=AC)

***Cryptosporidium hominis TU502* (Ch)**
Project/s:
National Center for Biotechnology Information: NCBI Protozoa genomes (http://www.ncbi.nlm.nih.gov/sutils/blast_table.cgi?taxid=Protozoa)
Virginia Commonwealth University: Cryptosporidium hominis Research (http://www.hominis.mic.vcu.edu/)
Publication/s:
Xu P *et. al.* , *Nature* , **431** , 1107 (2004).

***Cryptosporidium parvum* (Cp)**
Project/s:
International Species Sequencing Consortium: Cryptosporidium parvum Sequencing Consortium ()
National Center for Biotechnology Information: NCBI Protozoa genomes (http://www.ncbi.nlm.nih.gov/sutils/blast_table.cgi?taxid=Protozoa)
The Institute for Genomic Research: TIGR Cryptosporidium parvum Gene Index (http://www.tigr.org/tigr-scripts/tgi/T_index.cgi?species=c_parvum)
Publication/s:
Abrahamsen MS *et. al.* , *Science* , **304** , 441 (2004).

***Eimeria tenella* (Et)**
Project/s:
The Institute for Genomic Research: TIGR Eimeria tenella Gene Index (http://www.tigr.org/tigr-scripts/tgi/T_index.cgi?species=e_tenella)
The Wellcome Trust Sanger Institute: The Eimeria tenella Genome Project (http://www.sanger.ac.uk/Projects/E_tenella/)

***Neospora caninum* (Nca)**
Project/s:
The Institute for Genomic Research: TIGR Neospora caninum Gene Index (http://www.tigr.org/tigr-scripts/tgi/T_index.cgi?species=n_caninum)

***Toxoplasma gondii RH* (Tg)**
Project/s:
National Center for Biotechnology Information: NCBI Protozoa genomes (http://www.ncbi.nlm.nih.gov/sutils/blast_table.cgi?taxid=Protozoa)
The Institute for Genomic Research: TIGR Toxoplasma gondii Gene Index (http://www.tigr.org/tigr-scripts/tgi/T_index.cgi?species=t_gondii)
The Institute for Genomic Research: The Toxoplasma gondii Genome Project (http://www.tigr.org/tdb/e2k1/tga1/)
The Wellcome Trust Sanger Institute: Toxoplasma gondii Sequencing Project (http://www.sanger.ac.uk/Projects/T_gondii/)
ToxoDB: Toxoplasma gondii Genome resource (http://toxodb.org/)

***Gregarina polymorpha* (Gp)**

***Plasmodium reichenowi* (Plr)**
Project/s:
PlasmoDB The Plasmodium Genome Resource: PlasmoDB The Plasmodium Genome Resource (http://www.plasmodb.org/)
The Wellcome Trust Sanger Institute: Plasmodium reichenowi, Partial Genome Shotgun (http://www.sanger.ac.uk/Projects/P_reichenowi/)

***Plasmodium berghei str. ANKA* (Pb)**
Project/s:
GenBank - NIH genetic sequence database: (http://www.ncbi.nlm.nih.gov/BLAST/)
National Center for Biotechnology Information: NCBI Protozoa genomes (http://www.ncbi.nlm.nih.gov/sutils/blast_table.cgi?taxid=Protozoa)
PlasmoDB The Plasmodium Genome Resource: PlasmoDB The Plasmodium Genome Resource (http://www.plasmodb.org/)
The Institute for Genomic Research: TIGR Plasmodium berghei Gene Index (http://www.tigr.org/tigr-scripts/tgi/T_index.cgi?species=p_berghei)
The Wellcome Trust Sanger Institute: Plasmodium berghei, Genome Shotgun (http://www.sanger.ac.uk/Projects/P_berghei/)
Publication/s:
Hall N *et. al.* , *Science* , **307** , 82 (2005).

***Plasmodium vivax str. Salvador I* (Pv)**
Project/s:
GenBank - NIH genetic sequence database: (http://www.ncbi.nlm.nih.gov/BLAST/)
National Center for Biotechnology Information: NCBI Protozoa genomes (http://www.ncbi.nlm.nih.gov/sutils/blast_table.cgi?taxid=Protozoa)
PlasmoDB The Plasmodium Genome Resource: PlasmoDB The Plasmodium Genome Resource (http://www.plasmodb.org/)
The Institute for Genomic Research: Plasmodium vivax Genome Project (http://www.tigr.org/tdb/e2k1/pva1/)
The Institute for Genomic Research: TIGR Plasmodium vivax Gene Index (http://www.tigr.org/tigr-scripts/tgi/T_index.cgi?species=p_vivax)
Publication/s:
Cui L *et. al.* , *Mol Biochem Parasitol* , **144** , 1 (2005).

***Plasmodium gallinaceum* (Plg)**
Project/s:
PlasmoDB The Plasmodium Genome Resource: PlasmoDB The Plasmodium Genome Resource (http://www.plasmodb.org/)
The Wellcome Trust Sanger Institute: Plasmodium gallinaceum, Partial Genome Shotgun (http://www.sanger.ac.uk/Projects/P_gallinaceum/)

***Plasmodium chabaudi* (Plc)**
Project/s:
GenBank - NIH genetic sequence database: (http://www.ncbi.nlm.nih.gov/BLAST/)
National Center for Biotechnology Information: NCBI Protozoa genomes (http://www.ncbi.nlm.nih.gov/sutils/blast_table.cgi?taxid=Protozoa)
PlasmoDB The Plasmodium Genome Resource: PlasmoDB The Plasmodium Genome Resource (http://www.plasmodb.org/)
The Wellcome Trust Sanger Institute: Plasmodium chabaudi, Partial Genome Shotgun (http://www.sanger.ac.uk/Projects/P_chabaudi/)
Publication/s:
Hall N *et. al.* , *Science* , **307** , 82 (2005).

***Plasmodium knowlesi* (Pk)**
Project/s:
PlasmoDB The Plasmodium Genome Resource: PlasmoDB The Plasmodium Genome Resource (http://www.plasmodb.org/)
The Wellcome Trust Sanger Institute: Plasmodium knowlesi, Genome Shotgun (http://www.sanger.ac.uk/Projects/P_knowlesi/)

***Plasmodium falciparum 3D7* (Pf_a)**
Project/s:
International Species Sequencing Consortium: Plasmodium falciparum Sequencing Consortium ()
National Center for Biotechnology Information: NCBI Protozoa genomes (http://www.ncbi.nlm.nih.gov/sutils/blast_table.cgi?taxid=Protozoa)
PlasmoDB The Plasmodium Genome Resource: PlasmoDB The Plasmodium Genome Resource (http://www.plasmodb.org/)
The Wellcome Trust Sanger Institute: Plasmodium falciparum Genome Projects (http://www.sanger.ac.uk/Projects/P_falciparum/)
Publication/s:
Hall N *et. al.* , *Nature* , **419** , 527 (2002).
Gardner MJ *et. al.* , *Nature* , **419** , 531 (2002).
Hyman RW *et. al.* , *Nature* , **419** , 534 (2002).
Gardner MJ *et. al.* , *Nature* , **419** , 498 (2002).

***Plasmodium falciparum Ghanaian Isolate* (Pf_b)**
Project/s:
The Wellcome Trust Sanger Institute: Plasmodium falciparum Genome Projects (http://www.sanger.ac.uk/Projects/P_falciparum/)

***Plasmodium falciparum Dd2* (Pf_d)**
Project/s:
Broad Institute of Harvard and MIT: Plasmodium falciparum species Database (http://www.broad.mit.edu/annotation/genome/plasmodium_falciparum_spp/MultiHome.html)
National Center for Biotechnology Information: NCBI Protozoa genomes (http://www.ncbi.nlm.nih.gov/sutils/blast_table.cgi?taxid=Protozoa)

Plasmodium falciparum HB3 **(Pf_c)**
Project/s:
Broad Institute of Harvard and MIT: Plasmodium falciparum HB3 Database (http://www.broad.mit.edu/annotation/microbes/plasmodium_falciparum_hb3/)
National Center for Biotechnology Information: NCBI Protozoa genomes (http://www.ncbi.nlm.nih.gov/sutils/blast_table.cgi?taxid=Protozoa)

Plasmodium yoelii yoelii 17XNL **(Ply)**
Project/s:
International Species Sequencing Consortium: Plasmodium yoelii yoelii Sequencing Consortium ()
National Center for Biotechnology Information: NCBI Protozoa genomes (http://www.ncbi.nlm.nih.gov/sutils/blast_table.cgi?taxid=Protozoa)
PlasmoDB The Plasmodium Genome Resource: PlasmoDB The Plasmodium Genome Resource (http://www.plasmodb.org/)
The Institute for Genomic Research: TIGR Plasmodium yoelii Gene Index (http://www.tigr.org/tigr-scripts/tgi/T_index.cgi?species=p_yoelii)
The Institute for Genomic Research: The Plasmodium yoelii yoelii Genome Sequencing Program (http://www.tigr.org/tdb/e2k1/pya1/)
Publication/s:
Carlton JM *et. al.* , *Nature* , **419** , 512 (2002).

Babesia bovis str. Texas T2Bo **(Bb)**
Project/s:
The Wellcome Trust Sanger Institute: Babesia bovis EST Sequencing Project (http://www.sanger.ac.uk/Projects/B_bovis/)
Washington State University: Babesia bovis Genome Sequencing Project (http://www.vetmed.wsu.edu/research_vmp/babesia-bovis/)

Babesia bigemina **(Bab)**
Project/s:
The Wellcome Trust Sanger Institute: Babesia bigemina genome project (http://www.sanger.ac.uk/Projects/B_bigemina/)

Theileria parva str. Muguga **(Tep)**
Project/s:
International Species Sequencing Consortium: Theileria parva Sequencing Consortium ()
National Center for Biotechnology Information: NCBI Protozoa genomes (http://www.ncbi.nlm.nih.gov/sutils/blast_table.cgi?taxid=Protozoa)
The Institute for Genomic Research: The Theileria parva Genome Database (http://www.tigr.org/tdb/e2k1/tpa1/)
Publication/s:
Gardner MJ *et. al.* , *Science* , **309** , 134 (2005).

Theileria annulata str. Ankara **(Tha)**
Project/s:
International Species Sequencing Consortium: Theileria annulata Sequencing Consortium ()
The Wellcome Trust Sanger Institute: Theileria annulata Sequencing Projects (http://www.sanger.ac.uk/Projects/T_annulata/)
Publication/s:
Pain A *et. al.* , *Science* , **309** , 131 (2005).

Tetrahymena thermophila SB210 **(Tet)**
Project/s:
International Species Sequencing Consortium: Tetrahymena thermophila Sequencing Consortium ()
National Center for Biotechnology Information: NCBI Protozoa genomes (http://www.ncbi.nlm.nih.gov/sutils/blast_table.cgi?taxid=Protozoa)
TBestDB - Taxonomically Broad EST Database: Tetrahymena thermophila (http://tbestdb.bcm.umontreal.ca/searches/organism.php?orgID=TT)
The Institute for Genomic Research: TIGR Tetrahymena thermophila Gene Index (http://www.tigr.org/tigr-scripts/tgi/T_index.cgi?species=t_thermophila)
The Institute for Genomic Research: The Tetrahymena thermophila Genome Sequencing Project (http://www.tigr.org/tdb/e2k1/ttg/)
Publication/s:
Eisen JA *et. al.* , *PLoS Biol* , **4** , e286 (2006).

Paramecium tetraurelia **(Pt)**
Project/s:
Genoscope: Paramecium tetraurelia - a model ciliate (http://www.genoscope.cns.fr/externe/English/Projets/Projet_FN/organisme_FN.html)
Publication/s:
Aury JM *et. al.* , *Nature* , **444** , 171 (2006).

Karlodinium micrum **(Kam)**
Project/s:
TBestDB - Taxonomically Broad EST Database: Karlodinium micrum (http://tbestdb.bcm.umontreal.ca/searches/organism.php?orgID=KM)

Perkinsus marinus **(Prm)**
Project/s:
The Institute for Genomic Research: The Perkinsus marinus Genome Sequencing Project (http://www.tigr.org/tdb/e2k1/pmg/)
The Marine Genomics Project: Perkinsus marinus
(http://www.marinegenomics.org/speciesentry.php?&s=y&organism=est_p_marinus&abrev=P.%20marinus&srn=Perkinsus%20marinus)

Entamoeba invadens IP1 **(Eti)**
Project/s:
GenBank - NIH genetic sequence database: (http://www.ncbi.nlm.nih.gov/BLAST/)
National Center for Biotechnology Information: NCBI Protozoa genomes (http://www.ncbi.nlm.nih.gov/sutils/blast_table.cgi?taxid=Protozoa)
Publication/s:
Wang Z *et. al.* , *Mol Biochem Parasitol* , **129** , 23 (2003).

Entamoeba dispar SAW760 **(Ed)**
Project/s:
National Center for Biotechnology Information: NCBI Protozoa genomes (http://www.ncbi.nlm.nih.gov/sutils/blast_table.cgi?taxid=Protozoa)

Entamoeba histolytica HM-1:IMSS **(Eh)**
Project/s:
International Species Sequencing Consortium: Entamoeba histolytica Sequencing Consortium ()
National Center for Biotechnology Information: NCBI Protozoa genomes (http://www.ncbi.nlm.nih.gov/sutils/blast_table.cgi?taxid=Protozoa)
The Institute for Genomic Research: Entamoeba histolytica Genome Project (http://www.tigr.org/tdb/e2k1/eha1/)
The Wellcome Trust Sanger Institute: Entamoeba histolytica Whole Genome Shotgun (http://www.sanger.ac.uk/Projects/E_histolytica/)
Publication/s:
Loftus B *et. al.* , *Nature* , **2005** , 865 (2005).

Leishmania infantum JPCM5 **(Lei)**
Project/s:
The Institute for Genomic Research: TIGR Leishmania sp. Gene Index (http://www.tigr.org/tigr-scripts/tgi/T_index.cgi?species=leishmania)
The Wellcome Trust Sanger Institute: The Leishmania infantum Genome Project (http://www.sanger.ac.uk/Projects/L_infantum/)

Leishmania major str. Friedlin **(Lem)**
Project/s:
International Species Sequencing Consortium: Leishmania major Sequencing Consortium ()
National Center for Biotechnology Information: NCBI Protozoa genomes (http://www.ncbi.nlm.nih.gov/sutils/blast_table.cgi?taxid=Protozoa)
The Institute for Genomic Research: TIGR Leishmania sp. Gene Index (http://www.tigr.org/tigr-scripts/tgi/T_index.cgi?species=leishmania)
The Wellcome Trust Sanger Institute: The Leishmania major Friedlin Genome Project (http://www.sanger.ac.uk/Projects/L_major/)
Publication/s:
El-Sayed NM *et. al.* , *Science* , **309** , 404 (2005).
Ivens AC *et. al.* , *Science* , **309** , 436 (2005).

Leishmania braziliensis **(Lb)**
Project/s:
The Wellcome Trust Sanger Institute: The Leishmania braziliensis Genome Project (http://www.sanger.ac.uk/Projects/L_braziliensis/)

***Trypanosoma vivax* (Tyv)**
Project/s:
The Wellcome Trust Sanger Institute: The Trypanosoma vivax Partial Genome Project (http://www.sanger.ac.uk/Projects/T_vivax/)

***Trypanosoma congolense* (Tyc)**
Project/s:
The Wellcome Trust Sanger Institute: The Trypanosoma congolense Partial Genome Project (http://www.sanger.ac.uk/Projects/T_congolense/)

***Trypanosoma cruzi CL Brener* (Trc)**
Project/s:
European Bioinformatics Institute: The Trypanosoma cruzi Proteome Classification and Analysis (http://www.ebi.ac.uk/parasites/TcGN/Proteome/proteome.html)
GenBank - NIH genetic sequence database: (http://www.ncbi.nlm.nih.gov/BLAST/)
International Species Sequencing Consortium: Trypanosoma cruzi Sequencing Consortium ()
National Center for Biotechnology Information: NCBI Protozoa genomes (http://www.ncbi.nlm.nih.gov/sutils/blast_table.cgi?taxid=Protozoa)
The Institute for Genomic Research: TIGR Trypanosoma cruzi Gene Index (http://www.tigr.org/tigr-scripts/tgi/T_index.cgi?species=t_cruzi)
The Institute for Genomic Research: The Trypanosoma cruzi Genome Project (http://www.tigr.org/tdb/e2k1/tca1/)
Publication/s:
El-Sayed NM *et. al.* , *Science* , **309** , 409 (2005).
El-Sayed NM *et. al.* , *Science* , **309** , 404 (2005).
Aguero F *et. al.* , *Mol Biochem Parasitol* , **136** , 221 (2004).
Porcel BM *et. al.* , *Genome Res* , **10** , 1103 (2000).

***Trypanosoma brucei TREU927* (Tb)**
Project/s:
International Species Sequencing Consortium: Trypanosoma brucei Sequencing Consortium ()
National Center for Biotechnology Information: NCBI Protozoa genomes (http://www.ncbi.nlm.nih.gov/sutils/blast_table.cgi?taxid=Protozoa)
The Institute for Genomic Research: TIGR Trypanosoma brucei Gene Index (http://www.tigr.org/tigr-scripts/tgi/T_index.cgi?species=t_brucei)
The Institute for Genomic Research: The TIGR Trypanosoma brucei Genome Project (http://www.tigr.org/tdb/e2k1/tba1/)
The Wellcome Trust Sanger Institute: The Trypanosoma brucei Genome Project (http://www.sanger.ac.uk/Projects/T_brucei/)
Publication/s:
El-Sayed NM *et. al.* , *Science* , **309** , 404 (2005).
Berriman M *et. al.* , *Science* , **309** , 416 (2005).

***Trypanosoma brucei gambiense* (Tbg)**
Project/s:
The Wellcome Trust Sanger Institute: The Trypanosoma brucei gambiense Partial Genome Project (http://www.sanger.ac.uk/Projects/T_b_gambiense/)

***Monosiga brevicollis* (Mb)**
Project/s:
DOE Joint Genome Institute: Monosiga brevicollis (http://genome.jgi-psf.org/Monbr1/Monbr1.home.html)

***Wangiella dermatitidis* (Wd)**

***Mycosphaerella graminicola* (Mg)**
Project/s:
DOE Joint Genome Institute: Mycosphaerella graminicola (http://genome.jgi-psf.org/Mycgr1/Mycgr1.home.html)
GenBank - NIH genetic sequence database: (http://www.ncbi.nlm.nih.gov/BLAST/)
Publication/s:
Keon J *et. al.* , *Fungal Genet Biol* , **42** , 376 (2005).

***Phaeosphaeria nodorum SN15* (Pn)**
Project/s:
Broad Institute of Harvard and MIT: Stagonospora nodorum Database (http://www.broad.mit.edu/annotation/fungi/stagonospora_nodorum/)
National Center for Biotechnology Information: NCBI Fungi Genomes Project (http://www.ncbi.nlm.nih.gov/sutils/genom_table.cgi?organism=fungi)

***Alternaria brassicicola ATCC 96836* (Alb)**
Project/s:
The Genome Sequencing Center at Washington University: Alternaria brassicicola (http://genome.wustl.edu/genome.cgi?GENOME=Alternaria%20brassicicola&GROUP=5)

***Emericella nidulans FGSC A4* (En)**
Project/s:
Broad Institute of Harvard and MIT: Aspergillus nidulans Database (http://www.broad.mit.edu/annotation/fungi/aspergillus/)
GenBank - NIH genetic sequence database: (http://www.ncbi.nlm.nih.gov/BLAST/)
International Species Sequencing Consortium: Emericella nidulans Sequencing Consortium ()
National Center for Biotechnology Information: NCBI Fungi Genomes Project (http://www.ncbi.nlm.nih.gov/sutils/genom_table.cgi?organism=fungi)
The Institute for Genomic Research: TIGR Aspergillus nidulans Gene Index (http://www.tigr.org/tigr-scripts/tgi/T_index.cgi?species=a_nidulans)
Publication/s:
Galagan JE *et. al.* , *Nature* , **438** , 1105 (2005).
Kupfer DM *et. al.* , *Eukaryot Cell* , **3** , 1088 (2004).

***Neosartorya fischeri NRRL 181* (Nef)**
Project/s:
National Center for Biotechnology Information: NCBI Fungi Genomes Project (http://www.ncbi.nlm.nih.gov/sutils/genom_table.cgi?organism=fungi)
The Aspergillus Website: Aspergillus Genomics - Neosartorya fischeri / Aspergillus fischerianus (http://www.aspergillus.man.ac.uk/indexhome.htm?secure/sequence_info/fischerianus.htm~main)

***Aspergillus niger ATCC 1015* (An)**
Project/s:
DOE Joint Genome Institute: Aspergillus niger (http://genome.jgi-psf.org/Aspni1/Aspni1.home.html)
Fungal Genomics Project: Aspergillus niger (https://fungalgenomics.concordia.ca/fungi/Anig.php)

***Aspergillus terreus NIH2624* (Ast)**
Project/s:
Broad Institute of Harvard and MIT: Aspergillus terreus Database (http://www.broad.mit.edu/annotation/fungi/aspergillus_terreus/)
National Center for Biotechnology Information: NCBI Fungi Genomes Project (http://www.ncbi.nlm.nih.gov/sutils/genom_table.cgi?organism=fungi)

***Aspergillus clavatus NRRL 1* (Asc)**
Project/s:
National Center for Biotechnology Information: NCBI Fungi Genomes Project (http://www.ncbi.nlm.nih.gov/sutils/genom_table.cgi?organism=fungi)
The Aspergillus Website: Aspergillus Genomics - Aspergillus clavatus (http://www.aspergillus.man.ac.uk/indexhome.htm?secure/sequence_info/clavatus.htm~main)

***Aspergillus flavus NRRL3357* (Af)**
Project/s:
GenBank - NIH genetic sequence database: (http://www.ncbi.nlm.nih.gov/BLAST/)
National Center for Biotechnology Information: NCBI Fungi Genomes Project (http://www.ncbi.nlm.nih.gov/sutils/genom_table.cgi?organism=fungi)
The Institute for Genomic Research: TIGR Aspergillus flavus Gene Index (http://www.tigr.org/tigr-scripts/tgi/T_index.cgi?species=a_flavus)
Publication/s:
Yu J *et. al.* , *FEMS Microbiol Lett* , **237** , 333 (2004).

***Aspergillus fumigatus Af293* (Asf)**
Project/s:
International Species Sequencing Consortium: Aspergillus fumigatus Sequencing Consortium ()
National Center for Biotechnology Information: NCBI Fungi Genomes Project (http://www.ncbi.nlm.nih.gov/sutils/genom_table.cgi?organism=fungi)
The Institute for Genomic Research: Aspergillus fumigatus Genome Project (http://www.tigr.org/tdb/e2k1/afu1/)

The Wellcome Trust Sanger Institute: Aspergillus fumigatus Genome Project (http://www.sanger.ac.uk/Projects/A_fumigatus/)
Publication/s:
Nierman WC *et. al.* , *Nature* , **438** , 1151 (2005).

Aspergillus oryzae RIB40 **(Ao)**
Project/s:
Database of the Genomes Analyzed at NITE: Aspergillus oryzae RIB40 (http://www.bio.nite.go.jp/dogan/MicroTop?GENOME_ID=ao)
Publication/s:
Machida M *et. al.* , *Nature* , **438** , 1157 (2005).

Ajellomyces capsulatus NAmI WU24 **(Ajc_c)**
Project/s:
Broad Institute of Harvard and MIT: Histoplasma capsulatum Database (http://www.broad.mit.edu/annotation/fungi/histoplasma_capsulatum/)
National Center for Biotechnology Information: NCBI Fungi Genomes Project (http://www.ncbi.nlm.nih.gov/sutils/genom_table.cgi?organism=fungi)

Ajellomyces capsulatus NAmII G217B **(Ajc_b)**
Project/s:
The Genome Sequencing Center at Washington University: Histoplasma capsulatum Sequencing (http://genome.wustl.edu/genome.cgi?GENOME=Histoplasma%20capsulatum)

Ajellomyces capsulatus NAmII G186AR **(Ajc_a)**
Project/s:
The Genome Sequencing Center at Washington University: Histoplasma capsulatum Sequencing (http://genome.wustl.edu/genome.cgi?GENOME=Histoplasma%20capsulatum)

Ascosphaera apis USDA-ARSEF 7405 **(Asa)**
Project/s:
Human Genome Sequencing Center at Baylor College of Medicine: Ascosphaera apis (http://www.hgsc.bcm.tmc.edu/projects/microbial/Aapis/)
National Center for Biotechnology Information: NCBI Fungi Genomes Project (http://www.ncbi.nlm.nih.gov/sutils/genom_table.cgi?organism=fungi)

Uncinocarpus reesii 1704 **(Ur)**
Project/s:
Broad Institute of Harvard and MIT: Uncinocarpus reesii Sequencing Project (http://www.broad.mit.edu/annotation/fungi/uncinocarpus_reesii/)
National Center for Biotechnology Information: NCBI Fungi Genomes Project (http://www.ncbi.nlm.nih.gov/sutils/genom_table.cgi?organism=fungi)

Coccidioides immitis RS **(Coi_a)**
Project/s:
Broad Institute of Harvard and MIT: Coccidioides immitis Sequencing Project (http://www.broad.mit.edu/annotation/fungi/coccidioides_immitis/)
National Center for Biotechnology Information: NCBI Fungi Genomes Project (http://www.ncbi.nlm.nih.gov/sutils/genom_table.cgi?organism=fungi)

Coccidioides immitis H538.4 **(Coi_b)**
Project/s:
Broad Institute of Harvard and MIT: Coccidioides group Database (http://www.broad.mit.edu/annotation/genome/coccidioides_group/MultiHome.html)
National Center for Biotechnology Information: NCBI Fungi Genomes Project (http://www.ncbi.nlm.nih.gov/sutils/genom_table.cgi?organism=fungi)

Coccidioides immitis RMSCC 2394 **(Coi_c)**
Project/s:
Broad Institute of Harvard and MIT: Coccidioides group Database (http://www.broad.mit.edu/annotation/genome/coccidioides_group/MultiHome.html)
National Center for Biotechnology Information: NCBI Fungi Genomes Project (http://www.ncbi.nlm.nih.gov/sutils/genom_table.cgi?organism=fungi)

Coccidioides posadasii C735 **(Cop)**
Project/s:
The Institute for Genomic Research: Coccidioides posadasii Genome Project (http://www.tigr.org/tdb/e2k1/cpa1/)
The Institute for Genomic Research: TIGR Coccidioides posadasii Gene Index (http://www.tigr.org/tigr-scripts/tgi/T_index.cgi?species=c_immitis)

Paracoccidioides brasiliensis **(Pab)**
Project/s:
GenBank - NIH genetic sequence database: (http://www.ncbi.nlm.nih.gov/BLAST/)
Publication/s:
Felipe MS *et. al.* , *J Biol Chem* , **280** , 24706 (2005).

Blumeria graminis **(Bg)**

Botryotinia fuckeliana B05.10 **(Bof)**
Project/s:
Broad Institute of Harvard and MIT: Botrytis cinerea (http://www.broad.mit.edu/cgi-bin/annotation/fungi/botrytis_cinerea/download_license.cgi)
National Center for Biotechnology Information: NCBI Fungi Genomes Project (http://www.ncbi.nlm.nih.gov/sutils/genom_table.cgi?organism=fungi)

Sclerotinia sclerotiorum 1980 **(Scs)**
Project/s:
Broad Institute of Harvard and MIT: Sclerotinia sclerotiorum Sequencing Project (http://www.broad.mit.edu/annotation/fungi/sclerotinia_sclerotiorum/)
GenBank - NIH genetic sequence database: (http://www.ncbi.nlm.nih.gov/BLAST/)
National Center for Biotechnology Information: NCBI Fungi Genomes Project (http://www.ncbi.nlm.nih.gov/sutils/genom_table.cgi?organism=fungi)
Publication/s:
Li R *et. al.* , *Fungal Genet Biol* , **41** , 735 (2004).

Hypocrea jecorina QM9414 **(Hj)**
Project/s:
DOE Joint Genome Institute: Trichoderma reesei (http://genome.jgi-psf.org/Trire2/Trire2.home.html)
GenBank - NIH genetic sequence database: (http://www.ncbi.nlm.nih.gov/BLAST/)
National Center for Biotechnology Information: NCBI Fungi Genomes Project (http://www.ncbi.nlm.nih.gov/sutils/genom_table.cgi?organism=fungi)
Publication/s:
Diener SE *et. al.* , *FEMS Microbiol Lett* , **230** , 275 (2004).
Foreman PK *et. al.* , *J Biol Chem* , **278** , 31988 (2003).

Gibberella zeae PH-1 **(Gz)**
Project/s:
Broad Institute of Harvard and MIT: Fusarium graminearum Sequencing Project (http://www.broad.mit.edu/annotation/fungi/fusarium/index.html)
National Center for Biotechnology Information: NCBI Fungi Genomes Project (http://www.ncbi.nlm.nih.gov/sutils/genom_table.cgi?organism=fungi)

Gibberella moniliformis 7600 **(Gim)**
Project/s:
Broad Institute of Harvard and MIT: Fusarium verticillioides Sequencing Project (http://www.broad.mit.edu/annotation/fungi/fusarium_verticillioides/)
GenBank - NIH genetic sequence database: (http://www.ncbi.nlm.nih.gov/BLAST/)
National Center for Biotechnology Information: NCBI Fungi Genomes Project (http://www.ncbi.nlm.nih.gov/sutils/genom_table.cgi?organism=fungi)
The Institute for Genomic Research: TIGR Fusarium verticillioides Gene Index (http://www.tigr.org/tigr-scripts/tgi/T_index.cgi?species=f_verticill)
Publication/s:
Brown DW *et. al.* , *Fungal Genet Biol* , **42** , 848 (2005).

Nectria haematococca MPVI **(Nh)**
Project/s:
DOE Joint Genome Institute: Nectria haematococca (http://genome.jgi-psf.org/Necha1/Necha1.home.html)

Fusarium oxysporum **(Fo)**

Magnaporthe grisea 70-15 **(Mag)**

Project/s:
Broad Institute of Harvard and MIT: Magnaporthe grisea Sequencing Project (http://www.broad.mit.edu/annotation/fungi/magnaporthe/)
GenBank - NIH genetic sequence database: (http://www.ncbi.nlm.nih.gov/BLAST/)
International Species Sequencing Consortium: Magnaporthe grisea Sequencing Consortium ()
National Center for Biotechnology Information: NCBI Fungi Genomes Project (http://www.ncbi.nlm.nih.gov/sutils/genom_table.cgi?organism=fungi)
The Institute for Genomic Research: TIGR Magnaporthe grisea Gene Index (http://www.tigr.org/tigr-scripts/tgi/T_index.cgi?species=m_grisea)
Publication/s:
Dean RA *et. al.* , *Nature* , **434** , 980 (2005).
Ebbole DJ *et. al.* , *Mol Plant Microbe Interact* , **17** , 1337 (2004).

***Glomerella graminicola* (Glg)**

***Colletotrichum trifolii* (Cot)**

***Chaetomium globosum CBS 148.51* (Chg)**
Project/s:
Broad Institute of Harvard and MIT: Chaetomium globosum Sequencing Project (http://www.broad.mit.edu/annotation/fungi/chaetomium_globosum/)
National Center for Biotechnology Information: NCBI Fungi Genomes Project (http://www.ncbi.nlm.nih.gov/sutils/genom_table.cgi?organism=fungi)

***Podospora anserina* (Poa)**
Project/s:
Genoscope: Podospora anserina Genome Project (http://www.genoscope.cns.fr/externe/English/Projets/Projet_GA/organisme_GA.html)

***Neurospora crassa OR74A* (Nc)**
Project/s:
Broad Institute of Harvard and MIT: Neurospora crassa Sequencing Project (http://www.broad.mit.edu/annotation/fungi/neurospora_crassa_7/)
GenBank - NIH genetic sequence database: (http://www.ncbi.nlm.nih.gov/BLAST/)
International Species Sequencing Consortium: Neurospora crassa Sequencing Consortium ()
National Center for Biotechnology Information: NCBI Fungi Genomes Project (http://www.ncbi.nlm.nih.gov/sutils/genom_table.cgi?organism=fungi)
The Institute for Genomic Research: TIGR Neurospora crassa Gene Index (http://www.tigr.org/tigr-scripts/tgi/T_index.cgi?species=neurospora)
Publication/s:
Kupfer DM *et. al.* , *Eukaryot Cell* , **3** , 1088 (2004).
Galagan JE *et. al.* , *Nature* , **422** , 859 (2003).

***Yarrowia lipolytica CLIB99* (Yl)**
Project/s:
Genolevures: Genomic Exploration of the Hemiascomycete Yeasts (http://cbi.labri.u-bordeaux.fr/Genolevures/blast.php)
National Center for Biotechnology Information: NCBI Fungi Genomes Project (http://www.ncbi.nlm.nih.gov/sutils/genom_table.cgi?organism=fungi)
Publication/s:
Dujon B *et. al.* , *Nature* , **430** , 35 (2004).
Casaregola S *et. al.* , *FEBS Lett* , **487** , 95 (2000).

***Clavispora lusitaniae ATCC 42720* (Cll)**
Project/s:
Broad Institute of Harvard and MIT: Candida lusitaniae Sequencing Project (http://www.broad.mit.edu/annotation/fungi/candida_lusitaniae/)
National Center for Biotechnology Information: NCBI Fungi Genomes Project (http://www.ncbi.nlm.nih.gov/sutils/genom_table.cgi?organism=fungi)

***Debaryomyces hansenii CBS767* (Deh)**
Project/s:
Genolevures: Genomic Exploration of the Hemiascomycete Yeasts (http://cbi.labri.u-bordeaux.fr/Genolevures/blast.php)
National Center for Biotechnology Information: NCBI Fungi Genomes Project (http://www.ncbi.nlm.nih.gov/sutils/genom_table.cgi?organism=fungi)
Publication/s:
Dujon B *et. al.* , *Nature* , **430** , 35 (2004).
Lepingle A *et. al.* , *FEBS Lett* , **487** , 82 (2000).

***Eremothecium gossypii ATCC 10895* (Erg)**
Project/s:
Ashbya Genome Database: Ashbya Genome Database (http://agd.unibas.ch/)
International Species Sequencing Consortium: Eremothecium gossypii Sequencing Consortium ()
National Center for Biotechnology Information: NCBI Fungi Genomes Project (http://www.ncbi.nlm.nih.gov/sutils/genom_table.cgi?organism=fungi)
Publication/s:
Dietrich FS *et. al.* , *Science* , **304** , 304 (2004).

***Kluyveromyces thermotolerans CBS 6340* (Kt)**
Project/s:
Genolevures: Genomic Exploration of the Hemiascomycete Yeasts (http://cbi.labri.u-bordeaux.fr/Genolevures/blast.php)
Publication/s:
Malpertuy A *et. al.* , *FEBS Letters* , **487** , 61 (2000).

***Kluyveromyces lactis NRRL Y-1140* (Kl)**
Project/s:
Genolevures: Genomic Exploration of the Hemiascomycete Yeasts (http://cbi.labri.u-bordeaux.fr/Genolevures/blast.php)
National Center for Biotechnology Information: NCBI Fungi Genomes Project (http://www.ncbi.nlm.nih.gov/sutils/genom_table.cgi?organism=fungi)
Publication/s:
Dujon B *et. al.* , *Nature* , **430** , 35 (2004).
Bolotin-Fukuhara M *et. al.* , *FEBS Lett* , **487** , 66 (2000).

***Kluyveromyces marxianus CBS712* (Km)**
Project/s:
Genolevures: Genomic Exploration of the Hemiascomycete Yeasts (http://cbi.labri.u-bordeaux.fr/Genolevures/blast.php)
Publication/s:
Llorente B *et. al.* , *FEBS Letters* , **487** , 71 (2000).

***Kluyveromyces waltii NCYC 2644* (Kw)**
Project/s:
International Species Sequencing Consortium: The Broad Institute Kluyveromyces waltii Sequencing Initiative ()
National Center for Biotechnology Information: NCBI Fungi Genomes Project (http://www.ncbi.nlm.nih.gov/sutils/genom_table.cgi?organism=fungi)
Publication/s:
Manolis Kellis, Bruce W. Birren, Eric S. Lander , *Nature* , **428** , 617 (2004).

***Lodderomyces elongisporus NRLL YB-4239* (Loe)**
Project/s:
Broad Institute of Harvard and MIT: Lodderomyces elongisporus Database (http://www.broad.mit.edu/annotation/genome/lodderomyces_elongisporus/Home.html)
National Center for Biotechnology Information: NCBI Fungi Genomes Project (http://www.ncbi.nlm.nih.gov/sutils/genom_table.cgi?organism=fungi)

***Naumovia castellii NRRL Y-12630* (Nac)**
Project/s:
International Species Sequencing Consortium: Washington University Naumovia castellii Sequencing ()
National Center for Biotechnology Information: NCBI Fungi Genomes Project (http://www.ncbi.nlm.nih.gov/sutils/genom_table.cgi?organism=fungi)
Publication/s:
Cliften *et. al.* , *Science* , **301** , 71 (2003).

***Pichia stipitis CBS 6054* (Pcs)**
Project/s:

DOE Joint Genome Institute: Pichia stipitis (http://genome.jgi-psf.org/Picst3/Picst3.home.html)

***Pichia guilliermondii ATCC 6260* (Pig)**
Project/s:
Broad Institute of Harvard and MIT: Candida guilliermondii Sequencing Project (http://www.broad.mit.edu/annotation/fungi/candida_guilliermondii/)
National Center for Biotechnology Information: NCBI Fungi Genomes Project (http://www.ncbi.nlm.nih.gov/sutils/genom_table.cgi?organism=fungi)

***Pichia farinosa CBS7064* (Pif)**
Project/s:
Genolevures: Genomic Exploration of the Hemiascomycete Yeasts (http://cbi.labri.u-bordeaux.fr/Genolevures/blast.php)
Publication/s:
de Montigny J *et. al.* , *FEBS Lett* , **487** , 87 (2000).

***Pichia angusta CBS 4732* (Pia)**
Project/s:
Genolevures: Genomic Exploration of the Hemiascomycete Yeasts (http://cbi.labri.u-bordeaux.fr/Genolevures/blast.php)
Publication/s:
Blandin G *et. al.* , *FEBS Letters* , **487** , 76 (2000).

***Saccharomyces paradoxus NRRL Y-17217* (Sap)**
Project/s:
Broad Institute of Harvard and MIT: Yeast Comparative Genomics (http://www.broad.mit.edu/annotation/fungi/comp_yeasts/)
National Center for Biotechnology Information: NCBI Fungi Genomes Project (http://www.ncbi.nlm.nih.gov/sutils/genom_table.cgi?organism=fungi)
Publication/s:
Kellis *et. al.* , *Nature* , **423** , 241 (2003).

***Saccharomyces kudriavzevii IFO 1802* (Sak)**
Project/s:
International Species Sequencing Consortium: Washington University Saccharomyces kudriavzevii Sequencing ()
National Center for Biotechnology Information: NCBI Fungi Genomes Project (http://www.ncbi.nlm.nih.gov/sutils/genom_table.cgi?organism=fungi)
Publication/s:
Cliften *et. al.* , *Science* , **301** , 71 (2003).

***Saccharomyces servazzii CBS4311* (Sse)**
Project/s:
Genolevures: Genomic Exploration of the Hemiascomycete Yeasts (http://cbi.labri.u-bordeaux.fr/Genolevures/blast.php)
Publication/s:
Casaregola S *et. al.* , *FEBS Letters* , **487** , 47 (2000).

***Saccharomyces mikatae IFO 1815* (Smi)**
Project/s:
Broad Institute of Harvard and MIT: Yeast Comparative Genomics (http://www.broad.mit.edu/annotation/fungi/comp_yeasts/)
International Species Sequencing Consortium: Washington University Saccharomyces mikatae Sequencing ()
National Center for Biotechnology Information: NCBI Fungi Genomes Project (http://www.ncbi.nlm.nih.gov/sutils/genom_table.cgi?organism=fungi)
Publication/s:
Cliften *et. al.* , *Science* , **301** , 71 (2003).

***Kazachstania exigua CBS379* (Ke)**
Project/s:
Genolevures: Genomic Exploration of the Hemiascomycete Yeasts (http://cbi.labri.u-bordeaux.fr/Genolevures/blast.php)
Publication/s:
Bon E *et. al.* , *FEBS Lett* , **487** , 42 (2000).

***Saccharomyces bayanus 623-6C* (Sab_a)**
Project/s:
International Species Sequencing Consortium: Washington University Saccharomyces bayanus Sequencing ()
National Center for Biotechnology Information: NCBI Fungi Genomes Project (http://www.ncbi.nlm.nih.gov/sutils/genom_table.cgi?organism=fungi)
Publication/s:
Cliften *et. al.* , *Science* , **301** , 71 (2003).

***Saccharomyces bayanus MCYC 623* (Sab_b)**
Project/s:
Broad Institute of Harvard and MIT: Yeast Comparative Genomics (http://www.broad.mit.edu/annotation/fungi/comp_yeasts/)
National Center for Biotechnology Information: NCBI Fungi Genomes Project (http://www.ncbi.nlm.nih.gov/sutils/genom_table.cgi?organism=fungi)
Publication/s:
Kellis *et. al.* , *Nature* , **423** , 241 (2003).

***Saccharomyces bayanus var. uvarum* (Suv)**
Project/s:
Genolevures: Genomic Exploration of the Hemiascomycete Yeasts (http://cbi.labri.u-bordeaux.fr/Genolevures/blast.php)
Publication/s:
Bon E *et. al.* , *FEBS Letters* , **487** , 37 (2000).

***Saccharomyces cerevisiae RM11-1a* (Sc_b)**
Project/s:
Broad Institute of Harvard and MIT: Saccharomyces cerevisiae RM11-1a Sequencing Project (http://www.broad.mit.edu/annotation/fungi/saccharomyces_cerevisiae_rm11_1a/)
National Center for Biotechnology Information: NCBI Fungi Genomes Project (http://www.ncbi.nlm.nih.gov/sutils/genom_table.cgi?organism=fungi)
The Institute for Genomic Research: TIGR Saccharomyces cerevisiae Gene Index (http://www.tigr.org/tigr-scripts/tgi/T_index.cgi?species=yeast)

***Saccharomyces cerevisiae YJM789* (Sc_a)**
Project/s:
National Center for Biotechnology Information: NCBI Fungi Genomes Project (http://www.ncbi.nlm.nih.gov/sutils/genom_table.cgi?organism=fungi)
The Institute for Genomic Research: TIGR Saccharomyces cerevisiae Gene Index (http://www.tigr.org/tigr-scripts/tgi/T_index.cgi?species=yeast)

***Saccharomyces cerevisiae S288c* (Sc_c)**
Project/s:
e! Ensembl: S.cerevisiae (http://www.ensembl.org/Saccharomyces_cerevisiae/index.html)
Genolevures: Re-Annotation of the Saccharomyces cerevisiae Genome ()
International Species Sequencing Consortium: Saccharomyces cerevisiae Sequencing Consortium ()
National Center for Biotechnology Information: NCBI Fungi Genomes Project (http://www.ncbi.nlm.nih.gov/sutils/genom_table.cgi?organism=fungi)
Saccharomyces Genome Database: Database of the molecular biology and genetics of the yeast Saccharomyces cerevisiae (http://www.yeastgenome.org/)
The Institute for Genomic Research: TIGR Saccharomyces cerevisiae Gene Index (http://www.tigr.org/tigr-scripts/tgi/T_index.cgi?species=yeast)
Publication/s:
Blandin G *et. al.* , *FEBS Lett* , **487** , 31 (2000).
Dujon B *et. al.* , *Nature* , **387** , 98 (1997).
Tettelin *et. al.* , *Nature* , **387** , 81 (1997).
Goffeau *et. al.* , *Science* , **274** , 546 (1996).

***Saccharomyces kluyveri NRRL Y-12651* (Sk_a)**
Project/s:
International Species Sequencing Consortium: Washington University Saccharomyces kluyveri Sequencing ()
National Center for Biotechnology Information: NCBI Fungi Genomes Project (http://www.ncbi.nlm.nih.gov/sutils/genom_table.cgi?organism=fungi)
The Genome Sequencing Center at Washington University: Saccharomyces kluyveri (http://genome.wustl.edu/genome.cgi?GENOME=Saccharomyces%20kluyveri&GROUP=5)
Publication/s:

Cliften *et. al.* , *Science* , **301** , 71 (2003).

Zygosaccharomyces rouxii CBS732 **(Zr)**
Project/s:
Genolevures: Genomic Exploration of the Hemiascomycete Yeasts (http://cbi.labri.u-bordeaux.fr/Genolevures/blast.php)
Publication/s:
de Montigny J *et. al.* , *FEBS Letters* , **487** , 52 (2000).

Candida dubliniensis CD36 **(Cad)**
Project/s:
The Wellcome Trust Sanger Institute: Candida dubliniensis Genome Sequencing (http://www.sanger.ac.uk/sequencing/Candida/dubliniensis/)

Candida parapsilosis **(Cap)**
Project/s:
The Wellcome Trust Sanger Institute: Candida parapsilosis Sequencing (http://www.sanger.ac.uk/sequencing/Candida/parapsilosis/)

Candida glabrata CBS138 **(Cgl)**
Project/s:
Genolevures: Genomic Exploration of the Hemiascomycete Yeasts (http://cbi.labri.u-bordeaux.fr/Genolevures/blast.php)
National Center for Biotechnology Information: NCBI Fungi Genomes Project (http://www.ncbi.nlm.nih.gov/sutils/genom_table.cgi?organism=fungi)
Publication/s:
Dujon B *et. al.* , *Nature* , **430** , 35 (2004).

Candida albicans SC5314 **(Ca_a)**
Project/s:
National Center for Biotechnology Information: NCBI Fungi Genomes Project (http://www.ncbi.nlm.nih.gov/sutils/genom_table.cgi?organism=fungi)
Stanford Genome Technology Center: Sequencing of Candida Albicans (http://www-sequence.stanford.edu/group/candida/index.html)
Publication/s:
Jones T *et. al.* , *Proc Natl Acad Sci U S A* , **101** , 7329 (2004).

Candida albicans WO-1 **(Ca_b)**
Project/s:
Broad Institute of Harvard and MIT: Candida albicans Database (http://www.broad.mit.edu/annotation/genome/candida_albicans/Home.html)
National Center for Biotechnology Information: NCBI Fungi Genomes Project (http://www.ncbi.nlm.nih.gov/sutils/genom_table.cgi?organism=fungi)

Candida tropicalis MYA-3404 **(Ct_a)**
Project/s:
Broad Institute of Harvard and MIT: Candida tropicalis Sequencing Project (http://www.broad.mit.edu/annotation/fungi/candida_tropicalis/)
National Center for Biotechnology Information: NCBI Fungi Genomes Project (http://www.ncbi.nlm.nih.gov/sutils/genom_table.cgi?organism=fungi)

Schizosaccharomyces japonicus yFS275 **(Sj)**
Project/s:
Broad Institute of Harvard and MIT: Schizosaccharomyces japonicus Database (http://www.broad.mit.edu/annotation/genome/schizosaccharomyces_japonicus/Home.html)
National Center for Biotechnology Information: NCBI Fungi Genomes Project (http://www.ncbi.nlm.nih.gov/sutils/genom_table.cgi?organism=fungi)

Schizosaccharomyces pombe 972h- **(Sp)**
Project/s:
GenBank - NIH genetic sequence database: (http://www.ncbi.nlm.nih.gov/BLAST/)
International Species Sequencing Consortium: Schizosaccharomyces pombe Sequencing Consortium ()
National Center for Biotechnology Information: NCBI Fungi Genomes Project (http://www.ncbi.nlm.nih.gov/sutils/genom_table.cgi?organism=fungi)
The Institute for Genomic Research: TIGR Schizosaccharomyces pombe Gene Index (http://www.tigr.org/tigr-scripts/tgi/T_index.cgi?species=f_yeast)
The Wellcome Trust Sanger Institute: Schizosaccharomyces pombe Genome Project (http://www.sanger.ac.uk/Projects/S_pombe/)
Publication/s:
Kupfer DM *et. al.* , *Eukaryot Cell* , **3** , 1088 (2004).
Wood V *et. al.* , *Nature* , **415** , 871 (2002).

Filobasidiella neoformans var. bacillispora WM276 **(Fnb_c)**

Filobasidiella neoformans var. bacillispora ATCC32609 **(Fnb_a)**

Filobasidiella neoformans var. bacillispora R265 **(Fnb_b)**
Project/s:
Broad Institute of Harvard and MIT: Cryptococcus neoformans Serotype B Database (http://www.broad.mit.edu/annotation/fungi/cryptococcus_neoformans_b/)
National Center for Biotechnology Information: NCBI Fungi Genomes Project (http://www.ncbi.nlm.nih.gov/sutils/genom_table.cgi?organism=fungi)

Filobasidiella neoformans var. bacillispora E566 **(Fnb_d)**

Filobasidiella neoformans var. neoformans H99 **(Fna_b)**
Project/s:
Broad Institute of Harvard and MIT: Cryptococcus neoformans Serotype A Database (http://www.broad.mit.edu/annotation/genome/cryptococcus_neoformans/Home.html)
National Center for Biotechnology Information: NCBI Fungi Genomes Project (http://www.ncbi.nlm.nih.gov/sutils/genom_table.cgi?organism=fungi)
The Institute for Genomic Research: TIGR Cryptococcus sp. (Filobasidiella neoformans) Gene Index (http://www.tigr.org/tigr-scripts/tgi/T_index.cgi?species=cryptococcus)

Filobasidiella neoformans var. neoformans 125.91 **(Fna_a)**
Project/s:
The Institute for Genomic Research: TIGR Cryptococcus sp. (Filobasidiella neoformans) Gene Index (http://www.tigr.org/tigr-scripts/tgi/T_index.cgi?species=cryptococcus)

Filobasidiella neoformans var. neoformans B-3501A **(Fnd_b)**
Project/s:
GenBank - NIH genetic sequence database: (http://www.ncbi.nlm.nih.gov/BLAST/)
National Center for Biotechnology Information: NCBI Fungi Genomes Project (http://www.ncbi.nlm.nih.gov/sutils/genom_table.cgi?organism=fungi)
The Institute for Genomic Research: TIGR Cryptococcus sp. (Filobasidiella neoformans) Gene Index (http://www.tigr.org/tigr-scripts/tgi/T_index.cgi?species=cryptococcus)
Publication/s:
Kupfer DM *et. al.* , *Eukaryot Cell* , **3** , 1088 (2004).

Filobasidiella neoformans var. neoformans JEC21 **(Fnd_c)**
Project/s:
International Species Sequencing Consortium: Filobasidiella neoformans var. neoformans JEC21 Sequencing Consortium ()
National Center for Biotechnology Information: NCBI Fungi Genomes Project (http://www.ncbi.nlm.nih.gov/sutils/genom_table.cgi?organism=fungi)
The Institute for Genomic Research: TIGR Cryptococcus sp. (Filobasidiella neoformans) Gene Index (http://www.tigr.org/tigr-scripts/tgi/T_index.cgi?species=cryptococcus)
Publication/s:
Loftus BJ *et. al.* , *Science* , **307** , 1321 (2005).

Filobasidiella neoformans var. neoformans JEC20 **(Fnd_a)**
Project/s:
The Institute for Genomic Research: TIGR Cryptococcus sp. (Filobasidiella neoformans) Gene Index (http://www.tigr.org/tigr-scripts/tgi/T_index.cgi?species=cryptococcus)

Coprinopsis cinerea okayama7#130 **(Cpc)**
Project/s:
Broad Institute of Harvard and MIT: Coprinus cinereus Sequencing Project (http://www.broad.mit.edu/annotation/fungi/coprinus_cinereus/)
Fungal Genomics Project: Coprinus cinereus (https://fungalgenomics.concordia.ca/fungi/Ccin.php)
National Center for Biotechnology Information: NCBI Fungi Genomes Project (http://www.ncbi.nlm.nih.gov/sutils/genom_table.cgi?organism=fungi)

Laccaria bicolor S238N **(Lab)**

Project/s:
DOE Joint Genome Institute: Laccaria bicolor (http://genome.jgi-psf.org/Lacbi1/Lacbi1.home.html)
L'Institut National de la Recherche Agronomique: EctomycorrhizaDB (http://mycor.nancy.inra.fr/ectomycorrhizadb/)
L'Institut National de la Recherche Agronomique: Laccaria Genome Resources (http://mycor.nancy.inra.fr/IMGC/LaccariaGenome/index.html)

***Phanerochaete chrysosporium RP-78* (Phc)**
Project/s:
DOE Joint Genome Institute: Phanerochaete chrysosporium (http://genome.jgi-psf.org/Phchr1/Phchr1.home.html)
Fungal Genomics Project: Phanerochaete chrysosporium (https://fungalgenomics.concordia.ca/fungi/Pchr.php)
International Species Sequencing Consortium: Phanerochaete chrysosporium Sequencing Consortium ()
National Center for Biotechnology Information: NCBI Fungi Genomes Project (http://www.ncbi.nlm.nih.gov/sutils/genom_table.cgi?organism=fungi)
Publication/s:
Martinez D *et. al.* , *Nat Biotechnol* , **22** , 695 (2004).

***Paxillus involutus* (Pi)**
Project/s:
L'Institut National de la Recherche Agronomique: EctomycorrhizaDB (http://mycor.nancy.inra.fr/ectomycorrhizadb/)

***Sporobolomyces roseus IAM 13481* (Spr)**
Project/s:
DOE Joint Genome Institute: (http://genome.jgi-psf.org/euk_data.html)

***Ustilago maydis 521* (Um_a)**
Project/s:
Broad Institute of Harvard and MIT: Ustilago maydis Sequencing Project (http://www.broad.mit.edu/annotation/fungi/ustilago_maydis/)
National Center for Biotechnology Information: NCBI Fungi Genomes Project (http://www.ncbi.nlm.nih.gov/sutils/genom_table.cgi?organism=fungi)

***Ustilago maydis FB1* (Um_b)**
Project/s:
Broad Institute of Harvard and MIT: Ustilago maydis Database (http://www.broad.mit.edu/annotation/genome/ustilago_maydis/Home.html)

***Batrachochytrium dendrobatidis JEL423* (Bad)**
Project/s:
Broad Institute of Harvard and MIT: Batrachochytrium dendrobatidis Database (http://www.broad.mit.edu/annotation/genome/batrachochytrium_dendrobatidis)
TBestDB - Taxonomically Broad EST Database: Batrachochytrium dendrobatidis (http://tbestdb.bcm.umontreal.ca/searches/organism.php?orgID=BD)

***Encephalitozoon cuniculi GB-M1* (Ec)**
Project/s:
International Species Sequencing Consortium: Genoscope WGS project ()
National Center for Biotechnology Information: NCBI Fungi Genomes Project (http://www.ncbi.nlm.nih.gov/sutils/genom_table.cgi?organism=fungi)
Publication/s:
Katinka MD *et. al.* , *Nature* , **414** , 450 (2001).

***Antonospora locustae* (Anl)**
Project/s:
Marine Biological Laboratory at Woods Hole: Nosema locustae Genome Project (http://jbpc.mbl.edu/Nosema/)
TBestDB - Taxonomically Broad EST Database: Antonospora locustae (http://tbestdb.bcm.umontreal.ca/searches/organism.php?orgID=NL)

***Phycomyces blakesleeanus* (Phb)**
Project/s:
DOE Joint Genome Institute: (http://genome.jgi-psf.org/euk_data.html)

***Rhizopus arrhizus RA 99-880* (Rha)**
Project/s:
Broad Institute of Harvard and MIT: Rhizopus oryzae Database (http://www.broad.mit.edu/annotation/fungi/rhizopus_oryzae/)
National Center for Biotechnology Information: NCBI Fungi Genomes Project (http://www.ncbi.nlm.nih.gov/sutils/genom_table.cgi?organism=fungi)
TBestDB - Taxonomically Broad EST Database: Rhizopus oryzae
(http://amoebidia.bcm.umontreal.ca/public/pepdb/est.php?Info=est&orgID=RO&orgName=Rhizopus%20oryzae)

***Schistosoma japonicum* (Shj)**
Project/s:
GenBank - NIH genetic sequence database: (http://www.ncbi.nlm.nih.gov/BLAST/)
Publication/s:
Hu W *et. al.* , *Nat Genet* , **35** , 139 (2003).

***Schistosoma mansoni* (Sm)**
Project/s:
GenBank - NIH genetic sequence database: (http://www.ncbi.nlm.nih.gov/BLAST/)
The Institute for Genomic Research: TIGR Schistosoma mansoni Gene Index (http://www.tigr.org/tigr-scripts/tgi/T_index.cgi?species=s_mansoni)
The Institute for Genomic Research: The TIGR Schistosoma mansoni Genome Project (http://www.tigr.org/tdb/e2k1/sma1/)
Publication/s:
Dillon GP *et. al.* , *Int J Parasitol* , **36** , 1 (2006).
Verjovski-Almeida S *et. al.* , *Nat Genet* , **35** , 148 (2003).

***Convoluta convoluta* (Coc)**

***Paratomella rubra* (Pr)**

***Symsagittifera roscofensis* (Syr)**

***Nemertoderma westbladi* (Nw)**

***Discocelis tigrina* (Dit)**

***Thysanozoon sp. IRT-2002* (Ts)**

***Dugesia japonica* (Dj)**
Project/s:
GenBank - NIH genetic sequence database: (http://www.ncbi.nlm.nih.gov/BLAST/)
Publication/s:
Mineta K *et. al.* , *Proc Natl Acad Sci U S A* , **100** , 7666 (2003).

***Girardia tigrina* (Git)**

***Schmidtea mediterranea* (Scm)**
Project/s:
GenBank - NIH genetic sequence database: (http://www.ncbi.nlm.nih.gov/BLAST/)
The Genome Sequencing Center at Washington University: Schmidtea mediterranea (http://genome.wustl.edu/genome.cgi?GENOME=Schmidtea%20mediterranea)
Publication/s:
Zayas RM *et. al.* , *Proc Natl Acad Sci U S A* , **102** , 18491 (2005).

***Seriola dumerili* (Sd)**

***Ptyochromis sp. 'redtail sheller'* (Psrs)**

***Notothenia coriiceps* (Noc)**

***Paracirrhites forsteri* (Paf)**

***Dicentrarchus labrax* (Dil)**

***Morone saxatilis* (Mos)**

***Pennahia argentata* (Pa)**

***Siniperca chuatsi* (Sic)**

***Oryzias latipes str. Hd-rR* (Ol)**
Project/s:
GenBank - NIH genetic sequence database: (http://www.ncbi.nlm.nih.gov/BLAST/)
National Center for Biotechnology Information: NCBI Eukaryotic Genomes Project (http://www.ncbi.nlm.nih.gov/sutils/genom_table.cgi?organism=eukaryote)
NIG DNA Sequencing Center: Medaka Genome Sequencing Project (http://dolphin.lab.nig.ac.jp/medaka/)
The Institute for Genomic Research: TIGR Oryzias latipes Gene Index (http://www.tigr.org/tigr-scripts/tgi/T_index.cgi?species=o_latipes)
Publication/s:
Kimura T *et. al.* , *Mech Dev* , **121** , 915 (2004).

***Fundulus heteroclitus* (Fh)**
Project/s:
FunnyBase Expressed Gene Database: Annotated Fundulus Heteroclitus EST Gene Expression Database (http://genomics.rsmas.miami.edu/funnybase/super_craw4/)
The Institute for Genomic Research: TIGR Fundulus heteroclitus Gene Index (http://www.tigr.org/tigr-scripts/tgi/T_index.cgi?species=killifish)
The Marine Genomics Project: Fundulus species
(http://www.marinegenomics.org/speciesentry.php?&s=y&organism=est_f_species&abrev=F.%20species&srn=Fundulus%20species)
Publication/s:
Paschall JE *et. al.* , *BMC Genomics* , **5** , 96 (2004).

***Gasterosteus aculeatus* (Ga)**
Project/s:
GenBank - NIH genetic sequence database: (http://www.ncbi.nlm.nih.gov/BLAST/)

***Takifugu rubripes* (Tar)**
Project/s:
DOE Joint Genome Institute: Fugu rubripes (http://genome.jgi-psf.org/Takru4/Takru4.home.html)
e! Ensembl: Fugu (http://www.ensembl.org/Fugu_rubripes/index.html)
GenBank - NIH genetic sequence database: (http://www.ncbi.nlm.nih.gov/BLAST/)
International Species Sequencing Consortium: Takifugu rubripes Sequencing Consortium ()
National Center for Biotechnology Information: NCBI Eukaryotic Genomes Project (http://www.ncbi.nlm.nih.gov/sutils/genom_table.cgi?organism=eukaryotes)
School of Biological & Chemical Sciences: The Fugu Genomics Project (http://fugu.biology.qmul.ac.uk/)
Singapore Institute of Molecular and Cell Biology: Fugu Genome Project (http://www.fugu-sg.org/)
The Institute for Genomic Research: TIGR Takifugu Gene Index (http://www.tigr.org/tigr-scripts/tgi/T_index.cgi?species=fugu)
UCSC Genome Bioinformatics: Takifugu rubripes Genome Browser Gateway
(http://genome.cse.ucsc.edu/cgi-bin/hgGateway?clade=vertebrate&org=Fugu&db=0&hgsid=68833987)
Publication/s:
Clark MS *et. al.* , *Genome Res* , **13** , 2747 (2003).
Aparicio S *et. al.* , *Science* , **297** , 1301 (2002).

***Tetraodon nigroviridis* (Tn)**
Project/s:
Broad Institute of Harvard and MIT: Tetraodon nigroviridis Database (http://www.broad.mit.edu/annotation/tetraodon/)
e! Ensembl: Tetraodon (http://www.ensembl.org/Tetraodon_nigroviridis/index.html)
Genoscope: Tetraodon nigroviridis - A fish with a compact genome (http://www.genoscope.cns.fr/externe/English/Projets/Projet_C/organisme_C.html)
International Species Sequencing Consortium: Tetraodon nigroviridis Sequencing Consortium ()
National Center for Biotechnology Information: NCBI Eukaryotic Genomes Project (http://www.ncbi.nlm.nih.gov/sutils/genom_table.cgi?organism=eukaryote)
UCSC Genome Bioinformatics: Tetraodon nigroviridis Genome Browser Gateway
(http://genome.cse.ucsc.edu/cgi-bin/hgGateway?clade=vertebrate&org=Tetraodon&db=0&hgsid=68833987)
Publication/s:
Jaillon O *et. al.* , *Nature* , **431** , 946 (2004).

***Gadus morhua* (Gm)**

***Theragra chalcogramma* (Tc)**

***Oncorhynchus keta* (Ok)**

***Oncorhynchus mykiss* (Om)**
Project/s:
GenBank - NIH genetic sequence database: (http://www.ncbi.nlm.nih.gov/BLAST/)
The Institute for Genomic Research: TIGR Oncorhynchus mykiss Gene Index (http://www.tigr.org/tigr-scripts/tgi/T_index.cgi?species=r_trout)
Publication/s:
Rise ML *et. al.* , *Genome Res* , **14** , 478 (2004).
Rexroad CE 3rd *et. al.* , *Cytogenet Genome Res* , **102** , 347 (2003).

***Salmo salar* (Sas)**
Project/s:
GenBank - NIH genetic sequence database: (http://www.ncbi.nlm.nih.gov/BLAST/)
The Institute for Genomic Research: TIGR Salmo salar Gene Index (http://www.tigr.org/tigr-scripts/tgi/T_index.cgi?species=salmon)
Publication/s:
Rise ML *et. al.* , *Genome Res* , **14** , 478 (2004).

***Cyprinus carpio* (Cyc)**
Project/s:
GenBank - NIH genetic sequence database: (http://www.ncbi.nlm.nih.gov/BLAST/)
Publication/s:
Gracey AY *et. al.* , *Proc Natl Acad Sci U S A* , **101** , 16970 (2004).

***Pimephales promelas* (Pip)**

***Brachydanio rerio* (Br)**
Project/s:
e! Ensembl: Zebrafish (http://www.ensembl.org/Danio_rerio/index.html)
GenBank - NIH genetic sequence database: (http://www.ncbi.nlm.nih.gov/BLAST/)
National Center for Biotechnology Information: Zebrafish Gene Collection (http://zgc.nci.nih.gov/)
National Center for Biotechnology Information: Zebrafish Sequencing Project (http://www.ncbi.nlm.nih.gov/genome/seq/BlastGen/BlastGen.cgi?taxid=7955)
The Institute for Genomic Research: TIGR Zebrafish (Danio rerio) Gene Index (http://www.tigr.org/tigr-scripts/tgi/T_index.cgi?species=zfish)
UCSC Genome Bioinformatics: Danio rerio Genome Browser Gateway
(http://genome.cse.ucsc.edu/cgi-bin/hgGateway?clade=vertebrate&org=Zebrafish&db=0&hgsid=68833987)
ZFIN The Zebrafish Information Network: (http://zfin.org/cgi-bin/webdriver?MIval=aa-ZDB_home.apg)
Publication/s:
Gerhard DS *et. al.* , *Genome Res* , **14** , 2121 (2004).
Lo J *et. al.* , *Genome Res* , **13** , 455 (2003).

Strausberg RL *et. al.* , *Proc Natl Acad Sci U S A* , **99** , 16899 (2002).

***Ictalurus punctatus* (Ip)**
Project/s:
GenBank - NIH genetic sequence database: (http://www.ncbi.nlm.nih.gov/BLAST/)
The Institute for Genomic Research: TIGR Ictalurus punctatus Gene Index (http://www.tigr.org/tigr-scripts/tgi/T_index.cgi?species=catfish)
Publication/s:
Ju Z *et. al.* , *Gene* , **261** , 373 (2000).

***Ornithorhynchus anatinus* (Ora)**
Project/s:
National Center for Biotechnology Information: NCBI Eukaryotic Genomes Project (http://www.ncbi.nlm.nih.gov/sutils/genom_table.cgi?organism=eukaryote)
The Genome Sequencing Center at Washington University: Ornithorhynchus anatinus (http://genome.wustl.edu/genome.cgi?GENOME=Ornithorhynchus%20anatinus&GROUP=2)

***Echinops telfairi* (Ect)**
Project/s:
e! Ensembl: Lesser Hedgehog Tenrec (http://ensembl.org/Echinops_telfairi/index.html)
National Center for Biotechnology Information: NCBI Eukaryotic Genomes Project (http://www.ncbi.nlm.nih.gov/sutils/genom_table.cgi?organism=eukaryote)

***Oryctolagus cuniculus* (Oc)**
Project/s:
e! Ensembl: Rabbit (http://www.ensembl.org/Oryctolagus_cuniculus/index.html)
National Center for Biotechnology Information: NCBI Eukaryotic Genomes Project (http://www.ncbi.nlm.nih.gov/sutils/genom_table.cgi?organism=eukaryote)

***Mesocricetus auratus* (Ma)**

***Mus musculus* (Mm)**
Project/s:
Broad Institute of Harvard and MIT: Mouse Genome Data (http://www.broad.mit.edu/mouse/)
e! Ensembl: Mouse (http://www.ensembl.org/Mus_musculus/index.html)
GenBank - NIH genetic sequence database: (http://www.ncbi.nlm.nih.gov/BLAST/)
Human Genome Sequencing Center at Baylor College of Medicine: Mouse Genome Project (http://www.hgsc.bcm.tmc.edu/projects/mouse/)
International Species Sequencing Consortium: Mus musculus Sequencing Consortium ()
National Center for Biotechnology Information: Mammalian Gene Collection - Mus musculus (http://mgc.nci.nih.gov/)
National Center for Biotechnology Information: Mouse Sequencing Project (http://www.ncbi.nlm.nih.gov/genome/seq/BlastGen/BlastGen.cgi?taxid=10090)
The Genome Sequencing Center at Washington University: Mus musculus (http://genome.wustl.edu/genome.cgi?GENOME=Mus%20musculus&GROUP=2)
The Institute for Genomic Research: TIGR Mouse (Mus musculus) Gene Index (http://www.tigr.org/tigr-scripts/tgi/T_index.cgi?species=mouse)
The Jackson Laboratory: Mouse Genome Informatics (http://www.informatics.jax.org/)
UCSC Genome Bioinformatics: Mus musculus Genome Browser Gateway (http://genome.cse.ucsc.edu/cgi-bin/hgGateway?clade=vertebrate&org=Mouse&db=0&hgsid=68833987)
Publication/s:
Carninci P *et. al.* , *Science* , **309** , 1559 (2005).
Gerhard DS *et. al.* , *Genome Res* , **14** , 2121 (2004).
Okazaki Y *et. al.* , *Nature* , **420** , 563 (2002).
Strausberg RL *et. al.* , *Proc Natl Acad Sci U S A* , **99** , 16899 (2002).
Waterston RH *et. al.* , *Nature* , **420** , 520 (2002).
Kawai J *et. al.* , *Nature* , **409** , 685 (2001).

***Rattus norvegicus* (Rn)**
Project/s:
Bioinformatics Program, HMGC: Rat Genome Database (http://rgd.mcw.edu/)
e! Ensembl: Rat (http://www.ensembl.org/Rattus_norvegicus/index.html)
Human Genome Sequencing Center at Baylor College of Medicine: Rat Genome Project (http://www.hgsc.bcm.tmc.edu/projects/rat/)
International Species Sequencing Consortium: Rattus norvegicus Sequencing Consortium ()
National Center for Biotechnology Information: Mammalian Gene Collection - Rattus norvegicus (http://mgc.nci.nih.gov/)
National Center for Biotechnology Information: Rat Sequencing Project (http://www.ncbi.nlm.nih.gov/genome/seq/BlastGen/BlastGen.cgi?taxid=10116)
The Institute for Genomic Research: TIGR Rat (Rattus norvegicus) Gene Index (http://www.tigr.org/tigr-scripts/tgi/T_index.cgi?species=rat)
UCSC Genome Bioinformatics: Rattus norvegicus Genome Browser Gateway (http://genome.cse.ucsc.edu/cgi-bin/hgGateway?clade=vertebrate&org=Rat&db=0&hgsid=68833987)
Publication/s:
Gibbs RA *et. al.* , *Nature* , **428** , 493 (2004).
Gerhard DS *et. al.* , *Genome Res* , **14** , 2121 (2004).
Strausberg RL *et. al.* , *Proc Natl Acad Sci U S A* , **99** , 16899 (2002).

***Spermophilus tridecemlineatus* (Spt)**
Project/s:
National Center for Biotechnology Information: NCBI Eukaryotic Genomes Project (http://www.ncbi.nlm.nih.gov/sutils/genom_table.cgi?organism=eukaryote)

***Macaca fascicularis* (Mf)**
Project/s:
GenBank - NIH genetic sequence database: (http://www.ncbi.nlm.nih.gov/BLAST/)
Publication/s:
Magness CL *et. al.* , *Genome Biol* , **6** , R60 (2005).
Osada N *et. al.* , *Mol Biol Evol* , **22** , 1976 (2005).

***Macaca mulatta* (Mam)**
Project/s:
e! Ensembl: Rhesus macaque (http://www.ensembl.org/Macaca_mulatta/index.html)
GenBank - NIH genetic sequence database: (http://www.ncbi.nlm.nih.gov/BLAST/)
Human Genome Sequencing Center at Baylor College of Medicine: Rhesus Monkey Genome Project (http://www.hgsc.bcm.tmc.edu/projects/rmacaque/)
National Center for Biotechnology Information: NCBI Eukaryotic Genomes Project (http://www.ncbi.nlm.nih.gov/sutils/genom_table.cgi)
The Genome Sequencing Center at Washington University: Macaca mulatta (http://genome.wustl.edu/genome.cgi?GENOME=Macaca%20mulatta&GROUP=1)
UCSC Genome Bioinformatics: Macaca mulatta Genome Browser Gateway (http://genome.cse.ucsc.edu/cgi-bin/hgGateway?clade=vertebrate&org=Rhesus&db=0&hgsid=68833987)
Publication/s:
Magness CL *et. al.* , *Genome Biol* , **6** , R60 (2005).

***Homo sapiens* (Hs)**
Project/s:
e! Ensembl: Human (http://www.ensembl.org/Homo_sapiens/index.html)
GenBank - NIH genetic sequence database: (http://www.ncbi.nlm.nih.gov/BLAST/)
International Species Sequencing Consortium: Homo sapiens Sequencing Consortium ()
National Center for Biotechnology Information: Human Sequencing Project (http://www.ncbi.nlm.nih.gov/genome/seq/BlastGen/BlastGen.cgi?taxid=9606)
National Center for Biotechnology Information: Mammalian Gene Collection - Homo sapiens (http://mgc.nci.nih.gov/)
The Genome Sequencing Center at Washington University: Homo sapiens (http://genome.wustl.edu/genome.cgi?GENOME=Homo%20sapiens&GROUP=1)
The Institute for Genomic Research: TIGR Human (Homo sapiens) Gene Index (http://www.tigr.org/tigr-scripts/tgi/T_index.cgi?species=human)
UCSC Genome Bioinformatics: Homo sapiens Genome Browser Gateway (http://genome.cse.ucsc.edu/cgi-bin/hgGateway?clade=vertebrate&org=0&db=0&hgsid=68833987)
Publication/s:
Muzny DM *et. al.* , *Nature* , **440** , 1194 (2006).
Zody MC *et. al.* , *Nature* , **440** , 671 (2006).
Gregory SG *et. al.* , *Nature* , **441** , 315 (2006).
Taylor TD *et. al.* , *Nature* , **440** , 497 (2006).
Zody MC *et. al.* , *Nature* , **440** , 1045 (2006).

Nusbaum C *et. al.* , *Nature* , **439** , 331 (2006).
Kimura K *et. al.* , *Genome Res* , **16** , 55 (2006).
Scherer SE *et. al.* , *Nature* , **440** , 346 (2006).
Hillier LW *et. al.* , *Nature* , **434** , 724 (2005).
Nusbaum C *et. al.* , *Nature* , **437** , 551 (2005).
Ross MT *et. al.* , *Nature* , **434** , 325 (2005).
Martin J *et. al.* , *Nature* , **432** , 988 (2004).
Imanishi T *et. al.* , *PLoS Biol* , **2** , e162 (2004).
Ota T *et. al.* , *Nat Genet* , **36** , 40 (2004).
Grimwood J *et. al.* , *Nature* , **428** , 529 (2004).
Collins JE *et. al.* , *Genome Biol* , **5** , R84 (2004).
Rual JF *et. al.* , *Genome Res* , **14** , 2128 (2004).
Dunham A *et. al.* , *Nature* , **428** , 522 (2004).
Deloukas P *et. al.* , *Nature* , **429** , 375 (2004).
Humphray SJ *et. al.* , *Nature* , **429** , 369 (2004).
Brandenberger R *et. al.* , *Nat Biotechnol* , **22** , 707 (2004).
Schmutz J *et. al.* , *Nature* , **431** , 268 (2004).
Gerhard DS *et. al.* , *Genome Res* , **14** , 2121 (2004).
Heilig R *et. al.* , *Nature* , **421** , 601 (2003).
Hillier LW *et. al.* , *Nature* , **424** , 157 (2003).
Mungall AJ *et. al.* , *Nature* , **425** , 805 (2003).
Strausberg RL *et. al.* , *Proc Natl Acad Sci U S A* , **99** , 16899 (2002).
Lander ES *et. al.* , *Nature* , **409** , 860 (2001).
Deloukas P *et. al.* , *Nature* , **414** , 865 (2001).
Venter JC *et. al.* , *Science* , **291** , 1304 (2001).
Hattori M *et. al.* , *Nature* , **405** , 311 (2000).
Dias Neto E *et. al.* , *Proc Natl Acad Sci U S A* , **97** , 3491 (2000).
Dunham I *et. al.* , *Nature* , **402** , 489 (1999).

***Pan troglodytes* (Pat)**
Project/s:
e! Ensembl: Chimp (http://www.ensembl.org/Pan_troglodytes/index.html)
GenBank - NIH genetic sequence database: (http://www.ncbi.nlm.nih.gov/BLAST/)
Human Genome Sequencing Center at Baylor College of Medicine: Chimpanzee Genome Analysis (http://www.hgsc.bcm.tmc.edu/projects/chimpanzee/)
International Species Sequencing Consortium: Pan troglodytes Sequencing Consortium ()
National Center for Biotechnology Information: Chimpanzee Sequencing Project (http://www.ncbi.nlm.nih.gov/genome/seq/BlastGen/BlastGen.cgi?taxid=9598)
The Genome Sequencing Center at Washington University: Pan troglodytes (http://genome.wustl.edu/genome.cgi?GENOME=Pan%20troglodytes&GROUP=1)
UCSC Genome Bioinformatics: Pan troglodytes Genome Browser Gateway
(http://genome.cse.ucsc.edu/cgi-bin/hgGateway?clade=vertebrate&org=Chimp&db=0&hgsid=68833987)
Publication/s:
Watanabe H *et. al.* , *Nature* , **429** , 382 (2004).
Hellmann I *et. al.* , *Genome Res* , **13** , 831 (2003).
Sakate R *et. al.* , *Genome Res* , **13** , 1022 (2003).

***Pongo pygmaeus* (Ppy)**
Project/s:
National Center for Biotechnology Information: Pongo pygmaeus (http://www.ncbi.nlm.nih.gov/sutils/genom_table.cgi?organism=euk)

***Otolemur garnettii* (Otg)**
Project/s:
National Center for Biotechnology Information: NCBI Eukaryotic Genomes Project (http://www.ncbi.nlm.nih.gov/sutils/genom_table.cgi?organism=eukaryote)

***Canis familiaris* (Caf)**
Project/s:
e! Ensembl: Dog (http://www.ensembl.org/Canis_familiaris/index.html)
International Species Sequencing Consortium: Canis familiaris Sequencing Consortium ()
National Center for Biotechnology Information: Dog Sequencing Project (http://www.ncbi.nlm.nih.gov/genome/seq/BlastGen/BlastGen.cgi?taxid=9615)
The Institute for Genomic Research: TIGR Canis familiaris Gene Index (http://www.tigr.org/tigr-scripts/tgi/T_index.cgi?species=dog)
UCSC Genome Bioinformatics: Canis familiaris Genome Browser Gateway
(http://genome.cse.ucsc.edu/cgi-bin/hgGateway?clade=vertebrate&org=Dog&db=0&hgsid=68833987)
Publication/s:
Lindblad-Toh K *et. al.* , *Nature* , **438** , 803 (2005).

***Felis catus* (Fc)**
Project/s:
National Center for Biotechnology Information: Cat Sequencing Project (http://www.ncbi.nlm.nih.gov/genome/seq/BlastGen/BlastGen.cgi?taxid=9685)

***Bos taurus* (Bt)**
Project/s:
e! Ensembl: Cow (http://www.ensembl.org/Bos_taurus/index.html)
GenBank - NIH genetic sequence database: (http://www.ncbi.nlm.nih.gov/BLAST/)
Human Genome Sequencing Center at Baylor College of Medicine: Bovine Genome Project (http://www.hgsc.bcm.tmc.edu/projects/bovine/)
National Center for Biotechnology Information: Cow Sequencing Project (http://www.ncbi.nlm.nih.gov/genome/seq/BlastGen/BlastGen.cgi?taxid=9913)
National Center for Biotechnology Information: Mammalian Gene Collection - Bos taurus (http://mgc.nci.nih.gov/)
The Institute for Genomic Research: TIGR Cattle (Bos taurus) Gene Index (http://www.tigr.org/tigr-scripts/tgi/T_index.cgi?species=cattle)
UCSC Genome Bioinformatics: Bos taurus Genome Browser Gateway (http://genome.cse.ucsc.edu/cgi-bin/hgGateway?clade=vertebrate&org=Cow&db=0&hgsid=68833987)
Publication/s:
Sonstegard TS *et. al.* , *Mamm Genome* , **13** , 373 (2002).
Takasuga A *et. al.* , *Nucleic Acids Res* , **29** , E108 (2001).
Smith TP *et. al.* , *Genome Res* , **11** , 626 (2001).

***Sus scrofa domestica* (Ss)**
Project/s:
GenBank - NIH genetic sequence database: (http://www.ncbi.nlm.nih.gov/BLAST/)
National Center for Biotechnology Information: Pig Sequencing Project (http://www.ncbi.nlm.nih.gov/genome/seq/BlastGen/BlastGen.cgi?taxid=9823)
PEDE: Pig Expression Data Explorer (http://pede.dna.affrc.go.jp/)
The Institute for Genomic Research: TIGR Porcine (Sus scrofa) Gene Index (http://www.tigr.org/tigr-scripts/tgi/T_index.cgi?species=pig)
Publication/s:
Uenishi H *et. al.* , *Nucleic Acids Res* , **32** , D484 (2004).
Fahrenkrug SC *et. al.* , *Mamm Genome* , **13** , 475 (2002).
Davoli R *et. al.* , *Anim Genet* , **33** , 3 (2002).

***Carollia perspicillata* (Clp)**

***Myotis lucifugus* (Myl)**
Project/s:
National Center for Biotechnology Information: NCBI Eukaryotic Genomes Project (http://www.ncbi.nlm.nih.gov/sutils/genom_table.cgi?organism=eukaryote)

***Equus caballus* (Eqc)**

***Dasypus novemcinctus* (Dn)**
Project/s:
e! Ensembl: Armadillo (http://www.ensembl.org/Dasypus_novemcinctus/index.html)
National Center for Biotechnology Information: NCBI Eukaryotic Genomes Project (http://www.ncbi.nlm.nih.gov/sutils/genom_table.cgi?organism=eukaryote)

***Monodelphis domestica* (Md)**
Project/s:
Broad Institute of Harvard and MIT: Opossum Genome (http://www.broad.mit.edu/mammals/opossum/)
e! Ensembl: Opossum (http://www.ensembl.org/Monodelphis_domestica/index.html)
National Center for Biotechnology Information: NCBI Eukaryotic Genomes Project (http://www.ncbi.nlm.nih.gov/sutils/genom_table.cgi?organism=eukaryote)
UCSC Genome Bioinformatics: Monodelphis domestica Genome Browser Gateway
(http://genome.cse.ucsc.edu/cgi-bin/hgGateway?clade=vertebrate&org=Opossum&db=0&hgsid=68833987)

***Trichosurus vulpecula* (Tcv)**

***Coturnix coturnix* (Ctc)**

***Gallus gallus* (Gg)**
Project/s:
ChickEST Database: Gallus gallus EST database (http://www.chick.umist.ac.uk/)
e! Ensembl: Chicken (http://www.ensembl.org/Gallus_gallus/index.html)
GenBank - NIH genetic sequence database: (http://www.ncbi.nlm.nih.gov/BLAST/)
International Species Sequencing Consortium: Gallus gallus Sequencing Consortium ()
National Center for Biotechnology Information: Chicken Sequencing Project (http://www.ncbi.nlm.nih.gov/genome/seq/BlastGen/BlastGen.cgi?taxid=9031)
The Genome Sequencing Center at Washington University: Gallus gallus (http://genome.wustl.edu/genome.cgi?GENOME=Gallus%20gallus&GROUP=2)
The Institute for Genomic Research: TIGR Gallus gallus Gene Index (http://www.tigr.org/tigr-scripts/tgi/T_index.cgi?species=g_gallus)
UCSC Genome Bioinformatics: Gallus gallus Genome Browser Gateway
(http://genome.cse.ucsc.edu/cgi-bin/hgGateway?clade=vertebrate&org=Chicken&db=0&hgsid=68833987)
Publication/s:
Shin JH *et. al.* , *Anim Genet* , **37** , 85 (2006).
Shin JH *et. al.* , *Anim Genet* , **36** , 346 (2005).
Hubbard SJ *et. al.* , *Genome Res* , **15** , 174 (2005).
Caldwell RB *et. al.* , *Genome Biol* , **6** , R6 (2005).
Savolainen P *et. al.* , *Cytogenet Genome Res* , **111** , 79 (2005).
Hillier LW *et. al.* , *Nature* , **432** , 695 (2004).
Boardman PE *et. al.* , *Curr Bio* , **12** , 1965 (2002).

***Xenopus tropicalis* (Xt)**
Project/s:
DOE Joint Genome Institute: Xenopus tropicalis (http://genome.jgi-psf.org/Xentr4/Xentr4.home.html)
e! Ensembl: X.tropicalis (http://www.ensembl.org/Xenopus_tropicalis/index.html)
National Center for Biotechnology Information: Xenopus Gene Collection (http://xgc.nci.nih.gov/)
The Genome Sequencing Center at Washington University: Xenopus tropicalis (http://genome.wustl.edu/genome.cgi?GENOME=Xenopus%20tropicalis&GROUP=2)
The Institute for Genomic Research: TIGR Xenopus tropicalis Gene Index (http://www.tigr.org/tigr-scripts/tgi/T_index.cgi?species=x_tropicalis)
UCSC Genome Bioinformatics: Xenopus tropicalis Genome Browser Gateway
(http://genome.cse.ucsc.edu/cgi-bin/hgGateway?clade=vertebrate&org=X.+tropicalis&db=0&hgsid=68833987)
Xenbase: a Xenopus web resource: (http://www.xenbase.org/)
Publication/s:
Gerhard DS *et. al.* , *Genome Res* , **14** , 2121 (2004).
Strausberg RL *et. al.* , *Proc Natl Acad Sci U S A* , **99** , 16899 (2002).

***Xenopus laevis* (Xl)**
Project/s:
GenBank - NIH genetic sequence database: (http://www.ncbi.nlm.nih.gov/BLAST/)
National Center for Biotechnology Information: Xenopus Gene Collection (http://xgc.nci.nih.gov/)
The Institute for Genomic Research: TIGR African clawed frog (Xenopus laevis) Gene Index (http://www.tigr.org/tigr-scripts/tgi/T_index.cgi?species=xenopus)
Xenbase: a Xenopus web resource: (http://www.xenbase.org/)
Publication/s:
Pollet N *et. al.* , *Mech Dev* , **122** , 365 (2005).
Gerhard DS *et. al.* , *Genome Res* , **14** , 2121 (2004).
Strausberg RL *et. al.* , *Proc Natl Acad Sci U S A* , **99** , 16899 (2002).
Klein SL *et. al.* , *Dev Dyn* , **225** , 384 (2002).
Blackshear PJ *et. al.* , *Gene* , **267** , 71 (2001).

***Rana catesbeiana* (Rc)**

***Rana pipiens* (Rp)**

***Lethenteron japonicum* (Lj)**

***Oikopleura dioica* (Oid)**
Project/s:
GenBank - NIH genetic sequence database: (http://www.ncbi.nlm.nih.gov/BLAST/)
Publication/s:
Bassham S, Postlethwait JH , *Development* , **132** , 4259 (2005).

***Ciona intestinalis* (Ci)**
Project/s:
DOE Joint Genome Institute: Ciona intestinalis (http://genome.jgi-psf.org/ciona4/ciona4.home.html)
e! Ensembl: C.intestinalis (http://www.ensembl.org/Ciona_intestinalis/index.html)
Ghost Database: Ciona intestinalis genomic and cDNA resources (http://ghost.zool.kyoto-u.ac.jp/indexr1.html)
International Species Sequencing Consortium: Ciona intestinalis Sequencing Consortium ()
National Center for Biotechnology Information: NCBI Eukaryotic Genomes Project (http://www.ncbi.nlm.nih.gov/sutils/genom_table.cgi?organism=eukaryotes)
The Institute for Genomic Research: TIGR Ciona intestinalis Gene Index (http://www.tigr.org/tigr-scripts/tgi/T_index.cgi?species=c_intestinalis)
UCSC Genome Bioinformatics: Ciona intestinalias Genome Browser Gateway
(http://genome.cse.ucsc.edu/cgi-bin/hgGateway?clade=deuterostome&org=0&db=0&hgsid=68833987)
Publication/s:
Dehal P *et. al.* , *Science* , **298** , 2157 (2002).

***Ciona savignyi* (Cis)**
Project/s:
Broad Institute of Harvard and MIT: Ciona savignyi Database (http://www.broad.mit.edu/annotation/ciona/)
e! Ensembl: C.savignyi (http://ensembl.org/Ciona_savignyi/index.html)
National Center for Biotechnology Information: NCBI Eukaryotic Genomes Project (http://www.ncbi.nlm.nih.gov/sutils/genom_table.cgi?organism=eukaryote)

***Halocynthia roretzi* (Hr)**

***Strongylocentrotus purpuratus* (Stp)**
Project/s:
GenBank - NIH genetic sequence database: (http://www.ncbi.nlm.nih.gov/BLAST/)
Human Genome Sequencing Center at Baylor College of Medicine: Sea Urchin Genome Project (http://www.hgsc.bcm.tmc.edu/projects/seaurchin/)
International Species Sequencing Consortium: Strongylocentrotus purpuratus Sequencing Consortium ()
National Center for Biotechnology Information: NCBI Eukaryotic Genomes Project (http://www.ncbi.nlm.nih.gov/sutils/genom_table.cgi?organism=eukaryote)
UCSC Genome Bioinformatics: Strongylocentrotus purpuratus Genome Browser Gateway
(http://genome.cse.ucsc.edu/cgi-bin/hgGateway?clade=deuterostome&org=S.+purpuratus&db=0&hgsid=68833987)
Publication/s:
Sodergren E *et. al.* , *Science* , **314** , 941 (2006).
Poustka AJ *et. al.* , *Genome Res* , **13** , 2736 (2003).

***Erpobdellid sp. IRT-2002* (Ers)**

***Oligochaete sp. IRT-2002* (Ols)**

***Nereis sp. IRT-2002* (Ns)**

***Bonellia viridis* (Bv)**

***Phoronis hippocrepia* (Ph)**

***Brachiopod sp. IRT-2002* (Bs)**

***Spisula solidissima* (Sis)**

***Mytilus galloprovincialis* (Myg)**
Project/s:
GenBank - NIH genetic sequence database: (http://www.ncbi.nlm.nih.gov/BLAST/)
Publication/s:
Venier P *et. al.* , *Gene* , **314** , 29 (2003).

***Argopecten irradians* (Ai)**
Project/s:
The Marine Genomics Project: Argopecten irradians
(http://www.marinegenomics.org/speciesentry.php?&s=y&organism=est_a_irradians&abrev=A.%20irradians&srn=Argopecten%20irradians)

***Chlamys farreri* (Clf)**

***Mizuhopecten yessoensis* (My)**

***Pecten maximus* (Pem)**

***Placopecten magellanicus* (Pm)**

***Atrina rigida* (Atr)**

***Loligo pealeii* (Lp)**

***Ilyanassa obsoleta* (Io)**

***Aplysia californica* (Apc)**
Project/s:
National Center for Biotechnology Information: NCBI Eukaryotic Genomes Project (http://www.ncbi.nlm.nih.gov/sutils/genom_table.cgi)

***Lineus sp. IRT-2002* (Ls)**

***Rhipicephalus appendiculatus* (Ra)**
Project/s:
The Institute for Genomic Research: TIGR Rhipicephalus appendiculatus Gene Index (http://www.tigr.org/tigr-scripts/tgi/T_index.cgi?species=r_appendiculatus)
Publication/s:
Nene V *et. al.* , *Insect Biochem Mol Biol* , **34** , 1117 (2004).

***Cyrtophora citricola* (Cci)**

***Opiliones sp. IRT-2002* (Ops)**

***Euscorpius flavicaudis* (Ef)**

***Limulus polyphemus* (Lip)**

***Scutigera coleoptrata* (Sco)**

***Lithobius sp. IRT-2002* (Lis)**

***Daphnia pulex* (Dap)**
Project/s:
wFleaBase: Daphnia Water Flea Genome Database (http://wfleabase.org/)

***Homarus gammarus* (Hg)**

***Idotea resecata* (Ir)**

***Bombyx mori str. Dazao* (Bm)**
Project/s:
National Center for Biotechnology Information: NCBI Insect Genomes Project (http://www.ncbi.nlm.nih.gov/sutils/genom_table.cgi?organism=insects)
SilkBase: EST Database of the Silkworm, Bombyx mori (http://papilio.ab.a.u-tokyo.ac.jp/silkbase/index.html)
SilkDB: Silkworm Knowledgebase: Bombyx mori project (http://silkworm.genomics.org.cn/index.jsp)
The Silkworm Genome Research Program: Bombyx mori project (http://sgp.dna.affrc.go.jp/index.html)
Publication/s:
Mita K *et. al.* , *DNA Res* , **11** , 27 (2004).
Mita K *et. al.* , *Proc Natl Acad Sci U S A* , **100** , 14121 (2003).

***Tribolium castaneum str. Georgia GA2* (Tic)**
Project/s:
BeetleBase: Tribolium Genome Database (http://www.bioinformatics.ksu.edu/BeetleBase/)
Human Genome Sequencing Center at Baylor College of Medicine: Tribolium castaneum Genome Project (http://www.hgsc.bcm.tmc.edu/projects/tribolium/)
National Center for Biotechnology Information: NCBI Insect Genomes Project (http://www.ncbi.nlm.nih.gov/sutils/genom_table.cgi?organism=insects)

***Drosophila grimshawi TSC#15287-2541.00* (Dg)**
Project/s:
FlyBase: A Database of the Drosophila Genome (http://flybase.bio.indiana.edu/)
National Center for Biotechnology Information: NCBI Insect Genomes Project (http://www.ncbi.nlm.nih.gov/sutils/genom_table.cgi?organism=insects)
UCSC Genome Bioinformatics: Drosophila grimshawi Genome Browser Gateway
(http://genome.ucsc.edu/cgi-bin/hgGateway?clade=insect&org=D.+grimshawi&db=0&hgsid=63313315)

***Drosophila hydei* (Dh)**

***Drosophila mojavensis TSC#15081-1352.22* (Dmo)**
Project/s:
FlyBase: A Database of the Drosophila Genome (http://flybase.bio.indiana.edu/)
National Center for Biotechnology Information: NCBI Insect Genomes Project (http://www.ncbi.nlm.nih.gov/sutils/genom_table.cgi?organism=insects)
UCSC Genome Bioinformatics: Drosophila mojavensis Genome Browser Gateway (http://genome.cse.ucsc.edu/cgi-bin/hgGateway?clade=insect&org=D.+mojavensis)

***Drosophila virilis TSC#15010-1051.87* (Dv)**
Project/s:

FlyBase: A Database of the Drosophila Genome (http://flybase.bio.indiana.edu/)
National Center for Biotechnology Information: NCBI Insect Genomes Project (http://www.ncbi.nlm.nih.gov/sutils/genom_table.cgi?organism=insects)
UCSC Genome Bioinformatics: Drosophila virilis Genome Browser Gateway (http://genome.cse.ucsc.edu/cgi-bin/hgGateway?org=D.+virilis&db=droVir2)

Drosophila ananassae TSC#14024-0371.13 **(Da)**
Project/s:
FlyBase: A Database of the Drosophila Genome (http://flybase.bio.indiana.edu/)
National Center for Biotechnology Information: NCBI Insect Genomes Project (http://www.ncbi.nlm.nih.gov/sutils/genom_table.cgi?organism=insects)
UCSC Genome Bioinformatics: Drosophila ananassae Genome Browser Gateway (http://genome.cse.ucsc.edu/cgi-bin/hgGateway?clade=insect&org=D.+ananassae)

Drosophila yakuba Tai18E2 **(Dy)**
Project/s:
FlyBase: A Database of the Drosophila Genome (http://flybase.bio.indiana.edu/)
GenBank - NIH genetic sequence database: (http://www.ncbi.nlm.nih.gov/BLAST/)
National Center for Biotechnology Information: NCBI Insect Genomes Project (http://www.ncbi.nlm.nih.gov/sutils/genom_table.cgi?organism=insects)
The Genome Sequencing Center at Washington University: Drosophila yakuba Sequencing (http://genome.wustl.edu/genome.cgi?GENOME=Drosophila%20yakuba&GROUP=6)
UCSC Genome Bioinformatics: Drosophila yakuba Genome Browser Gateway
(http://genome.cse.ucsc.edu/cgi-bin/hgGateway?clade=insect&org=D.+yakuba&db=0&hgsid=60909374)
Publication/s:
Domazet-Loso T, Tautz D , *Genome Res* , **13** , 2213 (2003).

Drosophila melanogaster **(Dm)**
Project/s:
Berkeley Drosophila Genome Project: Berkeley Drosophila Genome Project (http://www.fruitfly.org/)
e! Ensembl: Fruitfly (http://www.ensembl.org/Drosophila_melanogaster/index.html)
FlyBase: A Database of the Drosophila Genome (http://flybase.bio.indiana.edu/)
GenBank - NIH genetic sequence database: (http://www.ncbi.nlm.nih.gov/BLAST/)
International Species Sequencing Consortium: Drosophila melanogaster Sequencing Consortium ()
National Center for Biotechnology Information: Drosophila melanogaster Sequencing Project (http://www.ncbi.nlm.nih.gov/genome/seq/BlastGen/BlastGen.cgi?taxid=7227)
The Institute for Genomic Research: TIGR Drosophila Gene Index (http://www.tigr.org/tigr-scripts/tgi/T_index.cgi?species=drosoph)
UCSC Genome Bioinformatics: Drosophila melanogaster Genome Browser Gateway
(http://genome.cse.ucsc.edu/cgi-bin/hgGateway?clade=insect&org=D.+melanogaster&db=0&hgsid=67179843)
Publication/s:
Celniker SE *et. al.* , *Genome Biol* , RESEARCH0079 (2002).
Misra S *et. al.* , *Genome Biol* , RESEARCH0083 (2002).
Andrews J *et. al.* , *Genome Res* , **10** , 2030 (2000).
Adams MD *et. al.* , *Science* , **287** , 2185 (2000).

Drosophila erecta TSC#14021-0224.01 **(Der)**
Project/s:
FlyBase: A Database of the Drosophila Genome (http://flybase.bio.indiana.edu/)
National Center for Biotechnology Information: NCBI Insect Genomes Project (http://www.ncbi.nlm.nih.gov/sutils/genom_table.cgi?organism=insects)
UCSC Genome Bioinformatics: Drosophila erecta Genome Browser Gateway (http://genome.cse.ucsc.edu/cgi-bin/hgGateway?clade=insect&org=D.+erecta)

Drosophila sechellia Rob3c **(Dse)**
Project/s:
FlyBase: A Database of the Drosophila Genome (http://flybase.bio.indiana.edu/)
National Center for Biotechnology Information: NCBI Insect Genomes Project (http://www.ncbi.nlm.nih.gov/sutils/genom_table.cgi?organism=insects)
UCSC Genome Bioinformatics: Drosophila sechellia Genome Browser Gateway
(http://genome.cse.ucsc.edu/cgi-bin/hgGateway?clade=insect&org=D.+sechellia&db=0&hgsid=67179843)

Drosophila simulans str. white501 **(Dss_a)**
Project/s:
FlyBase: A Database of the Drosophila Genome (http://flybase.bio.indiana.edu/)
National Center for Biotechnology Information: NCBI Insect Genomes Project (http://www.ncbi.nlm.nih.gov/sutils/genom_table.cgi?organism=insects)
The Genome Sequencing Center at Washington University: Drosophila simulans Sequencing
(http://genome.wustl.edu/genome.cgi?GENOME=Drosophila%20simulans&GROUP=6)
UCSC Genome Bioinformatics: Drosophila simulans Genome Browser Gateway
(http://genome.cse.ucsc.edu/cgi-bin/hgGateway?clade=insect&org=D.+simulans&db=0&hgsid=60909439)

Drosophila persimilis MSH-3 **(Drp)**
Project/s:
FlyBase: A Database of the Drosophila Genome (http://flybase.bio.indiana.edu/)
National Center for Biotechnology Information: NCBI Insect Genomes Project (http://www.ncbi.nlm.nih.gov/sutils/genom_table.cgi?organism=insects)
UCSC Genome Bioinformatics: Drosophila persimilis Genome Browser Gateway
(http://genome.cse.ucsc.edu/cgi-bin/hgGateway?clade=insect&org=D.+persimilis&db=0&hgsid=67179843)

Drosophila pseudoobscura MV2-25 **(Dp)**
Project/s:
FlyBase: A Database of the Drosophila Genome (http://flybase.bio.indiana.edu/)
Human Genome Sequencing Center at Baylor College of Medicine: Drosophila Genome Project (http://www.hgsc.bcm.tmc.edu/projects/drosophila/)
International Species Sequencing Consortium: Drosophila pseudoobscura Sequencing Consortium ()
National Center for Biotechnology Information: NCBI Insect Genomes Project (http://www.ncbi.nlm.nih.gov/sutils/genom_table.cgi?organism=insects)
UCSC Genome Bioinformatics: Drosophila pseudoobscura Genome Browser Gateway
(http://genome.cse.ucsc.edu/cgi-bin/hgGateway?clade=insect&org=D.+pseudoobscura&db=0&hgsid=60909439)
Publication/s:
Richards S *et. al.* , *Genome Res* , **15** , 1 (2005).

Drosophila willistoni TSC#14030-0811.24 **(Dw)**
Project/s:
National Center for Biotechnology Information: NCBI Insect Genomes Project (http://www.ncbi.nlm.nih.gov/sutils/genom_table.cgi?organism=insects)

Glossina morsitans morsitans **(Gom)**
Project/s:
GenBank - NIH genetic sequence database: (http://www.ncbi.nlm.nih.gov/BLAST/)
The Wellcome Trust Sanger Institute: Glossina morsitans morsitans EST sequencing (http://www.sanger.ac.uk/Projects/G_morsitans/)
Publication/s:
Lehane MJ *et. al.* , *Genome Biol* , **4** , R63 (2003).

Anopheles gambiae str. PEST **(Ang)**
Project/s:
AnoBase - The Anopheles Database: AnoBase - The Anopheles Database (http://www.anobase.org/index.html)
e! Ensembl: Mosquito (http://www.ensembl.org/Anopheles_gambiae/index.html)
Genoscope: Anopheles gambiae (http://www.genoscope.cns.fr/externe/English/Projets/Projet_AK/organisme_AK.html)
International Species Sequencing Consortium: Anopheles gambiae Sequencing Consortium ()
National Center for Biotechnology Information: NCBI Insect Genomes Project (http://www.ncbi.nlm.nih.gov/sutils/genom_table.cgi?organism=insects)
The Institute for Genomic Research: TIGR Mosquito Gene Index (http://www.tigr.org/tigr-scripts/tgi/T_index.cgi?species=mosquito)
UCSC Genome Bioinformatics: Anopheles gambiae Genome Browser Gateway
(http://genome.cse.ucsc.edu/cgi-bin/hgGateway?clade=insect&org=A.+gambiae&db=0&hgsid=67179843)
VectorBase: A Bioinformatics Resource Center for Invertebrate Vectors of Human Pathogens (http://www.vectorbase.org/index.php)
Publication/s:
Holt RA *et. al.* , *Science* , **298** , 129 (2002).

Aedes aegypti str. Liverpool **(Aea)**

Project/s:
Broad Institute of Harvard and MIT: Aedes aegypti Database (http://www.broad.mit.edu/annotation/disease_vector/aedes_aegypti/)
e! Ensembl: A.aegypti (http://ensembl.org/Aedes_aegypti/index.html)
GenBank - NIH genetic sequence database: (http://www.ncbi.nlm.nih.gov/BLAST/)
National Center for Biotechnology Information: NCBI Insect Genomes Project (http://www.ncbi.nlm.nih.gov/sutils/genom_table.cgi?organism=insects)
The Institute for Genomic Research: TIGR A. aegypti Gene Index (http://www.tigr.org/tigr-scripts/tgi/T_index.cgi?species=a_aegypti)
VectorBase: A Bioinformatics Resource Center for Invertebrate Vectors of Human Pathogens (http://www.vectorbase.org/index.php)
Publication/s:
Bartholomay LC *et. al.* , *Infect Immun* , **72** , 4114 (2004).

***Armigeres subalbatus* (Ars)**

***Apis mellifera str. DH4* (Am)**
Project/s:
e! Ensembl: Honeybee (http://www.ensembl.org/Apis_mellifera/index.html)
GenBank - NIH genetic sequence database: (http://www.ncbi.nlm.nih.gov/BLAST/)
Human Genome Sequencing Center at Baylor College of Medicine: Honey Bee Genome Project (http://www.hgsc.bcm.tmc.edu/projects/honeybee/)
National Center for Biotechnology Information: Honey bee Sequencing Project (http://www.ncbi.nlm.nih.gov/genome/seq/BlastGen/BlastGen.cgi?taxid=7460)
The Institute for Genomic Research: TIGR Honeybee (Apis mellifera) Gene Index (http://www.tigr.org/tigr-scripts/tgi/T_index.cgi?species=honeybee)
UCSC Genome Bioinformatics: Apis mellifera Genome Browser Gateway
(http://genome.cse.ucsc.edu/cgi-bin/hgGateway?clade=insect&org=A.+mellifera&db=0&hgsid=67179843)
Publication/s:
Nunes FM *et. al.* , *BMC Genomics* , **5** , 84 (2004).
Whitfield CW *et. al.* , *Genome Res* , **12** , 555 (2002).

***Ammophila sp. IRT-2002* (Ams)**

***Nasonia vitripennis* (Nav)**
Project/s:
Human Genome Sequencing Center at Baylor College of Medicine: Nasonia Genome Project (http://www.hgsc.bcm.tmc.edu/projects/nasonia/)
University of Rochester: Nasonia Homepage (http://www.rochester.edu/College/BIO/labs/WerrenLab/nasonia/)

***Blatta sp. IRT-2002* (Bls)**

***Empusa sp. IRT-2002* (Es)**

***Lepisma saccharina* (Les)**

***Priapulus caudatus* (Prc)**

***Phascolosoma granulatum* (Pg)**

***Ascaris suum* (Ass)**
Project/s:
Nematode.net Genome Sequencing Center: Ascaris suum (http://nematode.net/Species.Summaries/Ascaris.suum/index.php)
Publication/s:
Parkinson J *et. al.* , *Nat Genet* , **36** , 1259 (2004).

***Toxocara canis* (Toc)**
Project/s:
Nematode.net Genome Sequencing Center: Toxocara canis (http://nematode.net/Species.Summaries/Toxocara.canis/index.php)
Publication/s:
Parkinson J *et. al.* , *Nat Genet* , **36** , 1259 (2004).

***Strongyloides stercoralis* (Sts)**
Project/s:
Nematode.net Genome Sequencing Center: Strongyloides stercoralis (http://nematode.net/Species.Summaries/Strongyloides.stercoralis/index.php)
Publication/s:
Parkinson J *et. al.* , *Nat Genet* , **36** , 1259 (2004).

***Strongyloides ratti* (Str)**
Project/s:
GenBank - NIH genetic sequence database: (http://www.ncbi.nlm.nih.gov/BLAST/)
Nematode.net Genome Sequencing Center: Strongyloides ratti (http://nematode.net/Species.Summaries/Strongyloides.ratti/index.php)
Publication/s:
Thompson FJ *et. al.* , *Mol Biochem Parasitol* , **142** , 32 (2005).
Parkinson J *et. al.* , *Nat Genet* , **36** , 1259 (2004).

***Caenorhabditis elegans* (Ce)**
Project/s:
e! Ensembl: C.elegans (http://www.ensembl.org/Caenorhabditis_elegans/index.html)
GenBank - NIH genetic sequence database: (http://www.ncbi.nlm.nih.gov/BLAST/)
International Species Sequencing Consortium: Caenorhabditis elegans Sequencing Consortium ()
National Center for Biotechnology Information: Caenorhabditis elegans / Caenorhabditis briggsae (http://www.ncbi.nlm.nih.gov/BLAST/Genome/NematodeBlast.html)
Nematode.net Genome Sequencing Center: Caenorhabditis elegans (http://nematode.net/Species.Summaries/Caenorhabditis.elegans/index.php)
The Genome Sequencing Center at Washington University: Caenorhabditis elegans (http://genome.wustl.edu/genome.cgi?GENOME=Caenorhabditis%20elegans&GROUP=6)
The Institute for Genomic Research: TIGR Caenorhabditis elegans Gene Index (http://www.tigr.org/tigr-scripts/tgi/T_index.cgi?species=elegans)
The Wellcome Trust Sanger Institute: Caenorhabditis Genome Sequencing Projects (http://www.sanger.ac.uk/Projects/C_elegans/)
UCSC Genome Bioinformatics: Caenorhabditis elegans Genome Browser Gateway
(http://genome.cse.ucsc.edu/cgi-bin/hgGateway?clade=worm&org=0&db=0&hgsid=68833987)
WorfDB: The C. elegans ORFeome cloning project (http://worfdb.dfci.harvard.edu/)
WormBase: WormBase (http://www.wormbase.org/)
Publication/s:
Li S *et. al.* , *Science* , **303** , 540 (2004).
Lamesch P *et. al.* , *Genome Res* , **14** , 2064 (2004).
Reboul J *et. al.* , *Nat Genet* , **34** , 35 (2003).
C. elegans Sequencing Consortium , *Science* , **282** , 2012 (1998).

***Caenorhabditis remanei PB4641* (Car)**
Project/s:
National Center for Biotechnology Information: NCBI Eukaryotic Genomes Project (http://www.ncbi.nlm.nih.gov/sutils/genom_table.cgi?organism=eukaryote)
The Genome Sequencing Center at Washington University: Caenorhabditis remanei (http://genome.wustl.edu/genome.cgi?GENOME=Caenorhabditis%20remanei&GROUP=6)

***Caenorhabditis briggsae* (Cb)**
Project/s:
International Species Sequencing Consortium: Caenorhabditis briggsae Sequencing Consortium ()
National Center for Biotechnology Information: Caenorhabditis elegans / Caenorhabditis briggsae (http://www.ncbi.nlm.nih.gov/BLAST/Genome/NematodeBlast.html)
Nematode.net Genome Sequencing Center: Caenorhabditis briggsae (http://nematode.net/Species.Summaries/Caenorhabditis.briggsae/index.php)
The Genome Sequencing Center at Washington University: Caenorhabditis briggsae (http://genome.wustl.edu/genome.cgi?GENOME=Caenorhabditis%20briggsae&GROUP=6)
The Wellcome Trust Sanger Institute: The Caenorhabditis briggsae Genome Project (http://www.sanger.ac.uk/Projects/C_briggsae/)
UCSC Genome Bioinformatics: Caenorhabditis briggsae Genome Browser Gateway
(http://genome.cse.ucsc.edu/cgi-bin/hgGateway?clade=worm&org=C.+briggsae&db=0&hgsid=68833987)
WormBase: WormBase (http://www.wormbase.org/)
Publication/s:

Stein LD *et. al.* , *PLoS Biol* , **1** , E45 (2003).

Haemonchus contortus **(Hc)**
Project/s:
Nematode.net Genome Sequencing Center: Haemonchus contortus (http://nematode.net/Species.Summaries/Haemonchus.contortus/index.php)
Publication/s:
Parkinson J *et. al.* , *Nat Genet* , **36** , 1259 (2004).

Ostertagia ostertagi **(Oo)**
Project/s:
Nematode.net Genome Sequencing Center: Ostertagia ostertagi (http://nematode.net/Species.Summaries/Ostertagia.ostertagi/index.php)
Publication/s:
Parkinson J *et. al.* , *Nat Genet* , **36** , 1259 (2004).

Teladorsagia circumcincta **(Tec)**
Project/s:
Nematode.net Genome Sequencing Center: Teladorsagia circumcincta (http://nematode.net/Species.Summaries/Teladorsagia.circumcincta/index.php)
Publication/s:
Parkinson J *et. al.* , *Nat Genet* , **36** , 1259 (2004).

Brugia malayi **(Brm)**
Project/s:
Nematode.net Genome Sequencing Center: Brugia malayi (http://nematode.net/Species.Summaries/Brugia.malayi/index.php)
The Institute for Genomic Research: Brugia malayi Genome Project (http://www.tigr.org/tdb/e2k1/bma1/)
The Institute for Genomic Research: TIGR Brugia malayi Gene Index (http://www.tigr.org/tigr-scripts/tgi/T_index.cgi?species=b_malayi)
Publication/s:
Parkinson J *et. al.* , *Nat Genet* , **36** , 1259 (2004).

Onchocerca volvulus **(Ov)**
Project/s:
GenBank - NIH genetic sequence database: (http://www.ncbi.nlm.nih.gov/BLAST/)
Nematode.net Genome Sequencing Center: Onchocerca volvulus & Onchocerca ochengi (http://nematode.net/Species.Summaries/Onchocerca.ochengi/index.php)
The Institute for Genomic Research: TIGR Onchocerca volvulus Gene Index (http://www.tigr.org/tigr-scripts/tgi/T_index.cgi?species=o_volvulus)
Publication/s:
Parkinson J *et. al.* , *Nat Genet* , **36** , 1259 (2004).
Lizotte-Waniewski M *et. al.* , *Infect Immun* , **68** , 3491 (2000).

Meloidogyne arenaria **(Mea)**
Project/s:
Nematode.net Genome Sequencing Center: Meloidogyne arenaria (http://nematode.net/Species.Summaries/Meloidogyne.arenaria/index.php)
Publication/s:
Parkinson J *et. al.* , *Nat Genet* , **36** , 1259 (2004).

Meloidogyne incognita **(Mi)**
Project/s:
Nematode.net Genome Sequencing Center: Meloidogyne incognita (http://nematode.net/Species.Summaries/Meloidogyne.incognita/index.php)
Publication/s:
Parkinson J *et. al.* , *Nat Genet* , **36** , 1259 (2004).

Meloidogyne chitwoodi **(Mlc)**
Project/s:
Nematode.net Genome Sequencing Center: Meloidogyne chitwoodi (http://nematode.net/Species.Summaries/Meloidogyne.chitwoodi/index.php)
Publication/s:
Parkinson J *et. al.* , *Nat Genet* , **36** , 1259 (2004).

Xiphinema index **(Xi)**
Project/s:
Nematode.net Genome Sequencing Center: Xiphinema index (http://nematode.net/Species.Summaries/Xiphinema.index/index.php)

Trichinella spiralis **(Trs)**
Project/s:
Nematode.net Genome Sequencing Center: Trichinella spiralis (http://nematode.net/Species.Summaries/Trichinella.spiralis/index.php)
The Genome Sequencing Center at Washington University: Trichinella spiralis (http://genome.wustl.edu/genome.cgi?GENOME=Trichinella%20spiralis)
Publication/s:
Parkinson J *et. al.* , *Nat Genet* , **36** , 1259 (2004).

Brachionus plicatilis **(Brp)**

Nematostella vectensis **(Nv)**
Project/s:
DOE Joint Genome Institute: Nematostella vectensis (http://genome.jgi-psf.org/Nemve1/Nemve1.home.html)
GenBank - NIH genetic sequence database: (http://www.ncbi.nlm.nih.gov/BLAST/)
StellaBase: Nematostella vectensis genomic database (http://www.stellabase.org/)
Publication/s:
Technau U *et. al.* , *Trends Genet* , **21** , 633 (2005).

Anemonia sulcata **(As)**

Bunodactis verrucosa **(Buv)**

Podocoryne carnea **(Poc)**

Hydra magnipapillata **(Hm)**

Naegleria gruberi NEG-M **(Ng)**
Project/s:
DOE Joint Genome Institute: Naegleria gruberi (http://genome.jgi-psf.org/Naegr1/Naegr1.home.html)

Amoeba proteus **(Amp)**

Hartmannella vermiformis **(Hav)**
Project/s:
TBestDB - Taxonomically Broad EST Database: Hartmannella vermiformis (http://tbestdb.bcm.umontreal.ca/searches/organism.php?orgID=HV)

Dictyostelium discoideum AX4 **(Dd)**
Project/s:
Baylor College of Medicine: Functional Genomics of Dictyostelium (http://dictygenome.bcm.tmc.edu/)
Dept. Genome Analysis, IMB Jena: Dictyostelium discoideum Genome Project (http://genome.imb-jena.de/dictyostelium/)
dictyBase: Dictyostelium genome information, curated Dictyostelium literature (http://dictybase.org/)
Dictyostelium cDNA Project: Dictyostelium cDNA Project (http://dictycdb.biol.tsukuba.ac.jp/cDNAproject.html)
International Species Sequencing Consortium: Dictyostelium discoideum Sequencing Consortium ()
National Center for Biotechnology Information: NCBI Protozoa genomes (http://www.ncbi.nlm.nih.gov/sutils/blast_table.cgi?taxid=Protozoa)
The Institute for Genomic Research: TIGR Dictyostelium discoideum Gene Index (http://www.tigr.org/tigr-scripts/tgi/T_index.cgi?species=d_discoideum)
The Wellcome Trust Sanger Institute: The Dictyostelium discoideum Genome Project (http://www.sanger.ac.uk/Projects/D_discoideum/)

Publication/s:
Eichinger L *et. al.* , *Nature* , **435** , 43 (2005).
Urushihara H *et. al.* , *Nucleic Acids Res* , **32** , 1647 (2004).
Glockner G *et. al.* , *Nature* , **418** , 79 (2002).
Morio T *et. al.* , *DNA Res* , **5** , 335 (1998).

***Physarum polycephalum* (Pp)**
Project/s:
TBestDB - Taxonomically Broad EST Database: Physarum polycephalum (http://tbestdb.bcm.umontreal.ca/searches/organism.php?orgID=PP)

***Galdieria sulphuraria* (Gs)**
Project/s:
Michigan State University: The Galdieria sulphararia Genome Project (http://genomics.msu.edu/galdieria/index.html)

***Chlamydomonas reinhardtii* (Cr)**
Project/s:
Chlamydomonas Center: Chlamydomonas reinhardtii resource (http://www.chlamy.org)
DOE Joint Genome Institute: Chlamydomonas reinhardtii (http://genome.jgi-psf.org/chlre2/chlre2.home.html)
GenBank - NIH genetic sequence database: (http://www.ncbi.nlm.nih.gov/BLAST/)
Kazusa DNA Research Institute: Chlamydomonas reinhardtii EST index (http://www.kazusa.or.jp/en/plant/chlamy/EST/)
The Institute for Genomic Research: TIGR Chlamydomonas reinhardtii Gene Index (http://www.tigr.org/tigr-scripts/tgi/T_index.cgi?species=c_reinhardtii)
Publication/s:
Asamizu E *et. al.* , *DNA Res* , **7** , 305 (2000).
Asamizu E *et. al.* , *DNA Res* , **7** , 305 (2000).

***Ostreococcus lucimarinus* (Osl)**
Project/s:
DOE Joint Genome Institute: Ostreococcus lucimarinus (http://genome.jgi-psf.org/Ost9901_3/Ost9901_3.home.html)

***Ostreococcus tauri* (Ot)**
Project/s:
Bioinformatics & Evolutionary Genomics: Ostreococcus tauri (http://bioinformatics.psb.ugent.be/genomes.php)
DOE Joint Genome Institute: Ostreococcus tauri (http://genome.jgi-psf.org/Ostta4/Ostta4.home.html)
International Species Sequencing Consortium: Ostreococcus tauri Sequencing Consortium ()
Publication/s:
Derelle E *et. al.* , *Proc Natl Acad Sci U S A* , **103** , 11647 (2006).

***Acetabularia peniculus* (Acp)**

***Chara corallina* (Chc)**

***Adiantum capillus-veneris* (Acv)**

***Anemia phyllitidis* (Ap)**

***Pinus taeda* (Pit)**

***Vallisneria gigantea* (Vg)**

***Allium cepa* (Alc)**
Project/s:
The Institute for Genomic Research: TIGR Allium cepa Gene Index (http://www.tigr.org/tigr-scripts/tgi/T_index.cgi?species=onion)

***Oryza sativa (indica cultivar-group)* (Os_b)**
Project/s:
GenBank - NIH genetic sequence database: (http://www.ncbi.nlm.nih.gov/BLAST/)
International Species Sequencing Consortium: Oryza sativa (indica cultivar-group) Sequencing Consortium ()
The Institute for Genomic Research: TIGR Rice (Oryza sativa) Gene Index (http://www.tigr.org/tigr-scripts/tgi/T_index.cgi?species=rice)
Publication/s:
Zhang J *et. al.* , *Plant J* , **42** , 772 (2005).
Xie K *et. al.* , *Sci China C Life Sci* , **48** , 445 (2005).
Yu J *et. al.* , *PLoS Biol* , **3** , e38 (2005).
Matsumoto T *et. al.* , *Nature* , **436** , 793 (2005).
Yu J *et. al.* , *Science* , **296** , 79 (2002).
Feng Q *et. al.* , *Nature* , **420** , 316 (2002).

***Oryza sativa (japonica cultivar-group)* (Os_a)**
Project/s:
GenBank - NIH genetic sequence database: (http://www.ncbi.nlm.nih.gov/BLAST/)
International Species Sequencing Consortium: Oryza sativa (japonica cultivar-group) Sequencing Consortium ()
The Institute for Genomic Research: TIGR Rice (Oryza sativa) Gene Index (http://www.tigr.org/tigr-scripts/tgi/T_index.cgi?species=rice)
Publication/s:
Jantasuriyarat C *et. al.* , *Plant Physiol* , **138** , 105 (2005).
Matsumoto T *et. al.* , *Nature* , **436** , 793 (2005).
Yu J *et. al.* , *PLoS Biol* , **3** , e38 (2005).
Kikuchi S *et. al.* , *Science* , **301** , 376 (2003).
Goff SA *et. al.* , *Science* , **296** , 92 (2002).
Sasaki T *et. al.* , *Nature* , **420** , 312 (2002).

***Saccharum officinarum* (So)**
Project/s:
GenBank - NIH genetic sequence database: (http://www.ncbi.nlm.nih.gov/BLAST/)
The Institute for Genomic Research: TIGR Saccharum officinarum Gene Index (http://www.tigr.org/tigr-scripts/tgi/T_index.cgi?species=s_officinarum)
Publication/s:
Vettore AL *et. al.* , *Genome Res* , **13** , 2725 (2003).

***Sorghum bicolor* (Sob)**
Project/s:
The Institute for Genomic Research: TIGR Sorghum bicolor Gene Index (http://www.tigr.org/tigr-scripts/tgi/T_index.cgi?species=sorghum)

***Zea mays* (Zm)**
Project/s:
GenBank - NIH genetic sequence database: (http://www.ncbi.nlm.nih.gov/BLAST/)
The Institute for Genomic Research: TIGR Maize (Zea mays) Gene Index (http://www.tigr.org/tigr-scripts/tgi/T_index.cgi?species=maize)
Publication/s:
Verza NC *et. al.* , *Plant Mol Biol* , **59** , 363 (2005).
Lai J *et. al.* , *Genome Res* , **14** , 1932 (2004).

***Hordeum vulgare subsp. vulgare* (Hv)**
Project/s:
GenBank - NIH genetic sequence database: (http://www.ncbi.nlm.nih.gov/BLAST/)
The Institute for Genomic Research: TIGR Barley (Hordeum vulgare) Gene Index (http://www.tigr.org/tigr-scripts/tgi/T_index.cgi?species=barley)
Publication/s:
Zhang H *et. al.* , *Plant J* , **40** , 276 (2004).

Triticum aestivum **(Ta)**
Project/s:
The Institute for Genomic Research: TIGR Wheat (Triticum aestivum) Gene Index (http://www.tigr.org/tigr-scripts/tgi/T_index.cgi?species=wheat)

Beta vulgaris **(Bev)**
Project/s:
GenBank - NIH genetic sequence database: (http://www.ncbi.nlm.nih.gov/BLAST/)
The Institute for Genomic Research: TIGR Beta vulgaris Gene Index (http://www.tigr.org/tigr-scripts/tgi/T_index.cgi?species=beet)
Publication/s:
Herwig R *et. al.* , *Plant J* , **32** , 845 (2002).

Petroselinum crispum **(Pec)**

Helianthus annuus **(Hea)**
Project/s:
GenBank - NIH genetic sequence database: (http://www.ncbi.nlm.nih.gov/BLAST/)
The Institute for Genomic Research: TIGR Helianthus annuus Gene Index (http://www.tigr.org/tigr-scripts/tgi/T_index.cgi?species=sunflower)
Publication/s:
Ben C *et. al.* , *Plant Mol Biol* , **57** , 255 (2005).

Lactuca sativa **(Las)**
Project/s:
The Institute for Genomic Research: TIGR Lactuca sativa Gene Index (http://www.tigr.org/tigr-scripts/tgi/T_index.cgi?species=lettuce)

Capsicum annuum **(Caa)**
Project/s:
The Institute for Genomic Research: TIGR Capsicum annuum Gene Index (http://www.tigr.org/tigr-scripts/tgi/T_index.cgi?species=pepper)
The Institute for Genomic Research: TIGR Pepper Gene Index (http://www.tigr.org/tigr-scripts/tgi/T_index.cgi?species=pepper)

Nicotiana tabacum **(Nt)**
Project/s:
North Carolina State University: Tobacco Genome Initiative (TGI) (http://tgi.ncsu.edu/index.html)
The Institute for Genomic Research: TIGR Nicotiana tabacum Gene Index (http://www.tigr.org/tigr-scripts/tgi/T_index.cgi?species=tobacco)

Solanum tuberosum **(St)**
Project/s:
The Institute for Genomic Research: TIGR Potato (Solanum tuberosum) Gene Index (http://www.tigr.org/tigr-scripts/tgi/T_index.cgi?species=potato)

Solanum habrochaites **(Soh)**

Lycopersicon esculentum **(Le)**
Project/s:
GenBank - NIH genetic sequence database: (http://www.ncbi.nlm.nih.gov/BLAST/)
The Institute for Genomic Research: TIGR Tomato (Lycopersicon esculentum) Gene Index (http://www.tigr.org/tigr-scripts/tgi/T_index.cgi?species=tomato)
Publication/s:
Yamamoto N *et. al.* , *Gene* , **356** , 127 (2005).

Lotus japonicus **(Lcj)**
Project/s:
GenBank - NIH genetic sequence database: (http://www.ncbi.nlm.nih.gov/BLAST/)
GenBank - NIH genetic sequence database: (http://www.ncbi.nlm.nih.gov/BLAST/)
Kazusa DNA Research Institute: Lotus japonicus EST index (http://www.kazusa.or.jp/en/plant/lotus/EST/)
Legume base: Lotus japonicus (http://shigen.lab.nig.ac.jp/legume/legumebase/index.jsp)
The Institute for Genomic Research: TIGR Lotus japonicus Gene Index (http://www.tigr.org/tigr-scripts/tgi/T_index.cgi?species=l_japonicus)
Publication/s:
Asamizu E *et. al.* , *Plant Mol Biol* , **54** , 405 (2004).
Kato T *et. al.* , *DNA Res* , **10** , 277 (2003).
Asamizu E *et. al.* , *DNA Res* , **10** , 115 (2003).
Kaneko T *et. al.* , *DNA Res* , **10** , 27 (2003).
Nakamura Y *et. al.* , *DNA Res* , **9** , 63 (2002).
Sato S *et. al.* , *DNA Res* , **8** , 311 (2001).
Asamizu E *et. al.* , *DNA Res* , **7** , 127 (2000).

Glycine max **(Glm)**
Project/s:
Legume base: Glycine max (http://shigen.lab.nig.ac.jp/legume/legumebase/index.jsp)
The Institute for Genomic Research: TIGR Soybean (Glycine max) Gene Index (http://www.tigr.org/tigr-scripts/tgi/T_index.cgi?species=soybean)

Phaseolus vulgaris **(Phv)**
Project/s:
GenBank - NIH genetic sequence database: (http://www.ncbi.nlm.nih.gov/BLAST/)
The Institute for Genomic Research: TIGR Phaseolus vulgaris Gene Index (http://www.tigr.org/tigr-scripts/tgi/T_index.cgi?species=Common_bean)
Publication/s:
Ramirez M *et. al.* , *Plant Physiol* , **137** , 1211 (2005).

Medicago truncatula **(Mt)**
Project/s:
The Institute for Genomic Research: TIGR Medicago truncatula Gene Index (http://www.tigr.org/tigr-scripts/tgi/T_index.cgi?species=medicago)

Ricinus communis **(Ric)**

Populus trichocarpa **(Pot)**
Project/s:
DOE Joint Genome Institute: Populus trichocarpa (http://genome.jgi-psf.org/Poptr1/Poptr1.home.html)
International Species Sequencing Consortium: Populus trichocarpa Sequencing Consortium ()
PopulusDB: PopulusDB (http://poppel.fysbot.umu.se/index.html)
The Institute for Genomic Research: TIGR Populus Gene Index (http://www.tigr.org/tigr-scripts/tgi/T_index.cgi?species=poplar)
Publication/s:
Tuskan GA *et. al.* , *Science* , **313** , 1596 (2006).
Sterky F *et. al.* , *Proc Natl Acad Sci U S A* , **101** , 13951 (2004).

Arabidopsis thaliana **(At)**
Project/s:
GenBank - NIH genetic sequence database: (http://www.ncbi.nlm.nih.gov/BLAST/)
International Species Sequencing Consortium: Arabidopsis thaliana Sequencing Consortium ()
Kazusa DNA Research Institute: Arabidopsis thaliana EST Index (http://www.kazusa.or.jp/en/plant/arabi/EST/index.html)
RIKEN Arabidopsis Genome Encyclopedia: (http://rarge.gsc.riken.jp/index.html)
The Institute for Genomic Research: TIGR Arabidopsis thaliana Gene Index (http://www.tigr.org/tigr-scripts/tgi/T_index.cgi?species=arab)
The Institute for Genomic Research: The TIGR Arabidopsis thaliana Database (http://www.tigr.org/tdb/e2k1/ath1/)
Publication/s:
Alexandrov NN *et. al.* , *Plant Mol Biol* , **60** , 69 (2006).
Schmid KJ *et. al.* , *Genome Res* , **13** , 1250 (2003).
Seki M *et. al.* , *Science* , **296** , 141 (2002).
Salanoubat M *et. al.* , *Nature* , **408** , 820 (2000).

Arabidopsis Genome Initiative , *Nature* , **408** , 796 (2000).
Tabata S *et. al.* , *Nature* , **408** , 823 (2000).
Theologis A *et. al.* , *Nature* , **408** , 816 (2000).
Asamizu E *et. al.* , *DNA Res* , **7** , 175 (2000).
Lin X *et. al.* , *Nature* , **402** , 761 (1999).
Mayer K *et. al.* , *Nature* , **402** , 769 (1999).

***Brassica napus* (Bn)**
Project/s:
The Institute for Genomic Research: TIGR Oilseed rape (Brassica napus) Gene Index (http://www.tigr.org/tigr-scripts/tgi/T_index.cgi?species=oilseed_rape)

***Brassica rapa subsp. pekinensis* (Brsp)**
Project/s:
John Innes Centre: Brassica Genome Gateway (http://brassica.bbsrc.ac.uk/)

***Gossypium raimondii* (Gor)**

***Gossypium hirsutum* (Gh)**

***Vitis vinifera* (Vv)**
Project/s:
GenBank - NIH genetic sequence database: (http://www.ncbi.nlm.nih.gov/BLAST/)
The Institute for Genomic Research: TIGR Vitis vinifera Gene Index (http://www.tigr.org/tigr-scripts/tgi/T_index.cgi?species=grape)
Publication/s:
Moser C *et. al.* , *Funct Integr Genomics* , **5** , 208 (2005).

***Aquilegia* (Aq)**
Project/s:
The Institute for Genomic Research: TIGR Aquilegia Gene Index (http://www.tigr.org/tigr-scripts/tgi/T_index.cgi?species=aquilegia)

***Phaeodactylum tricornutum CCAP1055/1* (Pht)**
Project/s:
DOE Joint Genome Institute: Phaeodactylum tricornutum (http://genome.jgi-psf.org/Phatr1/Phatr1.home.html)

***Thalassiosira pseudonana CCMP1335* (Thp)**
Project/s:
DOE Joint Genome Institute: Thalassiosira pseudonana (http://genome.jgi-psf.org/thaps1/thaps1.home.html)
International Species Sequencing Consortium: Thalassiosira pseudonana Sequencing Consortium ()
National Center for Biotechnology Information: NCBI Protozoa genomes (http://www.ncbi.nlm.nih.gov/sutils/blast_table.cgi?taxid=Protozoa)
Publication/s:
Armbrust EV *et. al.* , *Science* , **2004** , 79 (2004).

***Hyaloperonospora parasitica* (Hyp)**
Project/s:
The Genome Sequencing Center at Washington University: Hyaloperonospora parasitica Sequencing (http://genome.wustl.edu/data.cgi)

***Phytophthora parasitica* (Pyp)**
Project/s:
GenBank - NIH genetic sequence database: (http://www.ncbi.nlm.nih.gov/BLAST/)
Publication/s:
Panabieres F *et. al.* , *Fungal Genet Biol* , **42** , 611 (2005).

***Phytophthora ramorum Pr102* (Phr)**
Project/s:
DOE Joint Genome Institute: Phytophthora ramorum (http://genome.jgi-psf.org/ramorum1/ramorum1.home.html)
National Center for Biotechnology Information: NCBI Eukaryotic Genomes Project (http://www.ncbi.nlm.nih.gov/sutils/genom_table.cgi)
Publication/s:
Tyler BM *et. al.* , *Science* , **313** , 1261 (2006).

***Phytophthora sojae P6497* (Phs)**
Project/s:
DOE Joint Genome Institute: Phytophthora sojae (http://genome.jgi-psf.org/sojae1/sojae1.home.html)
National Center for Biotechnology Information: NCBI Eukaryotic Genomes Project (http://www.ncbi.nlm.nih.gov/sutils/genom_table.cgi)
Publication/s:
Tyler BM *et. al.* , *Science* , **313** , 1261 (2006).

***Phytophthora infestans T30-4* (Phi)**
Project/s:
Broad Institute of Harvard and MIT: Phytophthora infestans Database (http://www.broad.mit.edu/annotation/genome/phytophthora_infestans/Home.html)
GenBank - NIH genetic sequence database: (http://www.ncbi.nlm.nih.gov/BLAST/)
The Wellcome Trust Sanger Institute: Phytophthora infestans BAC Sequencing (http://www.sanger.ac.uk/Projects/P_infestans/)
Publication/s:
Randall TA *et. al.* , *Mol Plant Microbe Interact* , **18** , 229 (2005).
